# Reactions of an anionic chelate phosphane/borata-alkene ligand with [Rh(nbd)Cl]_2_, [Rh(CO)_2_Cl]_2_ and [Ir(cod)Cl]_2_†
†Dedicated to Professor Wolfgang Kirmse on the occasion of his 90th birthday.
‡
‡Electronic supplementary information (ESI) available: Additional experimental details, further spectral and crystallographic data. CCDC deposition numbers are 1960302–1960306 and 2008240. For ESI and crystallographic data in CIF or other electronic format see DOI: 10.1039/d0sc02223c


**DOI:** 10.1039/d0sc02223c

**Published:** 2020-06-19

**Authors:** Kohei Watanabe, Atsushi Ueno, Xin Tao, Karel Škoch, Xiaoming Jie, Sergei Vagin, Bernhard Rieger, Constantin G. Daniliuc, Matthias C. Letzel, Gerald Kehr, Gerhard Erker

**Affiliations:** a Organisch-Chemisches Institut , Westfälische Wilhelms-Universität Münster , Corrensstraße 40 , 48149 Münster , Germany . Email: erker@uni-muenster.de; b Wacker-Lehrstuhl für Makromolekulare Chemie , Fakultät für Chemie , Technische Universität München , Lichtenbergstraße 4, 85747 Garching bei München , Germany

## Abstract

Borata-alkenes can serve as anionic olefin equivalent ligands in transition metal chemistry. A chelate ligand of this type is described and used for metal coordination. Deprotonation of the Mes_2_P(CH_2_)_2_B(C_6_F_5_)_2_ frustrated Lewis pair in the α-CH[B] position gave the methylene-bridged phosphane/borata-alkene anion. It reacted with the [Rh(nbd)Cl] or [Rh(CO)_2_Cl] dimers to give the respective neutral chelate [P/C=B][Rh] complexes. The reaction of the [P/C=B]^–^ anion with [Ir(cod)Cl]_2_ proceeded similarly, only that the complex underwent a subsequent oxidative addition reaction at the mesityl substituent. Both the resulting Ir(iii)hydride complex **15** and the P/borata-alkene Rh system **12** were used as hydrogenation catalysts. The [P/C=B(C_6_F_5_)_2_]Rh(nbd) complex **12** served as a catalyst for arylacetylene polymerization.

## Abstract 2

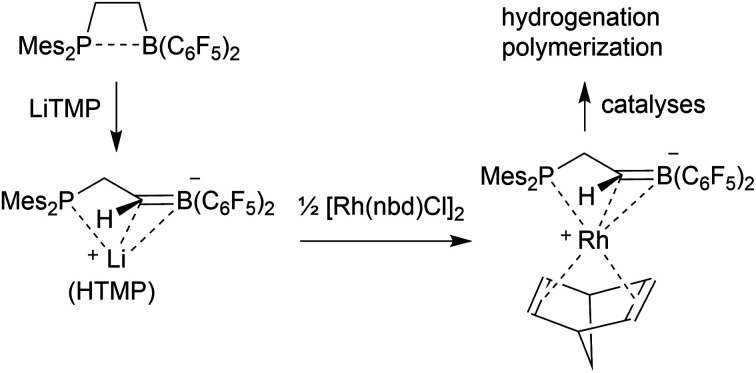
Borata-alkenes can serve as anionic olefin equivalent ligands in transition metal chemistry. A chelate ligand of this type is described and used for metal coordination.

## Introduction

Carbanions in the α-position to boryl groups show a conjugative interaction with the adjacent Lewis acid. Such systems can be described as borata-alkenes. Borata-alkenes derived from some alkyldiarylboranes had previously been prepared.^1^ Typically, short C=B bond lengths around 1.45 Å were found in these systems. In addition, a variety of related boryl-carbanion ↔ borata-alkene systems were *in situ* generated and employed as reagents *e.g.* in borata-Wittig olefination chemistry.^2^ These reactions are the formal boron analogues of the conventional phosphorus ylide derived Wittig olefination reaction of organic carbonyl compounds.^3^

It was recently shown that the presence of the strongly electron-withdrawing –B(C_6_F_5_)_2_ group resulted in a markedly increased α-CH acidity in the respective boranes. A DFT study had revealed that *e.g.* H_3_C–B(C_6_F_5_)_2_ showed a p*K*_a_-value comparable to that of cyclopentadiene.^4^ According to this study the H_3_C–B(C_6_F_5_)_2_ borane must be considered >10 p*K*_a_ values more C–H acidic than the related H_3_C-BMes_2_ borane. Consequently, R–H_2_C–B(C_6_F_5_)_2_ systems were easily deprotonated to give the corresponding [R–HC=B(C_6_F_5_)_2_]^–^ borata-alkene systems. Several of such systems were isolated as their Li^+^ salts. Some were used in borata-Wittig olefination reactions.^5^

**Scheme 1:**
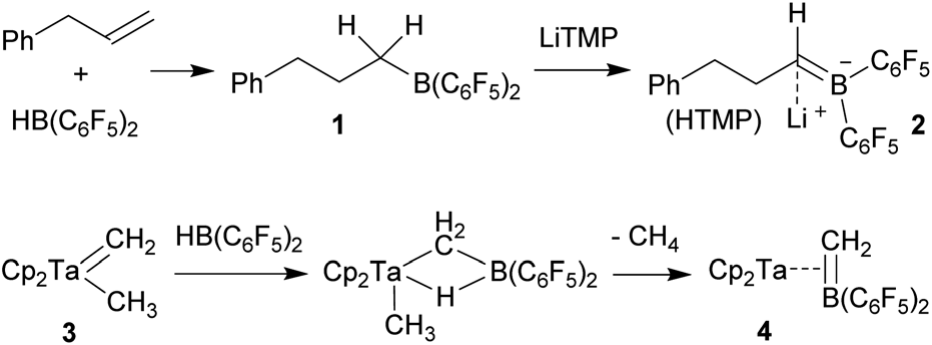
Formation of borata-alkene derivatives containing the =B(C_6_F_5_)_2_ unit.

Neutral bora-alkene compounds had previously been used as ligands^6^ and there are reports about the use of borata-benzenes in organometallic chemistry.^7^ There are a few examples of η^3^-borata-allyl metal complexes and related systems known.^8^ Piers *et al.* had prepared the borata-alkene tantalocene complex **4** (Scheme 1)^9^ and emphasized the relation of the anionic η^2^-[H_2_C=B(C_6_F_5_)_2_]^–^ ligand with the neutral η^2^-olefin analogues. The Piers group developed some follow-up chemistry of complex **4**.^9^ C. Martin *et al.* have just recently described a conceptually related borata-phenanthrene gold complex.^10^,^11^

Formal substitution of a hydrogen atom of the borata-alkene =CH_2_– terminus by a Mes_2_P–CH_2_-substituent now gave an anionic [P/C=B] system that served as a chelate ligand in Rh and Ir coordination chemistry.^12^ The preparation of first examples of this class of compounds and some uses are described in this account.

## Results and discussion

### Development of the chelate phosphane/borata-alkene ligand system

We started our phosphane/borata-alkene chelate ligand synthesis from the ethylene-bridged frustrated P/B Lewis pair (FLP) **7**.^13^ This was obtained from the hydroboration reaction of Mes_2_P-vinyl (**5**) with Piers' borane [HB(C_6_F_5_)_2_] (**6**)^14^ as we had previously reported.^15^

**Scheme 2:**
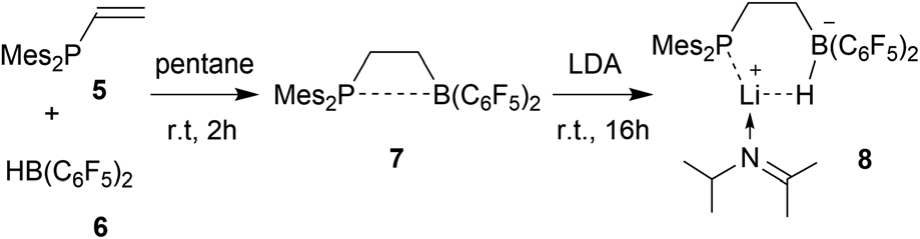
Reaction of the FLP **7** with LDA.

**Fig. 1:**
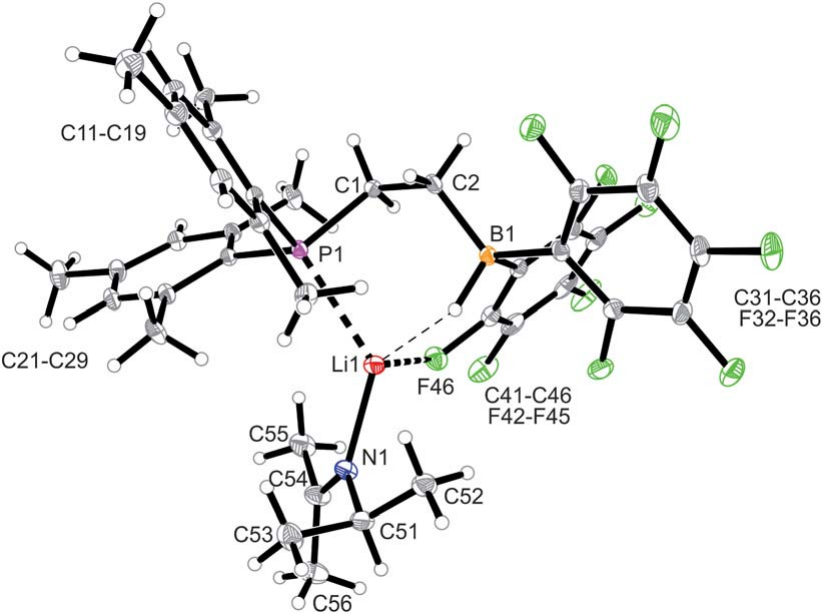
Molecular structure of compound **8** (thermal ellipsoids at 15% probability). Selected bond lengths (Å) and angles (°): P1–Li1 2.536(6) F46–Li1, 2.051(7), N1–C51 1.475(5), N1–C54 1.297(5), C2–B1–C31 115.8(3), C2–B1–C41 108.3(3), C31–B1–C41 109.1(3).

We first attempted deprotonation of **7** at the α-position to the boron atom by treatment with LDA (r.t., pentane, 16 h). Compound **7** is α-CH acidic, but it is also an active boron Lewis acid that is able to abstract hydride from amines in the α-position to nitrogen with iminium salt formation.^16^ We found that a variant of the latter reaction is favoured in this system. Hydride abstraction from an isopropyl substituent of the LDA reagent by the borane Lewis acid functional group of **7** generated the respective imine. This is found as a component in the product **8** that we isolated from the reaction mixture as a white solid in 67% yield (Scheme 2). Compound **8** was characterized by an X-ray crystal structure analysis (Fig. 1). It shows the intact Mes_2_PCH_2_CH_2_B(C_6_F_5_)_2_ backbone. The boron atom shows a pseudotetrahedral coordination geometry (ΣB1^CCC^ 333.2°); it has hydride attached. The lithium cation shows contacts to the [B]-H moiety, the phosphorus atom and one *ortho*-C_6_F_5_ fluorine atom. The Li cation has the newly formed imine moiety N-coordinated. In solution compound **8** shows a ^11^B NMR [B]-H doublet at *δ* –18.4 with a ^1^*J*_BH_ ∼70 Hz coupling constant and a ^7^Li NMR (C_6_D_6_) signal at *δ* 1.4. The –N=CMe_2_ imine ^13^C NMR resonance occurs at *δ* 173.5.

**Scheme 3:**
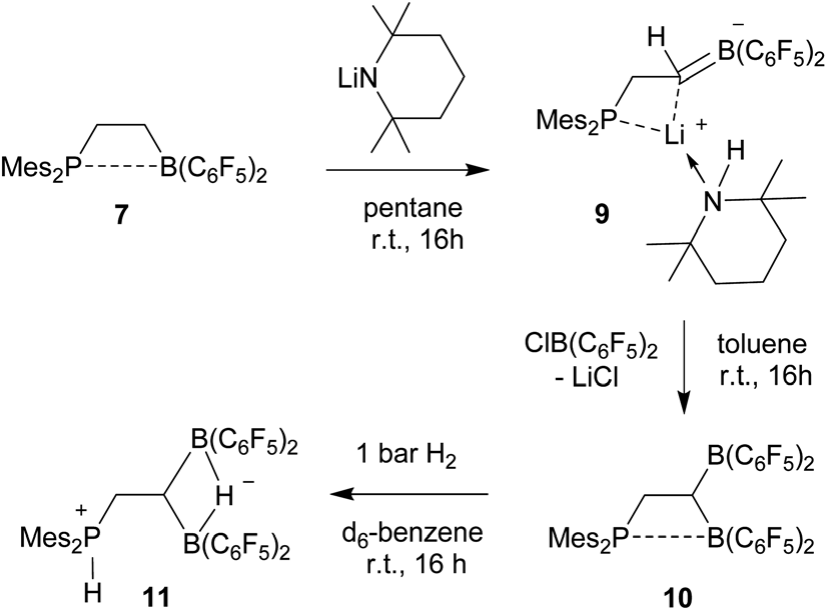
Formation and borylation reaction of the borata-alkene **9**.

In order to avoid the unwanted N–CH hydride abstraction we reacted the FLP **7** with the LiTMP reagent, a base that has no CH groups α to nitrogen. The reaction was carried out with the *in situ* generated FLP **7**. Treatment with LiTMP in pentane for 16 h at room temperature followed by workup gave the methylene-linked phosphane/borata-alkene product **9** (Scheme 3), that we isolated as a white solid in 78% yield.

**Fig. 2:**
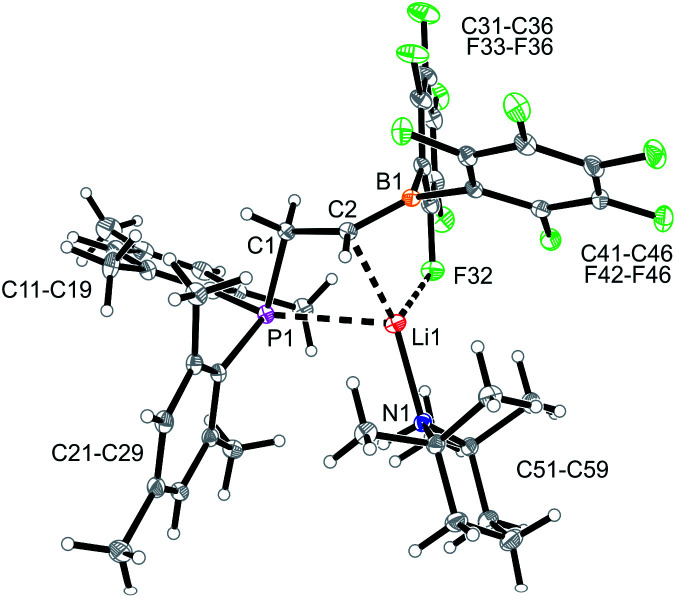
A view of the molecular structure of the phosphane/borata-alkene system **9** (thermal ellipsoids at 15% probability). Selected bond lengths (Å) and angles (°): B1–C2 1.441(3), B1–C31 1.607(3), B1–C41 1.602(3), B1–Li1 2.654(5), C1–P1 1.855(2), C2–Li1 2.367(4), F32–Li1 2.076(4), B1–C2–Li1 84.6 (2), B1–C2–C1 126.4(2), B1–Li1–C2 32.7(1), C2–B1–Li1 62.7(2), C2–B1–C31 120.7(2), C2–B1–C41 123.0(2), C31–B1–C41 116.3(2).

Compound **9** was characterized by C,H,N elemental analysis, by spectroscopy and by X-ray diffraction. The X-ray crystal structure analysis confirmed the formation of the borata-alkene functionality. It shows the typical short B1–C2 linkage of 1.441(3)Å, which is much shorter than the adjacent boron-aryl bonds (B1–C31: 1.607(3)Å, B1–C41: 1.602(3)Å). The boron coordination geometry in compound **9** is trigonal-planar (ΣB1^CCC^ 360.0°). The B1–C2–C1 angle amounts to 126.4(2)°. The lithium ion in **9** shows contacts to the borata-alkene unit as well as to the phosphorus atom and one *ortho*-C_6_F_5_ fluorine atom. The lithium atom Li^+^ also has the HTMP amine ligand bonded to it that had been formed in the deprotonation process (Fig. 2).

In solution (THF-d_8_) compound **9** features a typical borata-alkene ^11^B NMR signal at *δ* 18.6. The ^31^P NMR signal is at *δ* –20.6 and the –CH_2_–CH= backbone shows ^1^H NMR resonances at *δ* 4.21 (BCH; ^13^C: *δ* 106.7 (br)) and *δ* 3.36 (CH_2_; ^13^C: *δ* 33.5, ^1^*J*_PC_ = 16.0 Hz). The ^19^F NMR spectrum of compound **9** shows two sets of *o*,*p*,*m*-resonances of the C=B(C_6_F_5_)_2_ moiety (*E* and *Z* to the alkyl group at the adjacent sp^2^-hybridized borata-alkene carbon atom C2).

We briefly investigated the nucleophilic property of the borata-alkene unit in compound **9**. For that purpose, we reacted it with the ClB(C_6_F_5_)_2_ reagent.^14^,^17^ The reaction (in toluene, r.t., 16 h) resulted in a substitution reaction at boron to give the P/B/B compound **10** (isolated as a yellow solid in 52% yield). It was characterized by C,H-elemental analysis, by spectroscopy and by its reaction with dihydrogen (see below). Compound **10** is a typical intramolecular FLP, showing a P–B interaction with one boron atom and having the other one free. However, the temperature dependent ^19^F NMR spectrum showed exchange between the pair of B(C_6_F_5_)_2_ groups at *e.g.* 299 K. Only at low temperature (*e.g.* 203 K) we observed a set of three broad ^19^F NMR resonances of a free trigonal planar B(C_6_F_5_)_2_ unit and a set of ten separate signals [four *ortho*, two *para* and four *meta*] of the rotationally hindered P···B(C_6_F_5_)_2_ group. The ^31^P NMR (299 K) signal of compound **10** is at *δ* 16.3 and the –CH_2_–CH= backbone shows ^1^H NMR features at *δ* 3.55 and *δ* 4.30, respectively (^13^C: *δ* 29.2, 38.1 (br)).

**Fig. 3:**
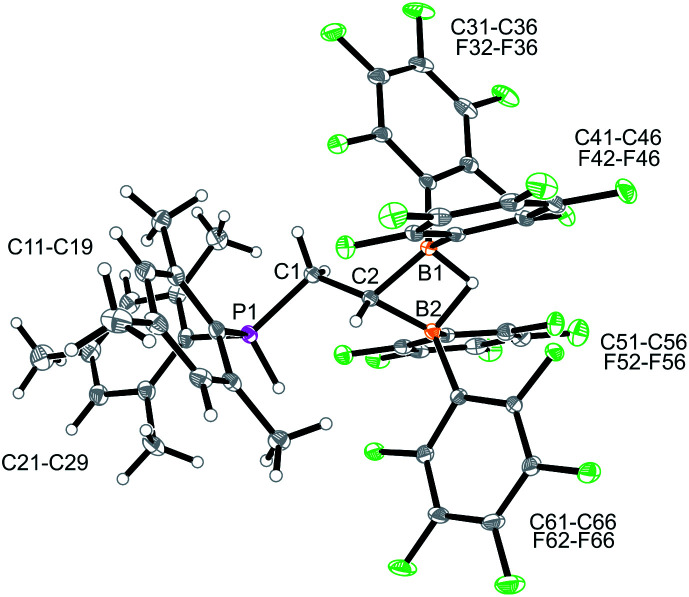
Molecular structure of the P/B/B dihydrogen splitting product **11** (thermal ellipsoids at 30% probability). Selected bond lengths (Å) and angles (°): B1–C2 1.601(3), B2–C2 1.601(3), C1–C2 1.524(3), C1–P1–C11 107.3(1), C1–P1–C21 121.1(1), C11–P1–C21 111.3(1).

Compound **10** reacted rapidly with dihydrogen under mild conditions (d_6_-benzene, r.t., 16 h, 1 bar H_2_) to give the phosphonium/hydridoborate dihydrogen splitting product **11** (isolated as a solid in 71% yield). The X-ray crystal structure analysis (Fig. 3) showed the presence of the phosphonium unit (ΣP1^CCC^ 339.7°) and the newly formed hydride-bridged bis-borane moiety. In solution (CD_2_Cl_2_) the phosphonium [P]-H unit showed up at *δ* 7.58 (^1^H NMR) and *δ* –3.7 (^31^P, ^1^*J*_PH_ ∼ 480 Hz), respectively. We recorded a broadened ^11^B NMR signal at *δ* –18.1 with a corresponding broad ^1^H NMR [B](μ-H) feature at *δ* 5.45. The ^19^F NMR spectrum of compound **11** shows two equal-intensity sets of *o*,*p*,*m*-C_6_F_5_ signals of the pair of B(C_6_F_5_)_2_ groups and we observed the ^1^H/^13^C NMR signals of the –CH_2_–CH backbone at *δ* 2.84/27.8 (PCH_2_) and *δ* 1.80/7.6(br)(BCH), respectively.

### Synthesis and characterization of the P/C=B chelate metal complexes

**Scheme 4:**
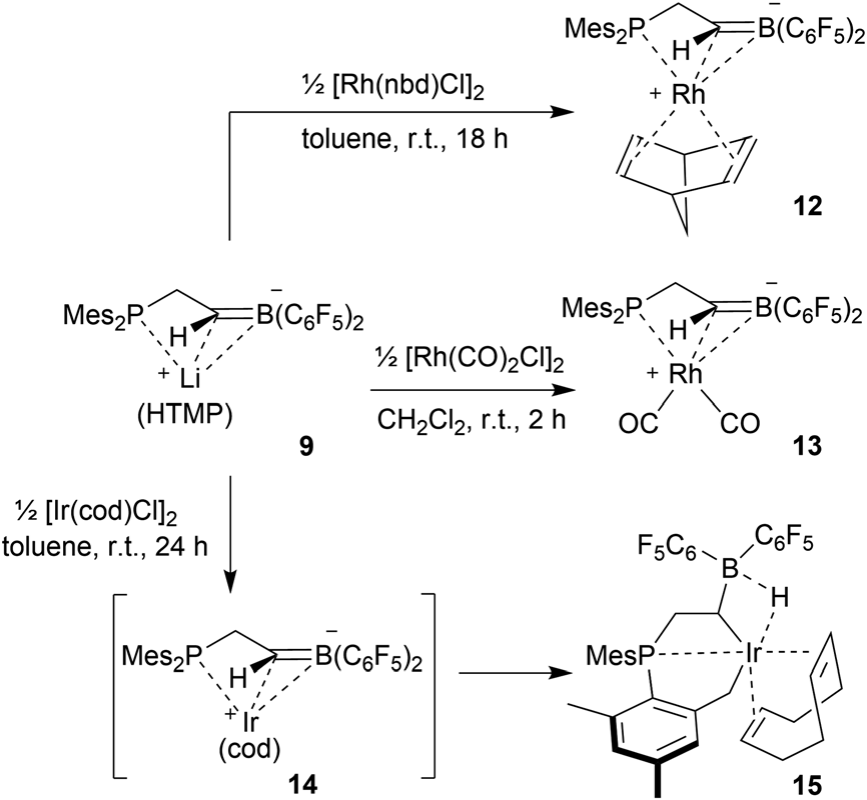
Preparation of Rh and Ir complexes from the anion **9**.

**Fig. 4:**
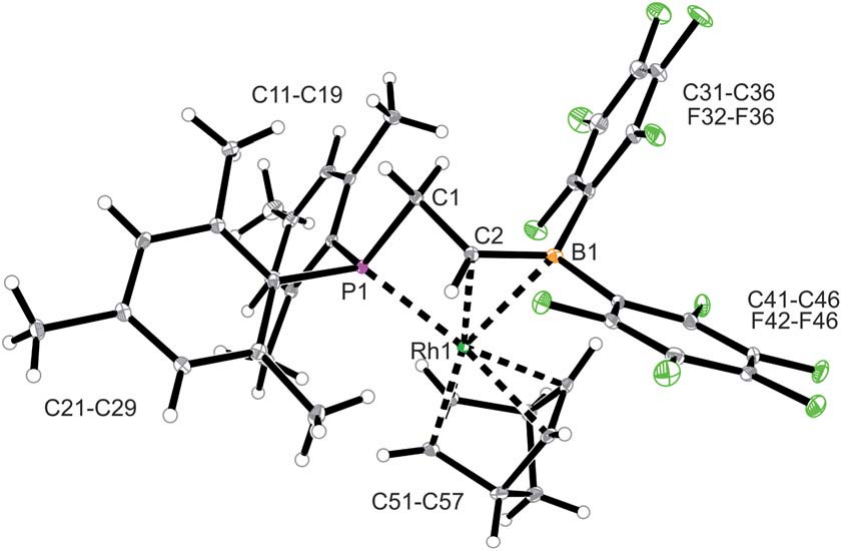
A view of the molecular structure of the chelate phosphane/borata-alkene Rh complex **12** (thermal ellipsoids at 30% probability).

We used the methylene-bridged phosphane/borata-alkene anion of the lithium salt **9** as a chelate ligand in Rh chemistry. For that purpose, we treated the (norbornadiene)RhCl dimer with the prefabricated borata-alkene reagent **9** for 18 h in toluene solution at room temperature. Workup then gave the respective neutral chelate phosphane/borata-alkene(norbornadiene)Rh complex **12** in >60% yield (Scheme 4). Suitable crystals for the X-ray crystal structure analysis were obtained from slow diffusion of pentane into a saturated solution in dichloromethane at –30 °C (Fig. 4). Compound **12** shows a distorted square-planar coordination geometry at rhodium. The P/C=B system serves as a chelate ligand. It is unsymmetrically η^2^-coordinated through both backbone atoms of the borata-alkene moiety and κP-bonded to the attached phosphanyl group. As the P/C=B ligand is mono-anionic, the resulting Rh complex is neutral. The C2–B1 bond is only marginally elongated, it is still within the typical C=B distance of borata-alkene examples^1^,^5^ (Table 1). The metal center has both olefinic π-systems of the norbornadiene ligand bonded through its endo-face. Both olefinic units are oriented perpendicular to the mean coordination plane of the transition metal center. In complex **12** the phosphane donor exhibits a stronger structural trans-effect^18^ than the borata-alkene ligand as judged from the respective Rh–C (olefin) bond lengths [trans: Rh1–C54: 2.214(2) Å, Rh1–C55: 2.213(2) Å; cis: Rh1–C51: 2.172(2) Å, Rh1–C52: 2.162(2) Å, see Fig. 4]

**Table 1:** A comparison of selected structural parameters of the chelate P/B complexes **12** (Rh) and **15** (Ir)^*a*^

	12 (Rh)	15 (Ir)^*b*^
M–C2	2.270(2)	2.166(4)
M–B1	2.611(2)	2.463(5)
M–P1	2.346(1)	2.328(1)
C2–B1	1.476(3)	1.545(6)
M–C51	2.172(2)	2.243(4)
M–C52	2.162(2)	2.256(4)
B1–C2–C1	124.2(2)	129.4(3)
C1–P1–M	88.6(1)	88.4(1)
ΣB1^CCC^	357.2	346.2

^*a*^Bond lengths in Å, angles in °.

^*b*^Two independent molecules, values are given for molecule A.

In solution, complex **12** shows a ^31^P NMR signal (CD_2_Cl_2_) at *δ* –89.0 with a ^1^*J*_RhP_ ∼ 120 Hz coupling constant. This changed only marginally when the spectrum of **12** was recorded in d_8_-THF solution. Compound **12** shows a ^11^B NMR signal at *δ* 24.3, a value that is similar to that of the uncomplexed borata-alkene anion **9** (see above).^5^ The ^19^F NMR spectrum of **12** shows two sets of *o*,*p*,*m*-C_6_F_5_ signals for the pair of pentafluorophenyl substituents at boron. We observed the ^1^H NMR signals of the chelate ligand backbone at *δ* 4.14/3.90 (PCH_2_) and *δ* 3.68 (B=CH–), respectively (corresponding ^13^C NMR signals at *δ* 42.9 and 59.8(br)), and there are the ^1^H/^13^C NMR signals of the coordinated norbornadiene ligand at rhodium (see the ESI‡ for details).

**Fig. 5:**
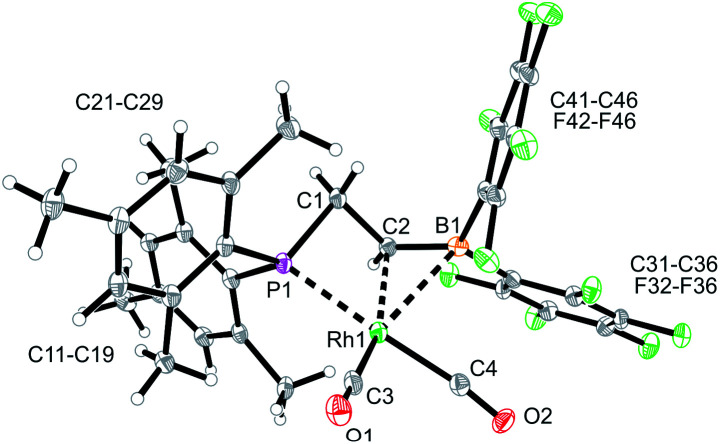
A view of the molecular structure of the chelate P/borata-alkene dicarbonyl Rh complex **13** (thermal ellipsoids at 30% probability).

The reaction of the P/borata-alkene lithium salt **9** with the chloro(dicarbonyl)Rh dimer was carried out at r.t (in dichloromethane, 2 h). Workup involving extraction with pentane and crystallization gave the neutral chelate [P/borata-alkene]Rh(CO)_2_ complex **13** as a yellow crystalline solid in 46% yield. The X-ray crystal structure analysis (Fig. 5) showed a distorted square planar coordination geometry around Rh. The phosphane (P1–Rh1: 2.335(1) Å) and the borata-alkene moiety of the chelate ligand are both bonded to rhodium (Rh1–C2: 2.251(4) Å, Rh1–B1: 2.590(5) Å). The B1–C2 linkage is found in the typical borata-alkene range at 1.476(7) Å. Again, the phosphane exerts a stronger trans effect than the C=B unit [Rh1–C4 (CO *trans* to P1): 1.915(5) Å, Rh1–C3 (CO *cis* to P1): 1.868(5) Å]. Compound **13** shows strong IR CO bands at *ν* = 2069 and 1997 cm^–1^.^19^ In CD_2_Cl_2_ solution it shows a ^11^B NMR signal at *δ* 27.3, *i.e.* in the typical borata-alkene range. The ^31^P NMR resonance was located at *δ* –105.0 with a ^1^*J*_RhP_ = 88.5 Hz coupling constant. The borata-alkene unit in complex **13** shows ^19^F NMR signals of a pair of inequivalent C_6_F_5_ substituents at boron.

The reaction between the borata-alkene reagent **9** and the iridium(cyclooctadiene)chloride dimer was carried out similarly (toluene, 24 h, r.t.). It gave a slightly different outcome. We assume that initially a (P/borata-alkene)Ir(cod) complex **14** was generated, analogous to the formation of the Rh system **12**. However, it was apparently not persistent under the prevailing reaction conditions but underwent intramolecular C–H bond activation^20^ at an *ortho*-methyl group of a mesityl substituent at phosphorus to give the oxidative addition product **15** (Scheme 4). It was isolated in 44% yield. Complex **15** was characterized spectroscopically and by X-ray diffraction (single crystals were obtained by crystallization from pentane at –30 °C).

**Fig. 6:**
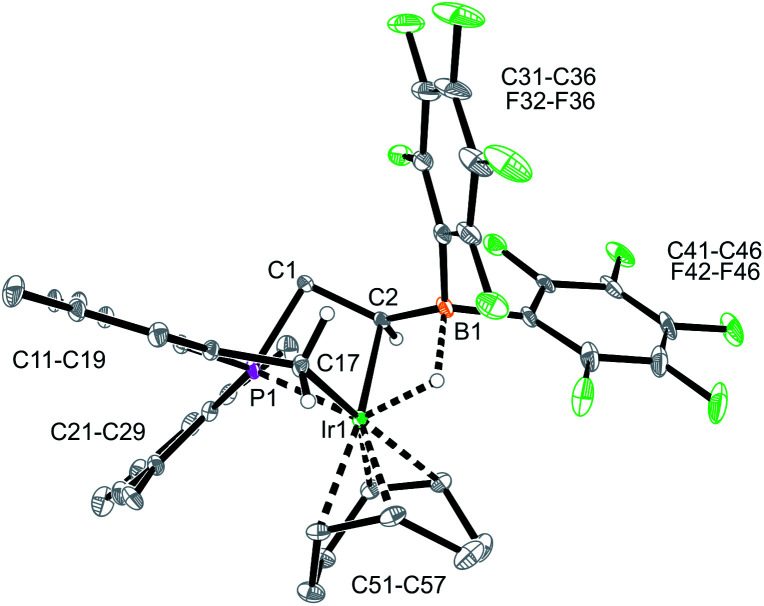
A projection of the molecular structure of the Iridium complex **15** (thermal ellipsoids at 30% probability).

The X-ray crystal structure analysis of complex **15** revealed that the iridium atom has undergone oxidative addition at a mesityl group at phosphorus, with formation of a new benzylic –CH_2_–Ir–H moiety (Fig. 6). The resulting Ir-hydride shows a contact to the boron atom. We note that the C2–B1 linkage in **15**, consequently, is much longer than in **9** or **12**, it corresponds to a short boron-carbon σ-bond. The Ir–C2 linkage is rather short (Table 1). The hydride is bridging between Ir and B [independent molecule A: Ir1A-H01 1.64(4) Å, H01–B1A 1.56(4) Å; molecule B: Ir1B–H02 1.59(4) Å, H02–B1B 1.50(4) Å] (Fig. 6).

In solution (CD_2_Cl_2_) the iridium complex **15** shows four olefinic ^1^H NMR signals of the coordinated cyclooctadiene ligand. It also features four arene CH ^1^H NMR signals of the mesitylene and the CH-activated Mes substituents at phosphorus. Complex **15** shows a broadened ^11^B NMR resonance at *δ* –17.5. The ^31^P NMR signal is observed at *δ* –104.0. It shows coupling to the Ir–H moiety (^2^*J*_PH_ ∼ 70 Hz).^21^ Consequently, the Ir-hydride signal shows up at *δ* –10.4 with *ca.* 70 Hz coupling to phosphorus (for additional details see the ESI‡).

### Catalytic reactions

**Scheme 5:**
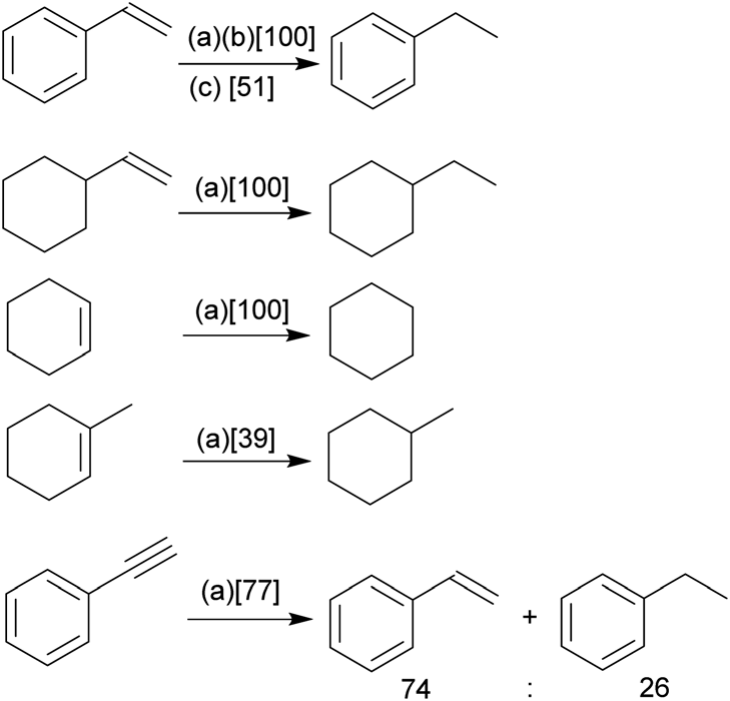
Catalytic hydrogenation of unsaturated substrates using an Ir catalyst derived from **15** under our standard conditions {1.0 bar H_2_, d_6_-benzene, r.t., 16 h, (a): 1 mol% catalyst, (b): 0.5 mol%, (c): 0.1 mol%; [% conversion achieved]}.

Our study has shown that the methylene linked phosphane/borata-alkene anion of the salt **9** served well as a chelate ligand in Rh coordination chemistry. It is likely that the Ir(iii) complex **15** was actually formed by an oxidative addition reaction at a mesityl methyl group at the stage of the analogous intermediate **14**. We carried out some preliminary investigation toward the use of the new chelate phosphane/borata-alkene complexes in catalysis. For this reason, we performed two sets of catalytic reactions using either of the complexes **12** and **15**. We first turned to alkene and alkyne hydrogenation catalysis.^22^ Exposure of complex **15** to dihydrogen (1.0 bar, r.t.) revealed the stoichiometric formation of cyclooctane, the reduction product of the cod ligand of the Ir complex **15.** Consequently, we employed compound **15** as a catalyst in our hydrogenation experiments. The hydrogenation of styrene is a typical example. With both 1 or 0.5 mol% of **15** quantitative hydrogenation to ethylbenzene was achieved (Scheme 5); with 0.1 mol% catalyst still a *ca.* 50% conversion was obtained. The catalytic hydrogenation sequence starting from complex **15** may possibly involve the not directly observed equilibration with its likely synthetic precursor **14**, the Ir(cod) analogue of the Rh complex **12** (see above).

Quantitative alkene hydrogenation was found at the **15** derived catalyst system with 1 mol% of vinylcyclohexane or cyclohexene, as well. The more sterically encumbered 1-methylcyclohexene substrate gave only a 39% conversion under these conditions and phenylacetylene eventually furnished a *ca.* 3 : 1 mixture of styrene and ethylbenzene with a combined conversion of 77% after 16 h.

**Scheme 6:**
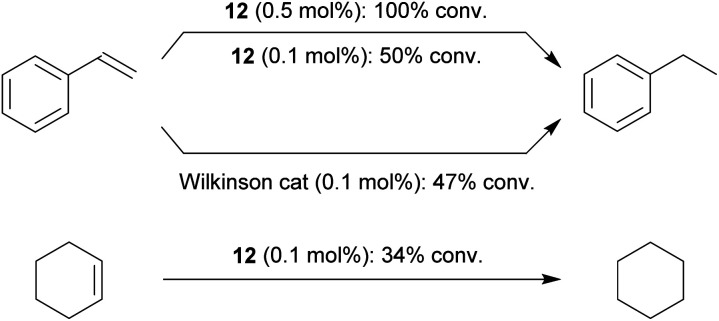
Catalytic hydrogenation of alkenes with Rh complexes: comparison of the reaction with complex **12** and the Wilkinson catalyst (1 bar H_2_, r.t., d_6_-benzene, 16 h).^23^

Styrene was quantitatively hydrogenated to ethylbenzene with 0.5 mol% of the Rh catalyst **12** under our standard conditions (Scheme 6). With 0.1 mol% a *ca.* 50% conversion was obtained, similar as with the Wilkinson catalyst under these conditions. Cyclohexene was hydrogenated at the catalyst system **12** (0.1 mol%, 34% conversion). The bulkier 1-methylcyclohexene was not hydrogenated at the Rh catalyst system **12** under our typical conditions.

So far we assume a conventional pathway of dihydrogen activation at the metal centre in the complexes **12** or **15**, but we presently cannot rule out an alternative “FLP-like” metal/borane dihydrogen splitting reaction.^24^

**Scheme 7:**
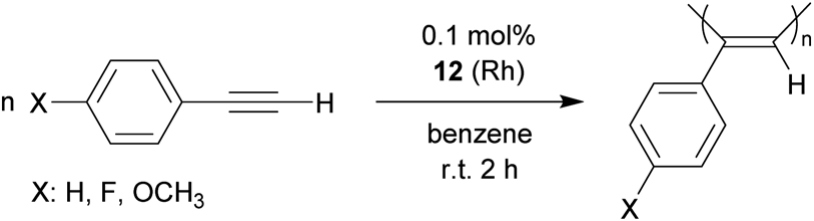
Polymerization of arylacetylenes.

A variety of Rh catalysts are able to polymerize arylacetylenes and so does the phosphane/borata-alkene complex **12**.^25^ The phenylacetylene polymerization reaction by the neutral system **12** was carried out in the non-polar solvent benzene or in ethereal solution (diethylether or tetrahydrofuran). We carried out the phenylacetylene polymerization at room temperature for a duration between 30 min (in ether) or 2 h (in benzene). With decreasing catalyst amounts (0.1 mol%, 0.05 mol%) an almost quantitative amount of polyphenylacetylene was isolated from the reaction in benzene. Even with 0.025 mol% as well as 0.01 mol% of the catalyst poly(phenylacetylene) was isolated, albeit in lower yields (45%, 28%). The obtained polymer was similar in appearance (yellow to orange solids) as the poly(phenylacetylene) obtained by Noyori *et al.* at the remotely related neutral [(Ph_3_P)_*n*_(nbd)Rh-CCPh] (*n*: 1 or 2) derived catalysts, so we assume it has a similar structure.25*a* We also polymerized *p*-fluorophenylacetylene and *p*-methoxyphenylacetylene at the catalyst system **12** (0.1 mol%) and isolated the respective polyacetylenes in close to quantitative yields (Scheme 7).^26^

Each of the arylacetylene polymers shows a single set of ^1^H NMR signals, which indicates its origin from a stereo- and regioselective polymerization process25*a* (see the ESI‡ for details). The poly(*p*-anisylacetylene) sample was characterized by MALDI-TOF mass spectrometry, which showed the regular sequence of signals separated by the mass of the respective monomer unit of 132 (depicted in the ESI‡). The molecular weights of the polyacetylene samples were determined by GPC. Under our typical conditions, the polymerization reactions in benzene or THF furnished polymers of somewhat lower molecular weight than in ether. The latter reaction produced higher molecular weight polyacetylenes.^27^ The samples contained varying amounts of insoluble material (potentially very high molecular weight polymer). For the sizable soluble fraction of the poly(*p*-anisylacetylene) sample obtained in ether with the Rh complex **12** derived catalyst we found a molecular weight of *M*_n_ ≥ 100000. The respective poly(phenylacetylene) sample had an about twice as high *M*_n_, and the poly(*p*-fluorophenylacetylene) had the highest measured *M*_n_ in the series of >400000 (Table 2). In all cases rather large polydispersities of close to 3 were found (see the ESI‡ for further details).

**Table 2:** Selected polyphenylacetylene results^*a*^

	X	Yield (%)	*M*_n_ ^*b*^	PD
1	MeO	86	114320	2.98
2	H	99	240006	2.69
3	F	97	444085	2.84

^*a*^Conditions: solvent Et_2_O (5 mL), compound **12** (0.015 mmol = 2 mol%), monomer phenylacetylenes (0.75 mmol).

^*b*^Soluble fraction measured (see the ESI for details), molecular weights determined by GPC, rel. to polystyrene standards.

## Conclusions

Our study has shown that the seminal study published by Piers *et al.* on the use of a borata-alkene as a π-ligand equivalent to ethene at an early transition metal can be substantially extended. In the Piers' system the H_2_C=B(C_6_F_5_)_2_^–^ ligand was generated by a typical organometallic reaction pathway within the coordination sphere of the metal (in that case at tantalum). Since we had found about the vastly increased α-CH acidity of the B(C_6_F_5_)_2_ boranes^4^ an improved and potentially more general pathway to κ^2^*C*,*B*-borata-alkene complexes has become evident: deprotonation^5^ of the respective suitably substituted [P]–CH_2_–CH_2_–B(C_6_F_5_)_2_ borane gave the borata-alkene in an independent initial step. Our syntheses of the methylene-bridged chelate phosphane/borata-alkene Rh and Ir complexes serve as examples of this development. The complexes are readily prepared, although the Ir system undergoes a subsequent rearrangement reaction. This new approach will probably allow for some variation on the ligand side, and it may open pathways to choosing variations on the metal side. The P/C=B ligands in the here reported complexes do not interfere with catalytic features in our examples. To us this indicates that the readily available borata-alkenes might see useful applications as polar alkene ligand analogues in organometallic and coordination chemistry as well as in catalysis.

## Conflicts of interest

There are no conflicts to declare.

## Supplementary Material

Supplementary information

Crystal structure data
