# Supplementary material for: Reactions of an anionic chelate phosphane/borata-alkene ligand with [Rh(nbd)Cl]2, [Rh(CO)2Cl]2 and [Ir(cod)Cl]2
Source: Chem Sci. 2020 Jun 19;11(28):7349–55. doi: 10.1039/d0sc02223c (PMC7654189; doi:10.1039/d0sc02223c)
Supplement: Supplementary file 1 [file SC-011-D0SC02223C-s001.pdf]

Electronic Supplementary Material (ESI) for Chemical Science.
This journal is © The Royal Society of Chemistry 2020

Reactions of an Anionic Chelate Phosphane/borata-alkene Ligand with $[Rh(nbd)Cl]_2$, $[Rh(CO)_2Cl]_2$ and $[Ir(cod)Cl]_2$.

Kohei Watanabe,[a,c] Atsushi Ueno,[a] Xin Tao,[a] Karel Škoch,[a] Xiaoming Jie,[a] Sergei Vagin,[b] Bernhard Rieger,[b] Constantin G. Daniliuc,[a] Matthias C. Letzel,[a] Gerald Kehr[a] and Gerhard Erker*[a]

[a] Organisch-Chemisches Institut, Westfälische Wilhelms-Universität Münster, Corrensstraße 40, 48149 Münster, Germany.

[b] Wacker-Lehrstuhl für Makromolekulare Chemie, Fakultät für Chemie, Technische Universität c München, Lichtenbergstraße 4, 85747 Garching bei München, Germany.

[c] Current address: Graduate School of Pharmaceutical Sciences, University of Tokyo, Hongo 7-3-1, Bunkyo-ku, Tokyo, Japan

Supporting Information

General Information	S2
Preparation of compound **8**	S3
Preparation of compound **9**	S8
Preparation of compound **10**	S13
Preparation of compound **11**	S17
Preparation of compound **12**	S21
Preparation of compound **13**	S35
Preparation of compound **15**	S39
Catalytic hydrogenation	S46
Polymerization of arylacetylenes catalyzed by Rh complex **12**	S56

General Information

All syntheses involving air- and moisture sensitive compounds were carried out using standard Schlenk-type glassware (or in a glovebox) under an atmosphere of argon. Solvents were dried and stored under an argon atmosphere. **NMR spectra** were recorded on a Varian Inova 500 (^{1}H 500 MHz, ^{13}C 126 MHz, ^{19}F 470 MHz, ^{11}B 160 MHz, ^{31}P 202 MHz, ^{7}Li 194 MHz) and on a Varian UnityPlus 600 (^{1}H 600 MHz, ^{13}C 151 MHz, ^{19}F 564 MHz, ^{11}B 192 MHz, ^{31}P 243 MHz). ^{1}H NMR and ^{13}C NMR: chemical shifts δ are given relative to TMS and referenced to the solvent signal. ^{19}F NMR: chemical shifts δ are given relative to $CFCl_3$ (external reference, δ = 0), ^{11}B NMR: chemical shifts δ are given relative to $BF_3{\cdot}Et_2O$ (external reference, δ = 0), ^{31}P NMR: chemical shifts δ are given relative to H_3PO_4 (85% in D_2O) (external reference, δ = 0). NMR assignments were supported by additional 2D-NMR experiments. Elemental analyses: Foss–Heraeus CHNO-Rapid.

Mass Spectrometry: MALDI mass spectra were recorded with an Autoflex Speed (Bruker Daltonics, Bremen). A SmartBeam™ NdYAG-Laser with 355nm wavelength was used. Trans-2-[3-(4-tert-butylphenyl)-2-methyl-2-propenylidene]malononitrile (DCTB) was used as matrix. **HRMS ESI** mass spectra were recorded on an LTQ Orbitap XL mass spectrometer (Thermo-Fisher Scientific, Bremen) equipped with nano spray ion source.

GPC analysis of polymer samples was performed at 40°C and at 1 ml/min flow rate in THF using a Varian „GPC-50 plus“ instrument equipped with PLgel-mixed-C columns (600 mm total length). The calculation of molecular weights was performed relative to polystyrene using differential refractive index detector. Polymer samples (1-2 mg in 1.3 ml THF) were gently shaken for 10 – 15 min, the undissloved material was allowed to settle or, alternatively, was filtered off, and the solutions were injected into the chromatograph in a way that each sample was in contact with THF for approx. 1 hour prior to the injection.

X-Ray diffraction: Data sets for compounds **11**, **12**, **13** and **15** were collected with a Bruker D8 Venture CMOS diffractometer. Programs used: data collection: APEX3 V2016.1-0 (Bruker AXS Inc., 2016); cell refinement: SAINT V8.37A (Bruker AXS Inc., 2015); data reduction: SAINT V8.37A (Bruker AXS Inc., 2015); absorption correction, SADABS V2014/7 (Bruker AXS Inc., 2014); structure solution *SHELXT-2015* (Sheldrick, G. M. *Acta Cryst.,* 2015, *A71*, 3-8); structure refinement *SHELXL-2015* (G. M. Sheldrick, *Acta Cryst.,* 2015, **C71** (1), 3-8). For compounds **8** and **9** data sets were collected with a Nonius Kappa CCD diffractometer. Programs used: data collection, COLLECT (R. W. W. Hooft, Bruker AXS, 2008, Delft, The Netherlands); data reduction Denzo-SMN (Z. Otwinowski, W. Minor, *Methods Enzymol.* 1997, **276**, 307-326); absorption correction, Denzo (Z. Otwinowski, D. Borek, W. Majewski, W. Minor, *Acta Crystallogr.* 2003, **A59**, 228-234); structure solution *SHELXT-2015* (G. M. Sheldrick, *Acta Cryst.,* 2015, A71, 3-8); structure refinement *SHELXL-2015* (G. M. Sheldrick, *Acta Cryst.,* 2015, **C71** (1), 3-8) and graphics, *XP* (Version 5.1, Bruker AXS Inc., Madison, Wisconsin, USA, 1998). *R*-values are given for observed reflections, and wR^2 values are given for all reflections. *Exceptions and special features*: For compound **8** one pentane molecule and for compound **9** one toluene molecule and the HTMP unit were found disordered over two positions in the asymmetric unit. Several restraints (SADI, SAME, ISOR and SIMU) were used in order to improve refinement stability. Additionally, for compound **11** a badly disordered pentane molecule and for compound **15** two badly half pentane molecules were found in the asymmetrical unit and could not be satisfactorily refined. The program SQUEEZE (A.L. Spek, Acta Cryst., 2015, **C71**, 9-18.) was therefore used to remove mathematically the effect of the solvent. The quoted

formula and derived parameters are not included the squeezed solvent molecules.

Materials: Bis(pentafluorophenyl)borane was prepared according to the procedure described in the literature. [D. J. Parks, W. E. Piers, G. P. A. Yap, *Organometallics* **1998**, *17*, 5492-5503]

Preparation of compound 8

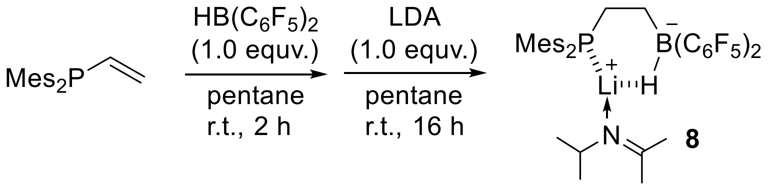

Scheme S1

A mixture of dimesitylvinylphosphine (296.4 mg, 1.0 mmol) and bis(pentafluorophenyl)borane (345.9 mg, 1.0 mmol) in *n*-pentane (10 mL) was stirred for 2 hours at room temperature. Then, lithium diisopropylamide (107.1 mg, 1.0 mmol) was added to the reaction mixture. After stirring of the obtained suspension for 16 hours, all volatiles were removed. The obtained residue was washed with *n*-pentane (3× 10 mL) and dried *in vacuo* to give compound **8** as a white powder (500 mg, 0.67 mmol, 67%).

Melting point: 150 °C

Elemental Analysis calcd for $C_{38}H_{40}BF_{10}LiNP$ (749.45 g/mol): C, 60.90; H, 5.38; N, 1.87. Found: C, 60.66; H, 5.53; N, 1.84.

^{1}H NMR (600 MHz, C_6D_6, 299 K): δ = 6.61 (m, 4H, *m*-Mes), 3.00 (m, 1H, CH^{iPr}), 2.57 (br m, 2H, PCH_2), 2.21 (s, 12H, *o*-Me^{Mes}), 2.03 (s, 6H, *p*-Me^{Mes}), 1.60 (br dm, $^3J_{PH}$ ~ 25 Hz, 2H, BCH_2), [1.42, 1.01](each s, each 3H, $Me^{N=C}$), 0.86 (d, $^3J_{HH}$ = 6.5 Hz, 6H, Me^{iPr}), n.o. (BH).

^{13}C{^{1}H} NMR (151 MHz, C_6D_6, 299 K): δ = 173.5 (N=C), 148.8 (dm, $^1J_{FC}$ ~ 230 Hz, C_6F_5), 141.6 (d, $^2J_{PC}$ = 11.0 Hz, *o*-Mes), 138.7 (dm, $^1J_{FC}$ ~ 250 Hz, C_6F_5), 138.4 (*p*-Mes), 137.5 (dm, $^1J_{FC}$ ~ 250 Hz, C_6F_5), 130.7 (d, $^3J_{PC}$ = 4.6 Hz, *m*-Mes), 131.5 (*i*-Mes), 125.3 (br, C_6F_5), 51.0 (CH^{iPr}), [28.4, 18.5]($Me^{N=C}$), 27.5 (br, PCH_2), 23.5 (d, $^3J_{PC}$ =10.8 Hz, *o*-Me^{Mes}), 22.5 (Me^{iPr}), 20.7 (*p*-Me^{Mes}), 16.1 (br, BCH_2).

^{11}B NMR (192 MHz, C_6D_6, 299 K): δ = -18.4 (d, $^1J_{BH}$ ~ 70 Hz).

^{11}B{^{1}H} NMR (192 MHz, C_6D_6, 299 K): δ = -18.4 ($ν_{1/2}$ ~ 85 Hz).

^{31}P NMR (243 MHz, C_6D_6, 299 K): δ = -23.3 ($ν_{1/2}$ ~ 60 Hz).

^{19}F NMR (564 MHz, C_6D_6, 299 K): δ = -135.6 (m, 2F, *o*-C_6F_5), -161.1 (t, $^3J_{FF}$ = 20.3 Hz, 1F, *p*-C_6F_5), -164.4 (m, 2F, *m*-C_6F_5), [$Δ^{19}F_{m,p}$ = 3.3].

^{7}Li NMR (194 MHz, C_6D_6, 299 K): δ = 1.4 ($ν_{1/2}$ ~ 35 Hz).

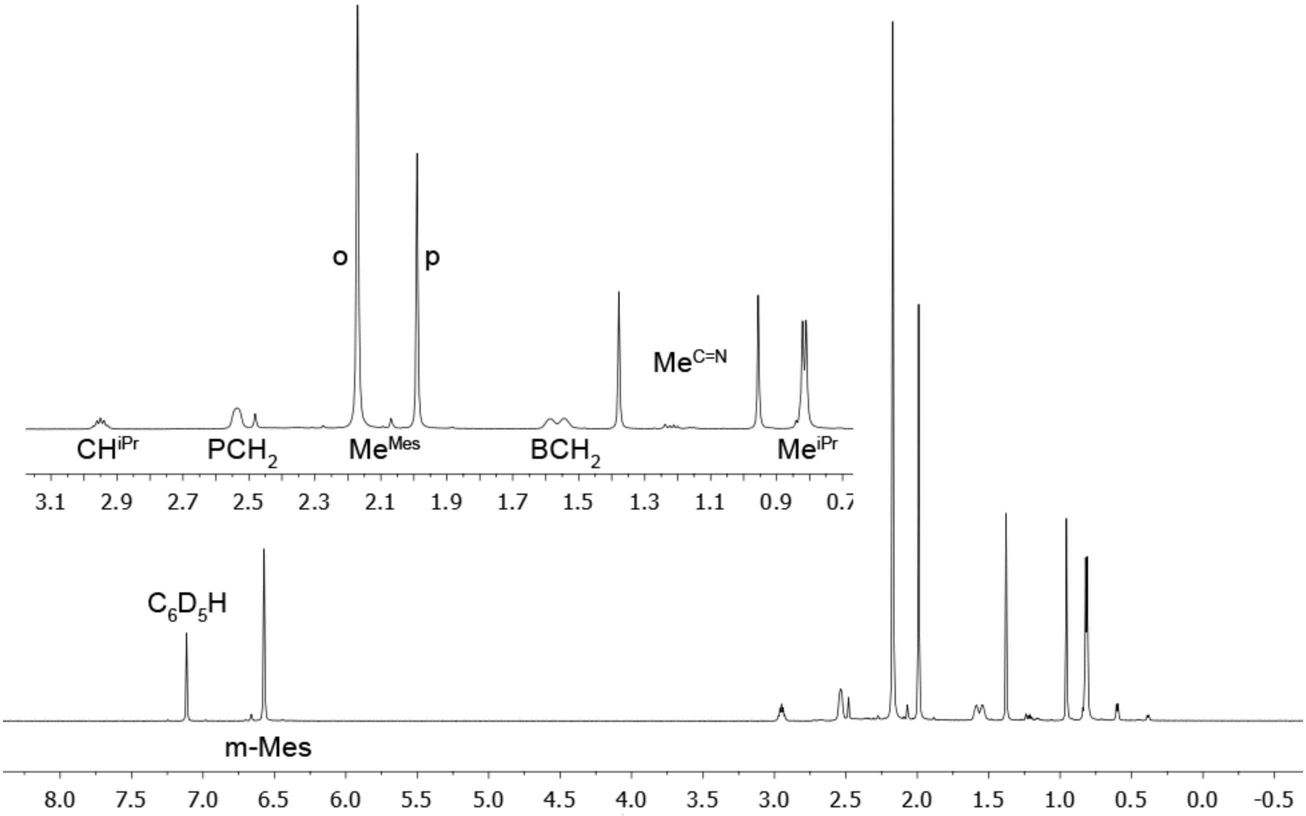

Figure S1. ^{1}**H NMR** (600 MHz, C_6D_6, 299 K) spectrum of compound **8**.

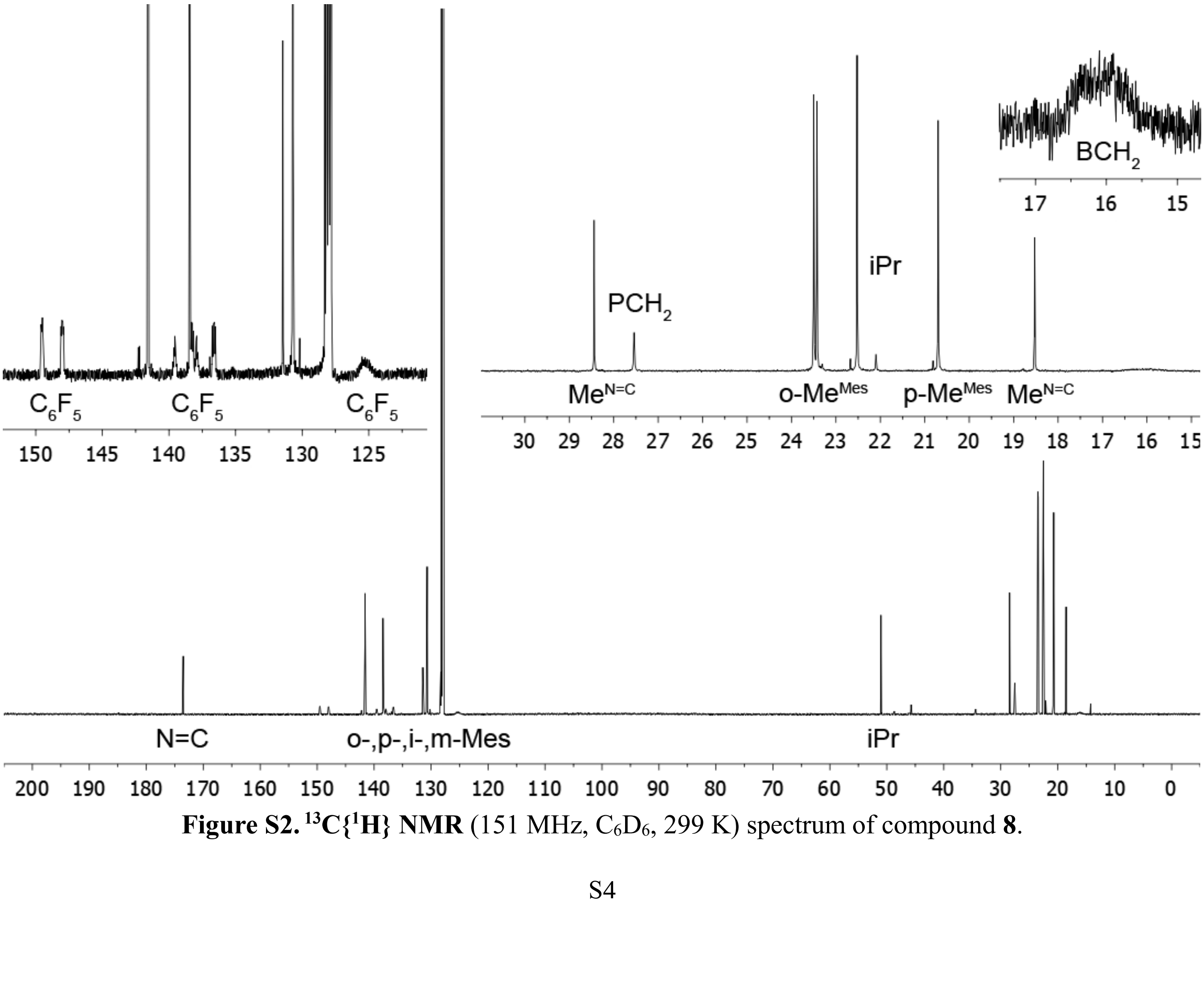

Figure S2. ^{13}**C{**^{1}**H} NMR** (151 MHz, C_6D_6, 299 K) spectrum of compound **8**.

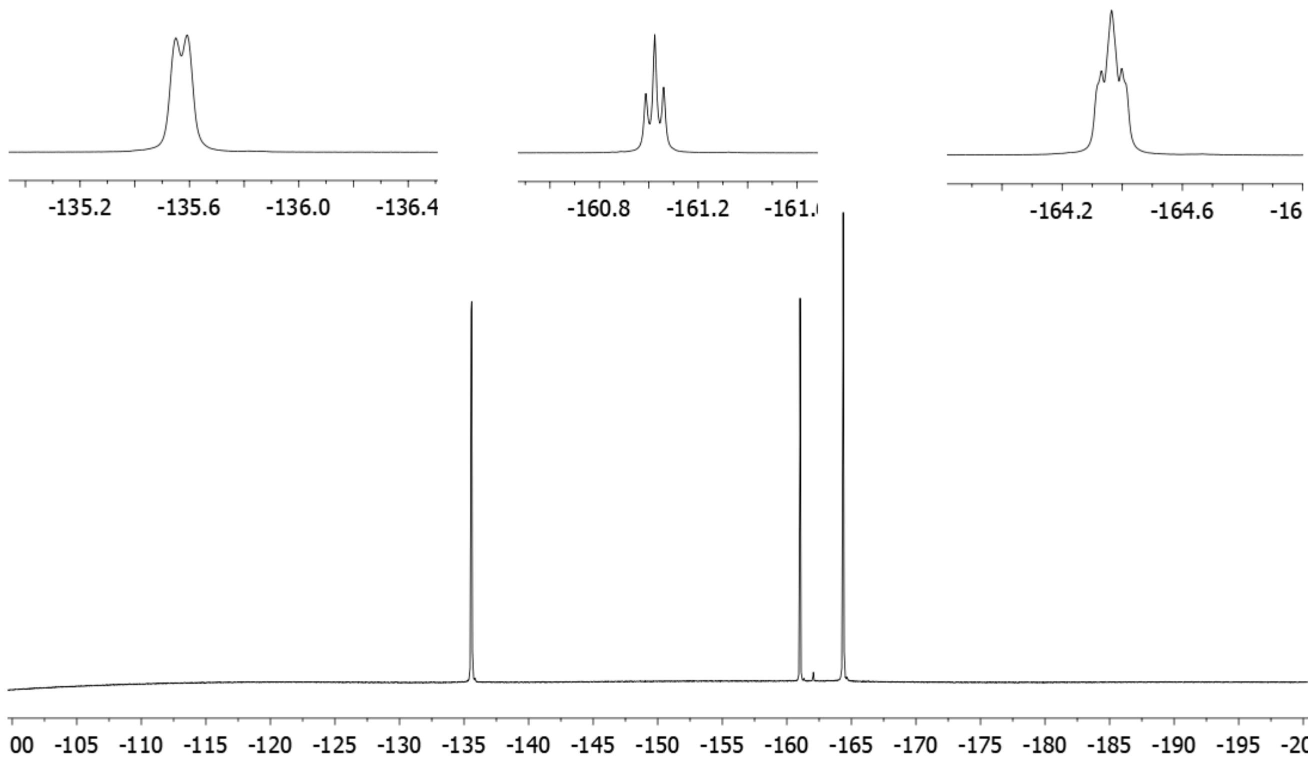

Figure S3. ^{19}F NMR (564 MHz, C_6D_6, 299 K) spectrum of compound **8**.

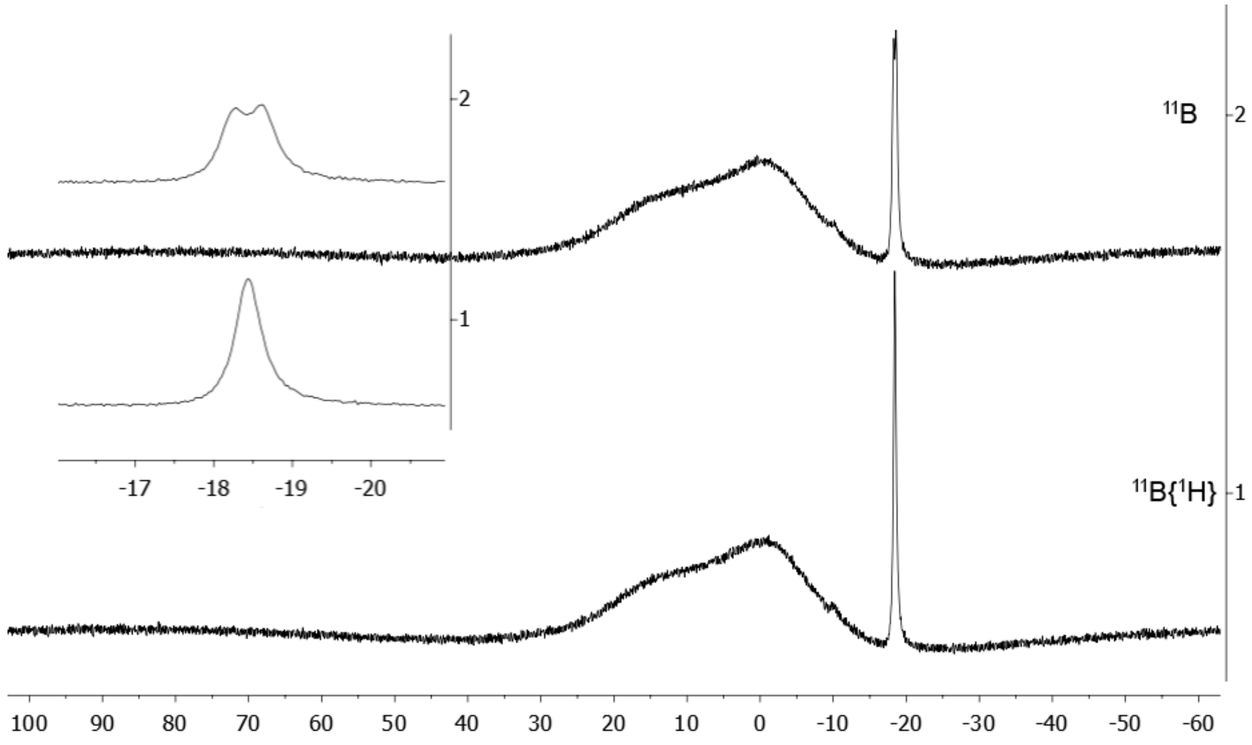

Figure S4. (1) **^{11}B{^{1}H}** and (2) **^{11}B NMR** (192 MHz, C_6D_6, 299 K) spectra of compound **8**.

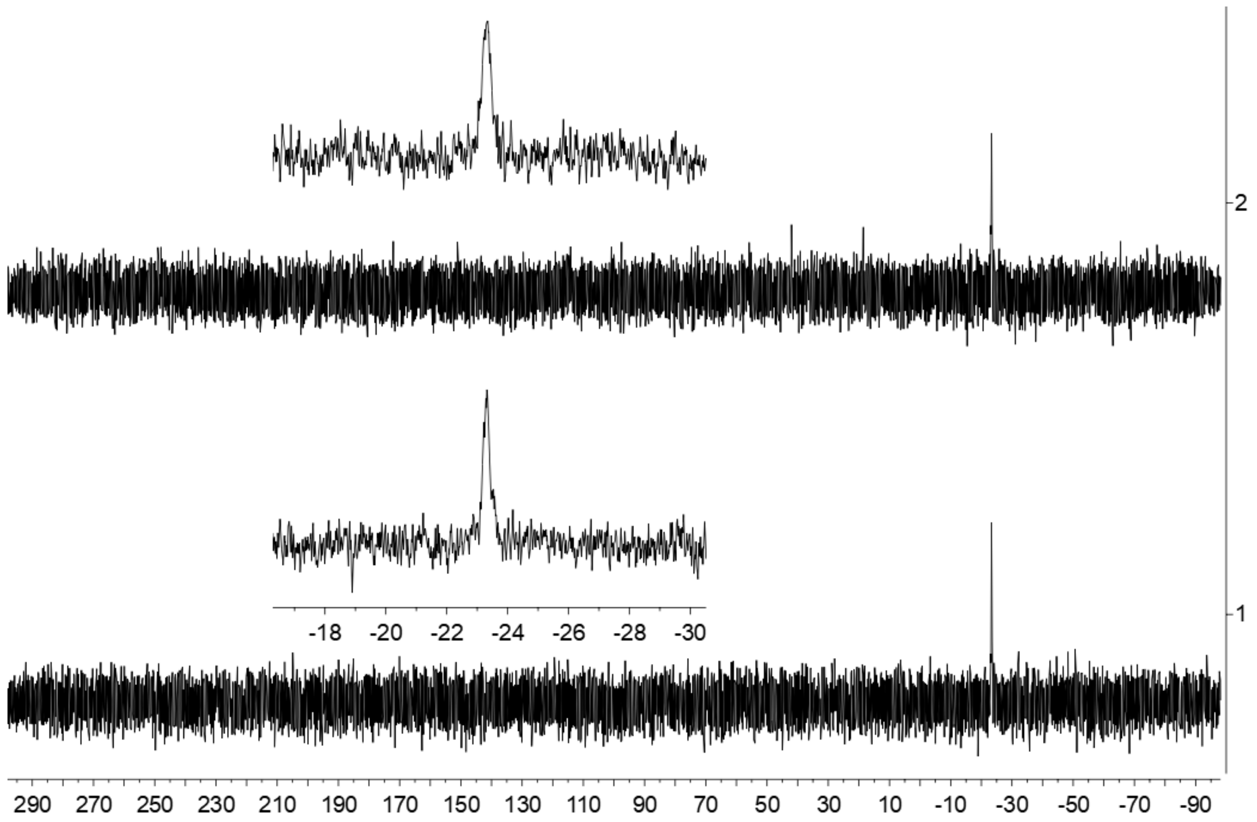

Figure S5. (1) **^{31}P{^{1}H}** and (2) **^{31}P NMR** (243 MHz, C_6D_6, 299 K) spectra of compound **8**.

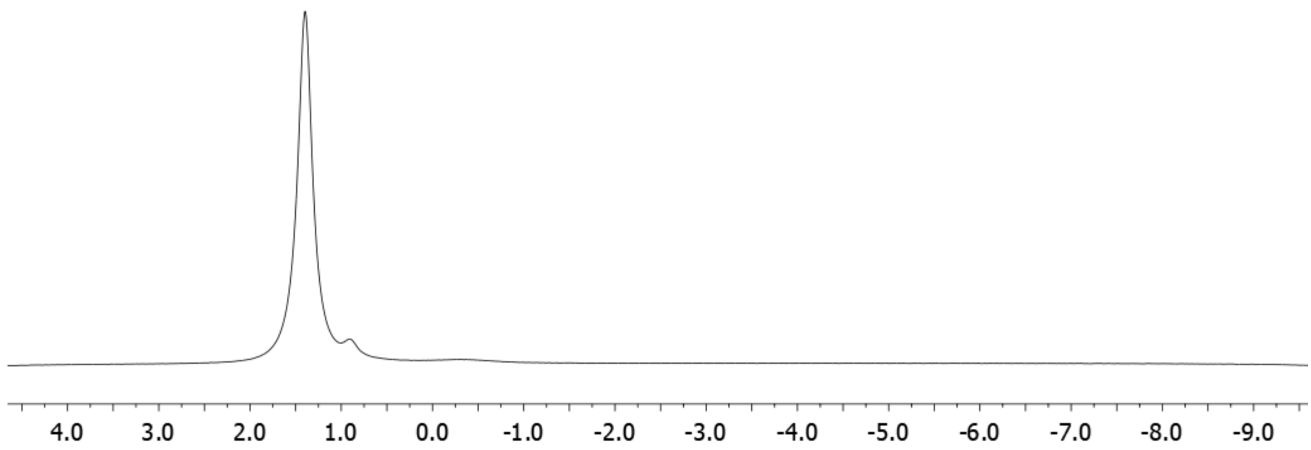

Figure S6. **^{7}Li NMR** (194 MHz, C_6D_6, 299 K) spectra of compound **8**.

Crystals suitable for the X-ray crystal structure analysis were obtained from a solution of compound **8** in *n*-pentane at -30 °C.

X-ray crystal structure analysis of compound 8 (erk9302): A colorless prism-like specimen of $C_{43}H_{52}BF_{10}LiNP$, approximate dimensions 0.040 mm x 0.080 mm x 0.120 mm, was used for the X-ray crystallographic analysis. The X-ray intensity data were measured. The integration of the data using a triclinic unit cell yielded a total of 11018 reflections to a maximum θ angle of 25.00° (0.84 Å resolution), of which 7372 were independent (average redundancy 1.495, completeness = 98.3%, R_{int} = 3.73%, R_{sig} = 5.26%) and 5599

(75.95%) were greater than 2σ(F^2). The final cell constants of a = 12.0597(2) Å, b = 12.1028(3) Å, c = 16.2479(5) Å, α = 98.0450(10)°, β = 99.3150(10)°, γ = 111.182(2)°, volume = 2130.75(10) $Å^3$, are based upon the refinement of the XYZ-centroids of reflections above 20 σ(I). Data were corrected for absorption effects using the multi-scan method (SADABS). The calculated minimum and maximum transmission coefficients (based on crystal size) are 0.9840 and 0.9940. The structure was solved and refined using the Bruker SHELXTL Software Package, using the space group *P*-1, with Z = 2 for the formula unit, $C_{43}H_{52}BF_{10}LiNP$. The final anisotropic full-matrix least-squares refinement on F^2 with 574 variables converged at R1 = 7.30%, for the observed data and wR2 = 15.24% for all data. The goodness-of-fit was 1.094. The largest peak in the final difference electron density synthesis was 0.292 $e^-/Å^3$ and the largest hole was -0.336 $e^-/Å^3$ with an RMS deviation of 0.052 $e^-/Å^3$. On the basis of the final model, the calculated density was 1.281 g/cm^3 and F(000), 860 e^-. The hydrogen at B1 atom was refined freely. CCDC number: 1960302.

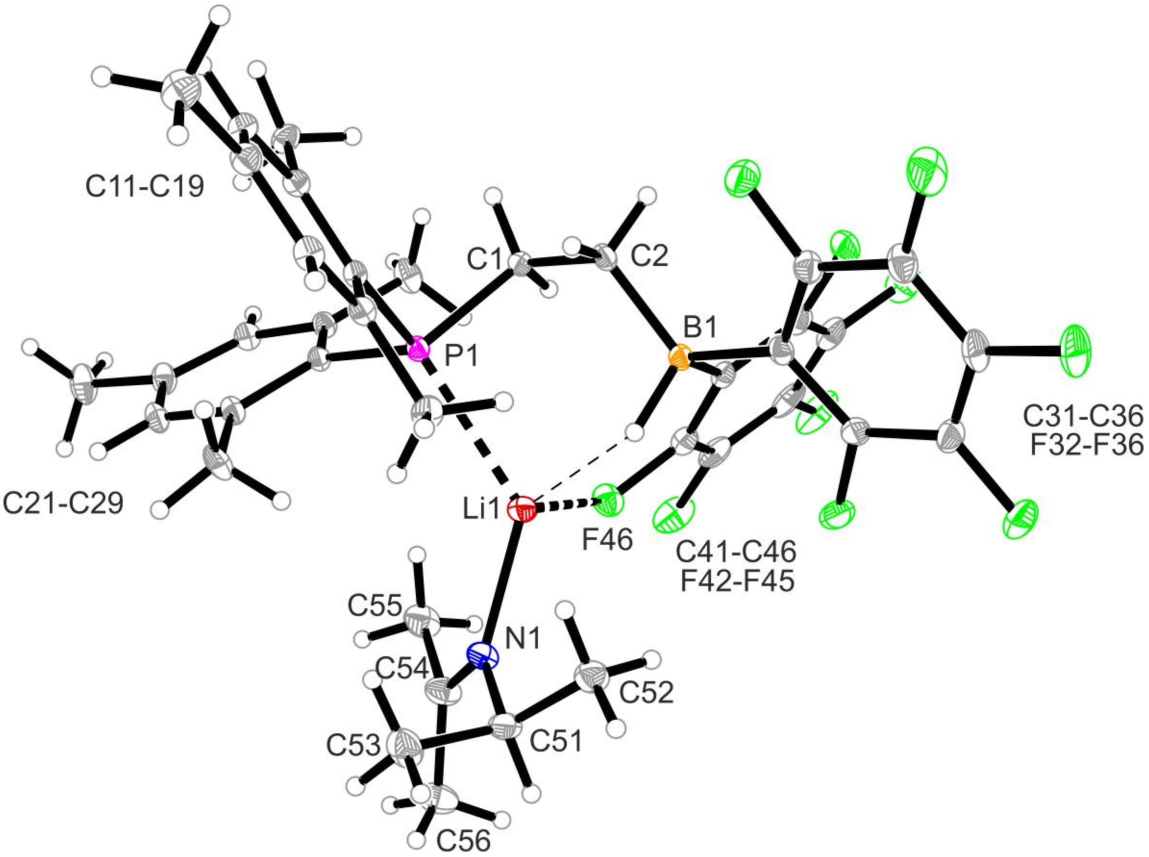

Figure S7. Crystal structure of compound **8** (thermal ellipsoids: 15% probability).

Preparation of compound 9

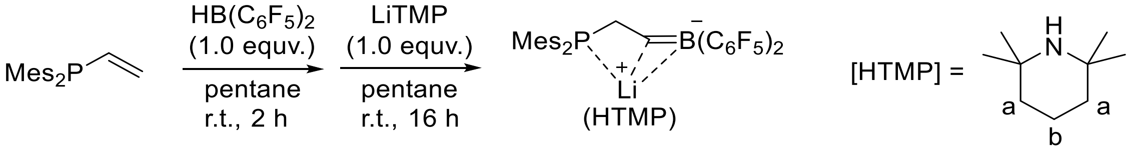

Scheme S2

A mixture of dimesitylvinylphosphine (592.8 mg, 2.0 mmol) and bis(pentafluorophenyl)borane (691.9 mg, 2.0 mmol) in *n*-pentane (20 mL) was stirred for 2 hours at room temperature. Then, LiTMP (294.1 mg, 2.0 mmol) was added to the reaction mixture. After stirring the obtained suspension for 16 hours at room temperature, the supernatant was removed by decantation. The obtained residue was washed with *n*-pentane (3× 10 mL) and dried *in vacuo* to give compound **9** as a white powder (1.231 g, 1.56 mmol, 78%).

Melting point: 142 °C

Elemental Analysis calcd for $C_{41}H_{44}BF_{10}PNLi$ (789.52 g/mol): C, 62.37; H, 5.62; N, 1.77. Found: C, 62.07; H, 5.43; N, 1.70.

^{1}H NMR (600 MHz, THF-d_8, 299 K): δ = 6.55 (m, 4H, *m*-Mes), 4.21 (q, $^3J_{HH}$ ~ $^3J_{PH}$ = 8.9 Hz, 1H, BCH), 3.36 (d, $^3J_{HH}$ = 8.9 Hz, 2H, PCH_2), 2.21 (s, 12H, *o*-Me^{Mes}), 2.12 (s, 6H, *p*-Me^{Mes}), 1.62 (m, 2H, $^bCH_2^{TMP}$), 1.29 (m, 4H, $^aCH_2^{TMP}$), 1.06 (s, 12H, Me^{TMP}), n.o. (NH^{TMP}).

^{13}C{^{1}H} NMR (151 MHz, THF-d_8, 299 K): δ = 142.8 (d, $^2J_{PC}$ =12.5 Hz, *o*-Mes), 138.1 (d, $^1J_{PC}$ = 32.2 Hz, *i*-Mes), 136.0 (*p*-Mes), 129.7 (*m*-Mes), 106.7 (br, BCH), 50.1 (NC^{TMP}), 39.2 ($^aCH_2^{TMP}$), 33.5 (d, $^1J_{PC}$ = 16.0 Hz, PCH_2), 32.2 (Me^{TMP}), 23.6 (d, $^3J_{PC}$ =11.3 Hz, *o*-Me^{Mes}), 20.9 (*p*-Me^{Mes}), 19.3 ($^bCH_2^{TMP}$), [C_6F_5 not listed].

^{11}B NMR (192 MHz, THF-d_8, 299 K): δ = 18.6 ($\nu_{1/2}$ ~ 350 Hz).

^{31}P NMR (243 MHz, THF-d_8, 299 K): δ = -20.6 ($\nu_{1/2}$ ~ 30 Hz).

^{19}F NMR (564 MHz, THF-d_8, 299 K): δ = [-131.2, -132.6](each m, each 2F, *o*-C_6F_5), [-167.7, -168.8](each m, each 1F, *p*-C_6F_5), [-168.9, -169.4](each m, each 2F, *p*-C_6F_5).

^{7}Li NMR (194 MHz, THF-d_8, 299 K): δ = -0.4 ($\nu_{1/2}$ ~ 10 Hz).

^{7}Li NMR (194 MHz, CD_2Cl_2, 299 K): δ = 1.4 ($\nu_{1/2}$ ~ 30 Hz).

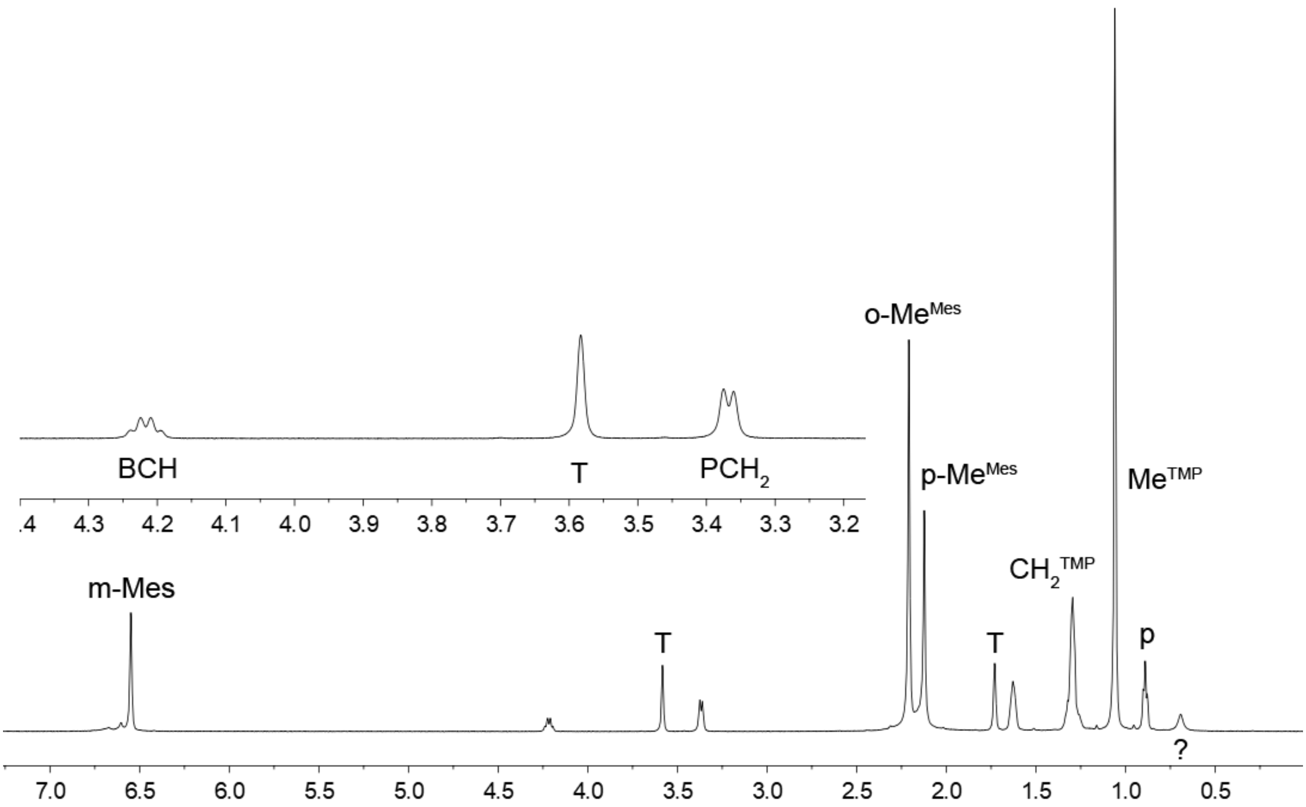

Figure S8. **^{1}H NMR** (600 MHz, THF-d$_8$, 299 K) spectrum of compound **9**.[T: THF-d$_7$, p: pentane, ?: tentatively assigned as NH]

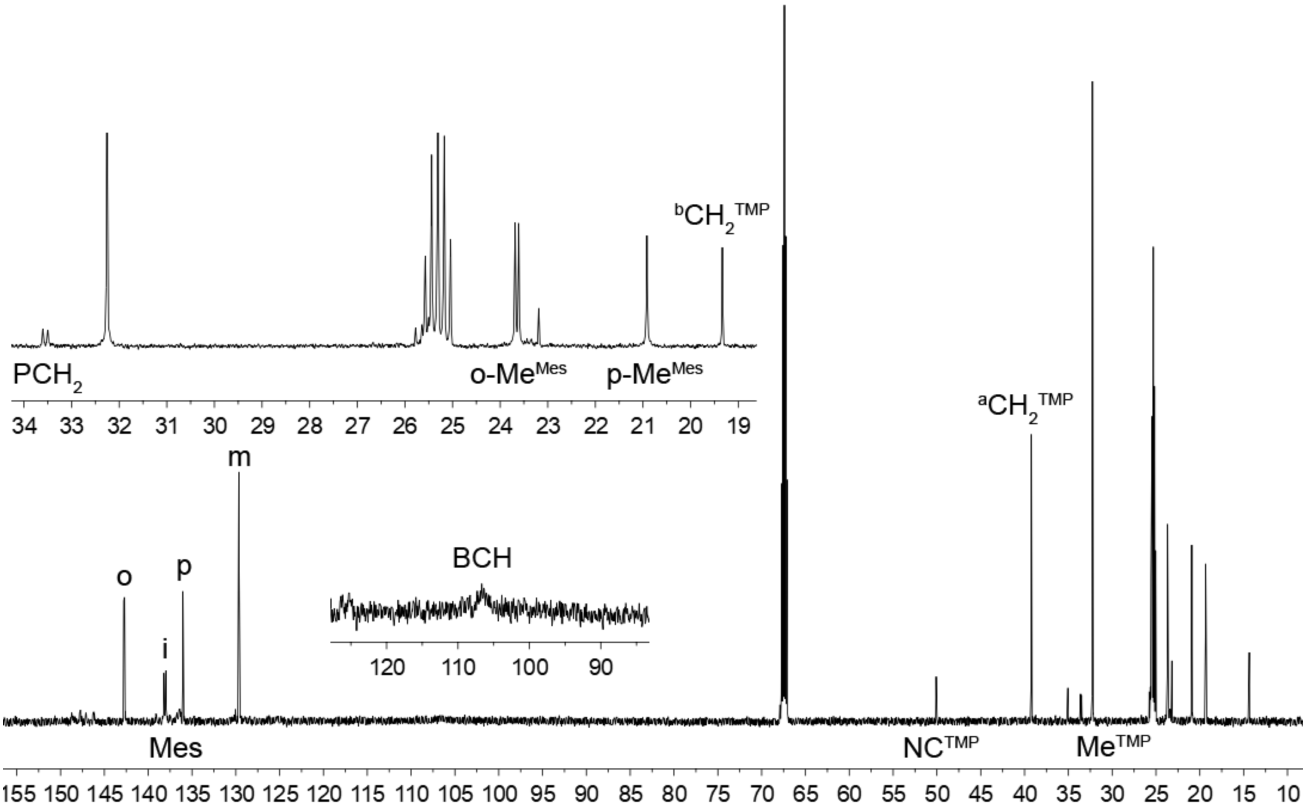

Figure S9. **^{13}C{^{1}H} NMR** (151 MHz, THF-d$_8$, 299 K) spectrum of compound **9**.

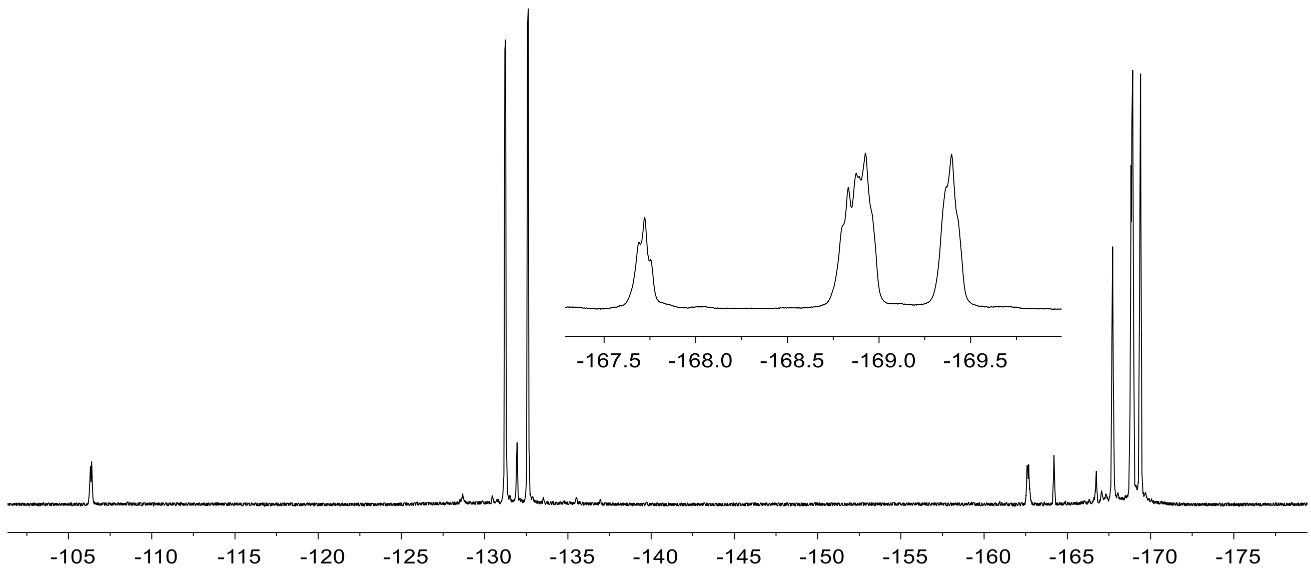

Figure S10. ^{19}**F NMR** (564 MHz, THF-d$_8$, 299 K) spectrum of compound **9**.

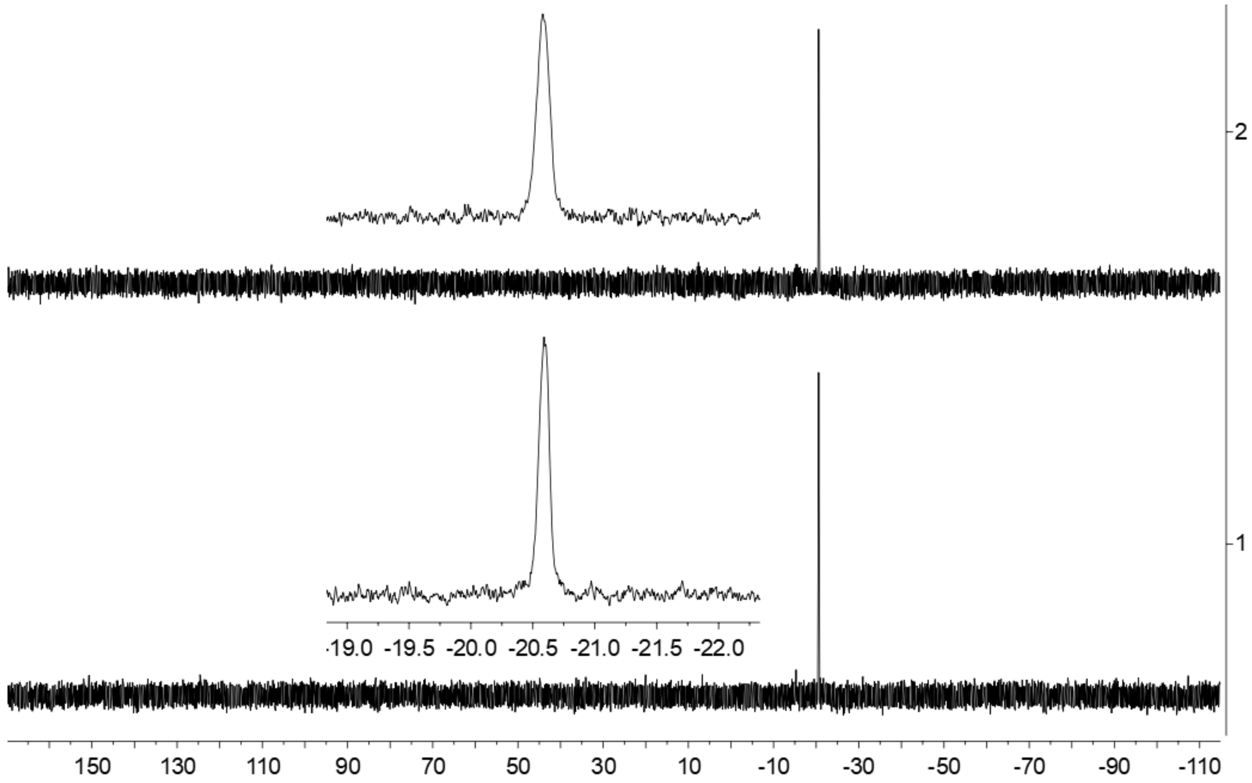

Figure S11. (1) ^{31}**P{^{1}H}** and (2) ^{31}**P NMR** (243 MHz, THF-d$_8$, 299 K) spectra of compound **9**.

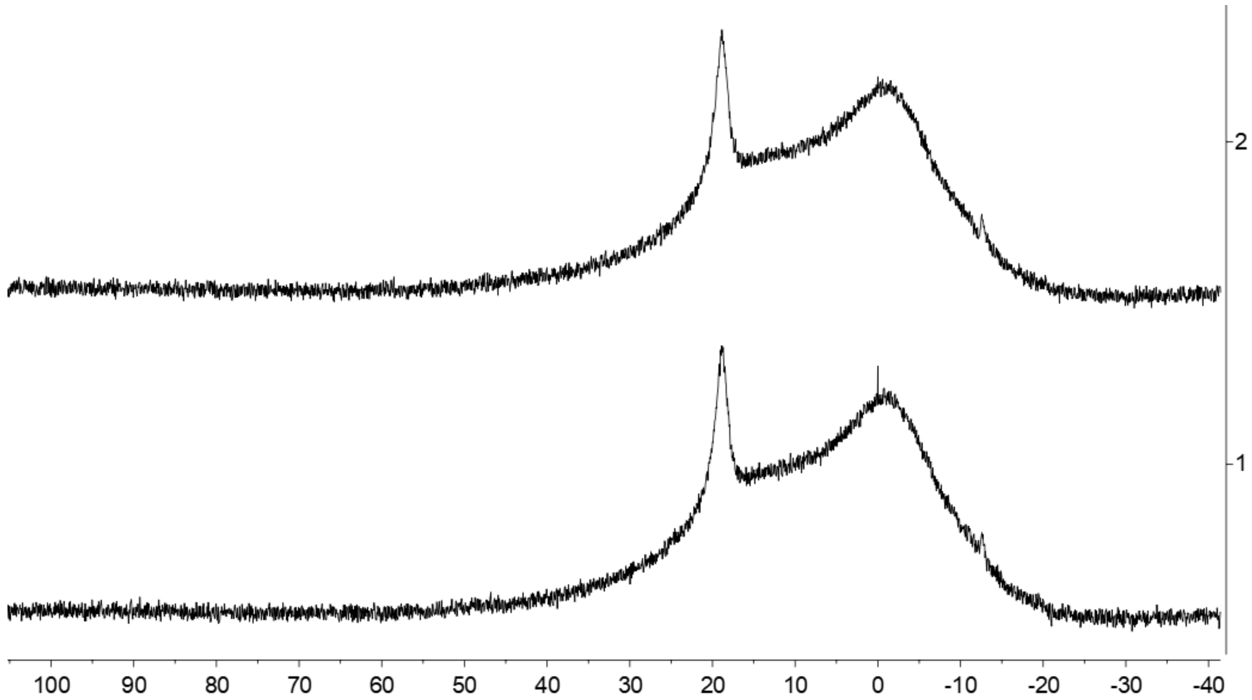

Figure S12. (1) ^{11}**B{**^{1}**H}** and (2) ^{11}**B NMR** (192 MHz, THF-d$_8$, 299 K) spectra of compound **9**.

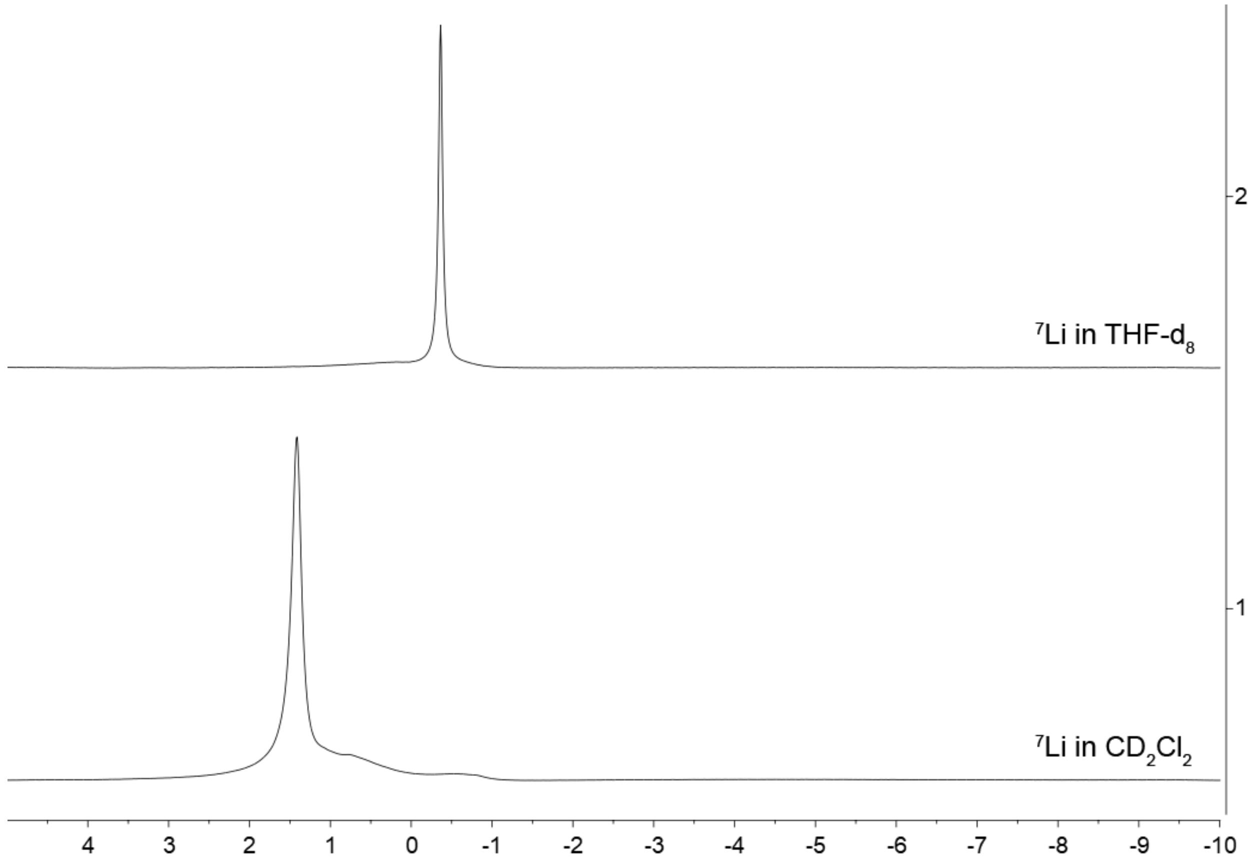

Figure S13. ^{7}**Li NMR** (194 MHz, 299 K) spectrum of compound **9** in (1) CD_2Cl_2 and (2) in THF-d$_8$.

Crystals suitable for the X-ray crystal structure analysis were obtained from a saturated solution of compound **9** in toluene at -30 °C.

X-ray crystal structure analysis of compound 9 (erk8412): A colorless prism-like specimen of $C_{48}H_{52}BF_{10}LiNP$, approximate dimensions 0.140 mm x 0.180 mm x 0.230 mm, was used for the X-ray crystallographic analysis. The X-ray intensity data were measured. The integration of the data using a monoclinic unit cell yielded a total of 14760 reflections to a maximum θ angle of 25.00° (0.84 Å resolution), of which 7964 were independent (average redundancy 1.853, completeness = 99.3%, R_{int} = 2.86%, R_{sig} = 3.39%) and 6527 (81.96%) were greater than 2σ(F^2). The final cell constants of a = 12.4065(2) Å, b = 20.2711(4) Å, c = 18.1486(4) Å, β = 95.6760(10)°, volume = 4541.88(15) $Å^3$, are based upon the refinement of the XYZ-centroids of reflections above 20 σ(I). Data were corrected for absorption effects using the multi-scan method (SADABS). The calculated minimum and maximum transmission coefficients (based on crystal size) are 0.9700 and 0.9810. The structure was solved and refined using the Bruker SHELXTL Software Package, using the space group $P2_1/n$, with Z = 4 for the formula unit, $C_{48}H_{52}BF_{10}LiNP$. The final anisotropic full-matrix least-squares refinement on F^2 with 734 variables converged at R1 = 5.05%, for the observed data and wR2 = 13.77% for all data. The goodness-of-fit was 1.043. The largest peak in the final difference electron density synthesis was 0.243 $e^-/Å^3$ and the largest hole was -0.207 $e^-/Å^3$ with an RMS deviation of 0.040 $e^-/Å^3$. On the basis of the final model, the calculated density was 1.289 g/cm^3 and F(000), 1840 e^-. The hydrogen at N1 atom was refined freely. CCDC number: 1960303.

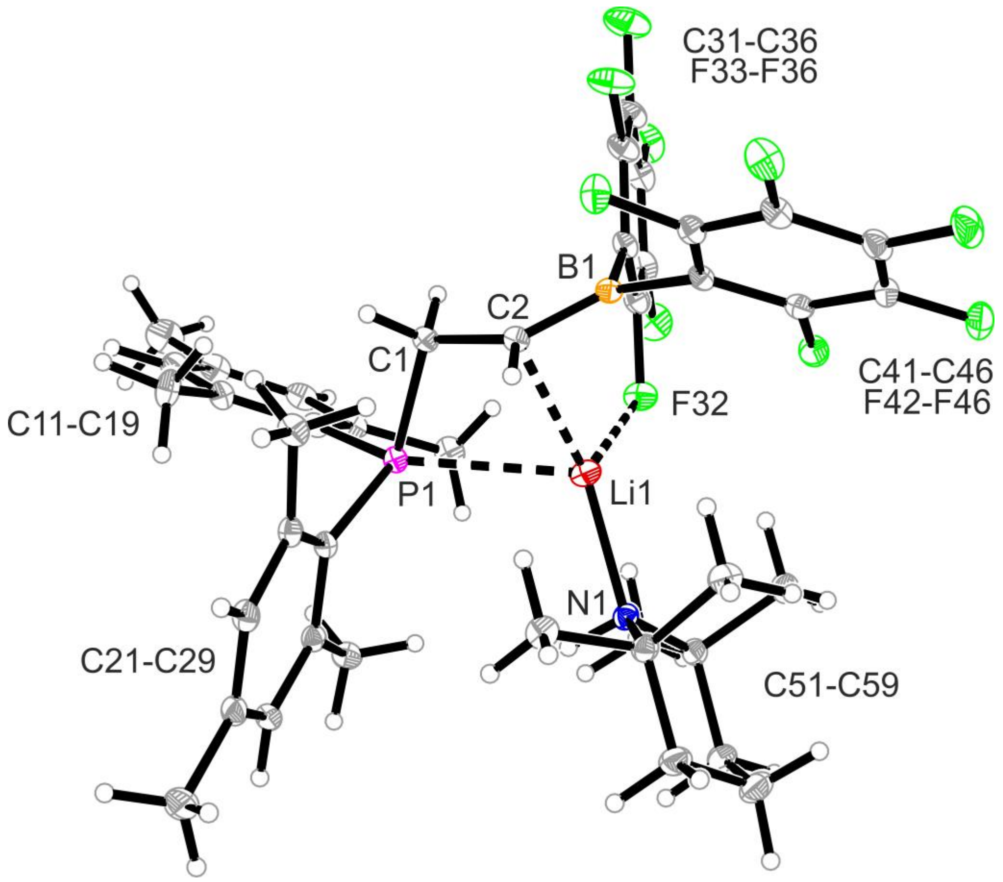

Figure S14. Crystal structure of compound **9** (thermal ellipsoids: 15% probability).

Preparation of compound 10

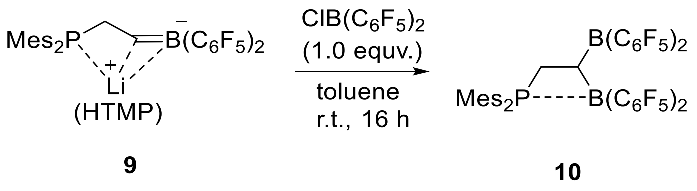

Scheme S3

A mixture of compound **9** (500 mg, 0.63 mmol) and chlorobis(pentafluorophenyl)borane (240 mg, 0.63 mmol) in toluene (10 mL) was stirred for 16 hours at room temperature. Then, the reaction mixture was filtered and all volatiles were removed *in vacuo*. Then, pentane (5 mL) was added and the obtained solid was collected, washed with *n*-pentane (3× 10 mL) and dried *in vacuo* to give compound **10** as a yellow powder (325 mg, 0.33 mmol, 52%).

Melting point: 152 °C

Elemental Analysis calcd for $C_{44}H_{25}B_2F_{20}P$ (986.25 g/mol): C, 53.59; H, 2.56. Found: C, 53.79; H, 2.68.

^{1}H NMR (600 MHz, CD_2Cl_2, 299 K): δ = 6.83 (m, 4H, *m*-Mes), 4.30 (br t, $^3J_{HH}$ = 10.3 Hz, 1H, BCH), 3.55 (t, $^3J_{HH}$ ~ $^2J_{PH}$ =10.3 Hz, 2H, PCH_2), 2.30 (br, 12H, *o*-Me^{Mes}), 2.24 (s, 6H, *p*-Me^{Mes}).

^{1}H NMR (600 MHz, CD_2Cl_2, 233 K): δ = [6.93 (br)/6.46 (br), 6.89 (br d, $^4J_{PH}$ =4.7 Hz)/6.81 (br)](each 1H, *m*-Mes), 4.22 (m, 1H, BCH), [3.59, 3.39](each m, each 1H, PCH_2), [2.78/1.54 (each 3H), 2.27 (6H)](each s, *o*-Me^{Mes}), [2.19, 2.15](each s, each 3H, *p*-Me^{Mes}).

^{13}C{^{1}H} NMR (151 MHz, CD_2Cl_2, 299 K): δ = 142.9 (br d, $^2J_{PC}$ = 8.1 Hz, *o*-Mes), 142.2 (d, $^4J_{PC}$ = 2.7 Hz, *p*-Mes), 131.2 (d, $^3J_{PC}$ = 7.4 Hz, *m*-Mes), 123.9 (br, *i*-Mes), 38.1 (br, BCH), 29.2 (d, $^1J_{PC}$ = 40.3 Hz, PCH_2) 22.9 (d, $^3J_{PC}$ = 6.4 Hz, *o*-Me^{Mes}), 20.8 (*p*-Me^{Mes}), [C_6F_5 not listed].

^{19}F NMR (564 MHz, CD_2Cl_2, 299 K): δ -130.0 (br, 2F, *o*-C_6F_5), -154.8 (br, 1F, *p*-C_6F_5), -163.4 (br, 2F, *m*-C_6F_5), [$\Delta^{19}F_{m,p}$ = 8.6].

^{19}F NMR (564 MHz, CD_2Cl_2, 203 K): δ = [-120.1 (1F), -124.0 (1F), -126.5 (1F), -128.7 (3F), -130.8 (1F), -132.9 (1F)] (each br m, *o*-C_6F_5), [-148.2 (br, 2F), -154.8 (m, 1F), -156.0 (m, 1F)](*p*-C_6F_5), [-159.6 (br, 4F), -160.5 (m, 1F), -162.7 (m, 1F), -163.1 (m, 1F), -163.2 (m, 1F)](*m*-C_6F_5).

^{10}B NMR (64 MHz, CD_2Cl_2, 299 K): δ = 40.5 ($\nu_{1/2}$ ~ 1500 Hz), -2.2 ($\nu_{1/2}$ ~ 1600 Hz).

^{31}P NMR (243 MHz, CD_2Cl_2, 299 K): δ = 16.3 ($\nu_{1/2}$ ~ 35 Hz).

^{31}P NMR (243 MHz, CD_2Cl_2, 203 K): δ = 15.4 ($\nu_{1/2}$ ~ 45 Hz).

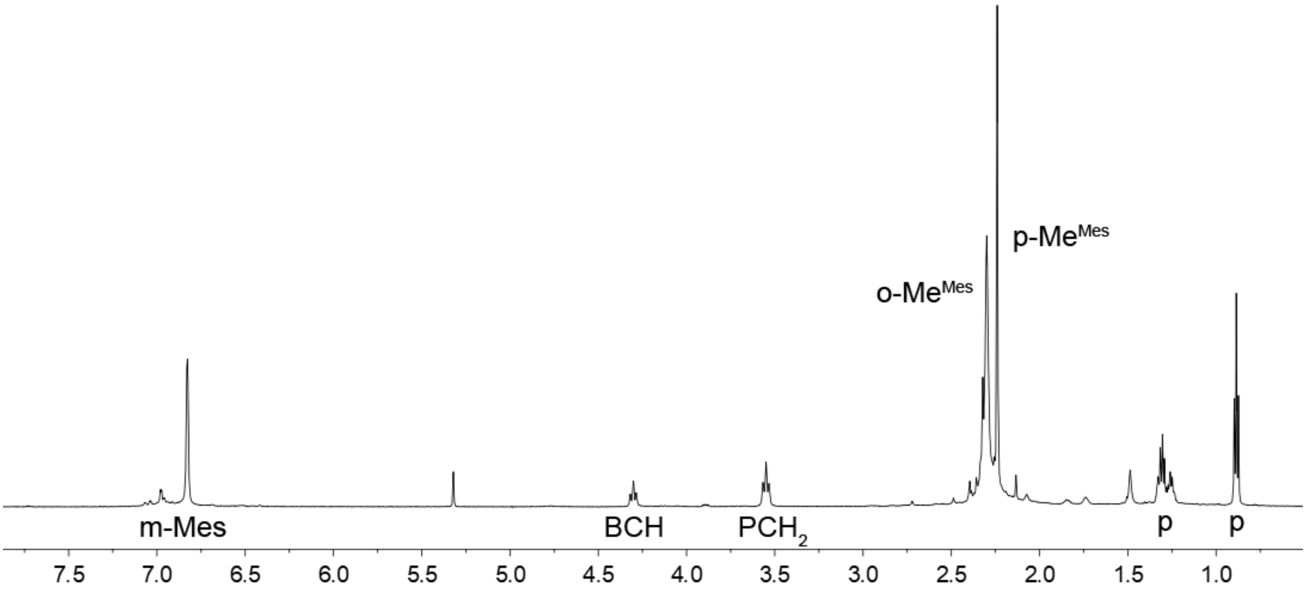

Figure S15. **^{1}H NMR** (600 MHz, CD_2Cl_2, 299 K) spectrum of compound **10** [admixed with pentane (p)].

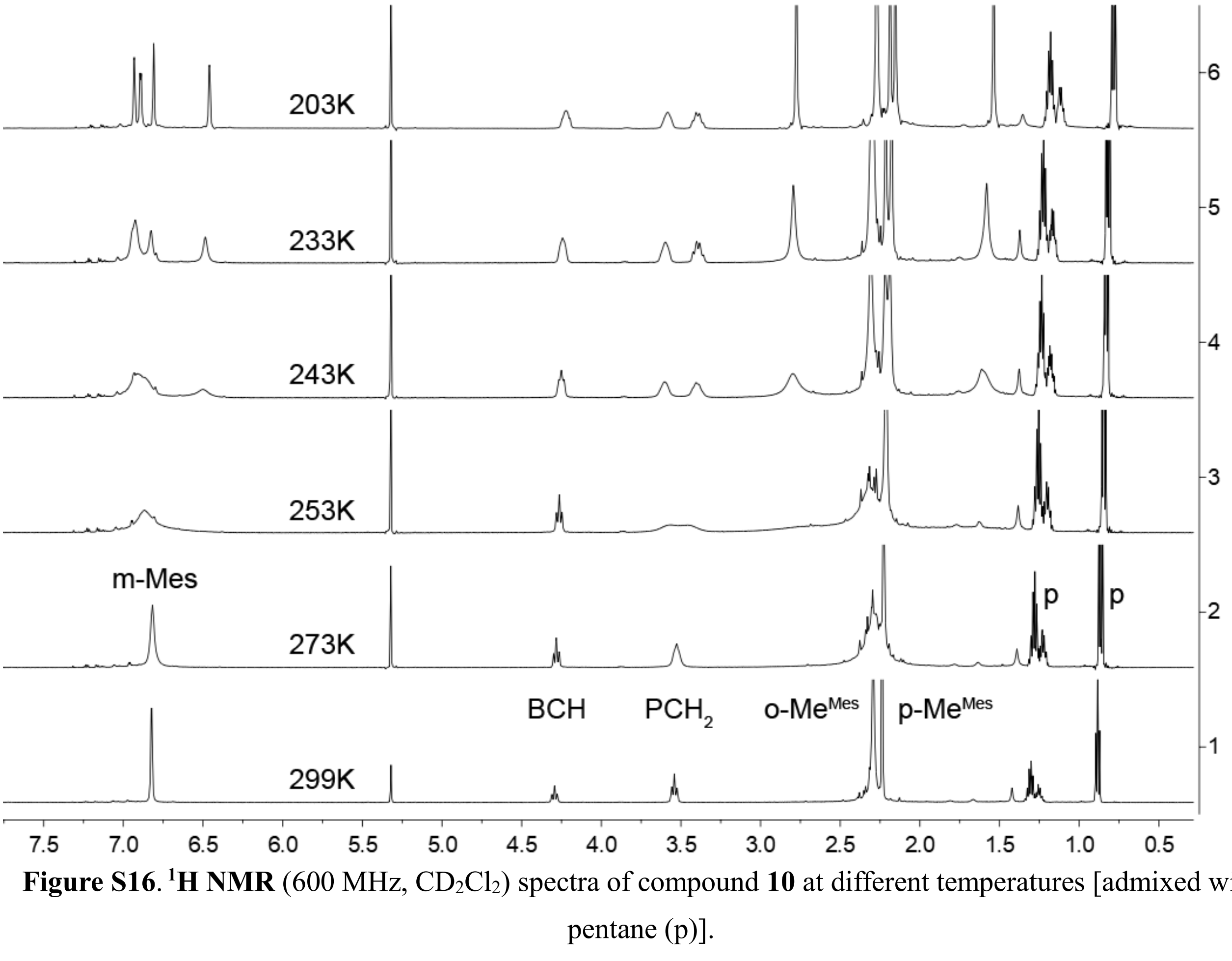

Figure S16. **^{1}H NMR** (600 MHz, CD_2Cl_2) spectra of compound **10** at different temperatures [admixed with pentane (p)].

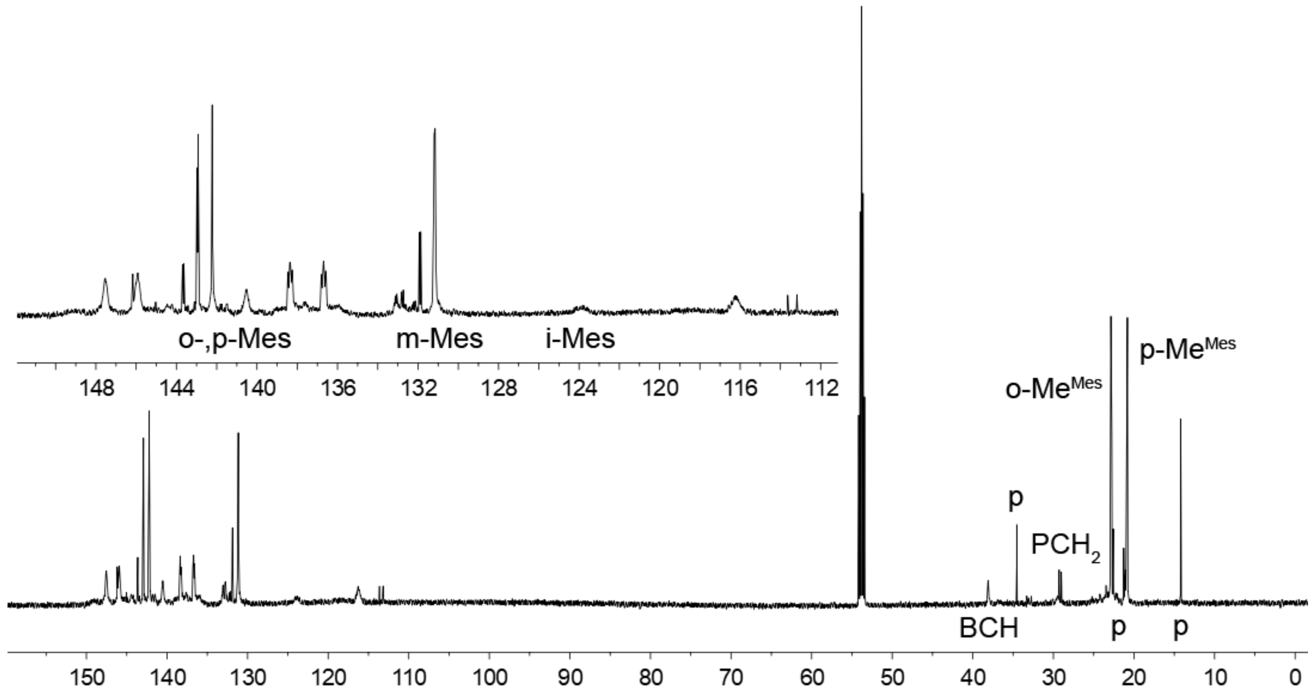

Figure S17. **$^{13}C\{^1H\}$ NMR** (151 MHz, CD_2Cl_2, 299 K) spectrum of compound **10** [admixed with pentane (p)].

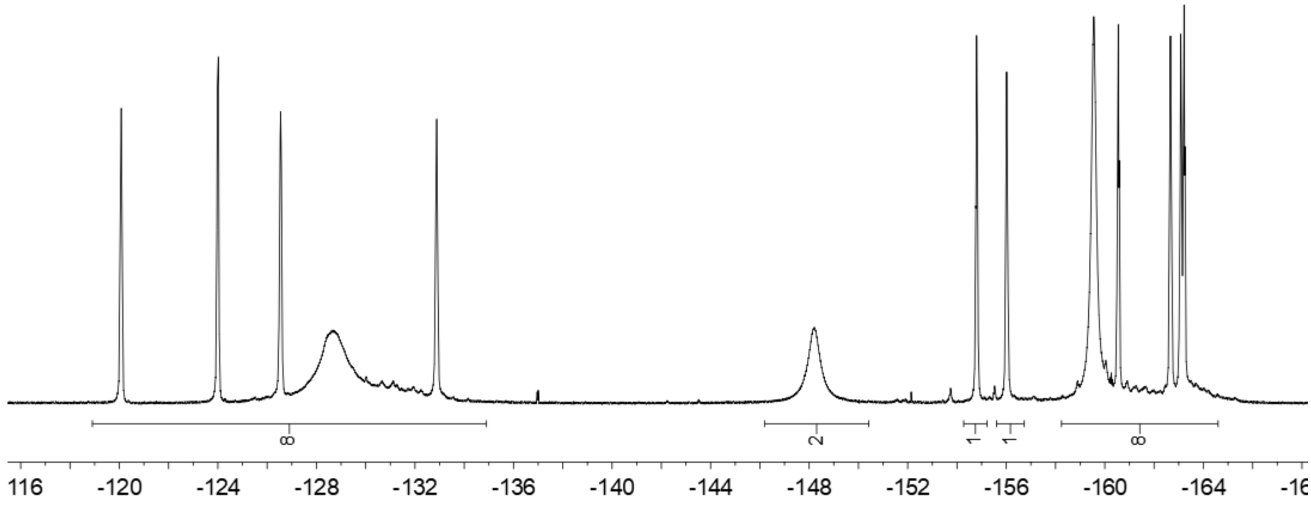

Figure S18. **^{19}F NMR** (564 MHz, CD_2Cl_2, 203 K) spectrum of compound **10**.

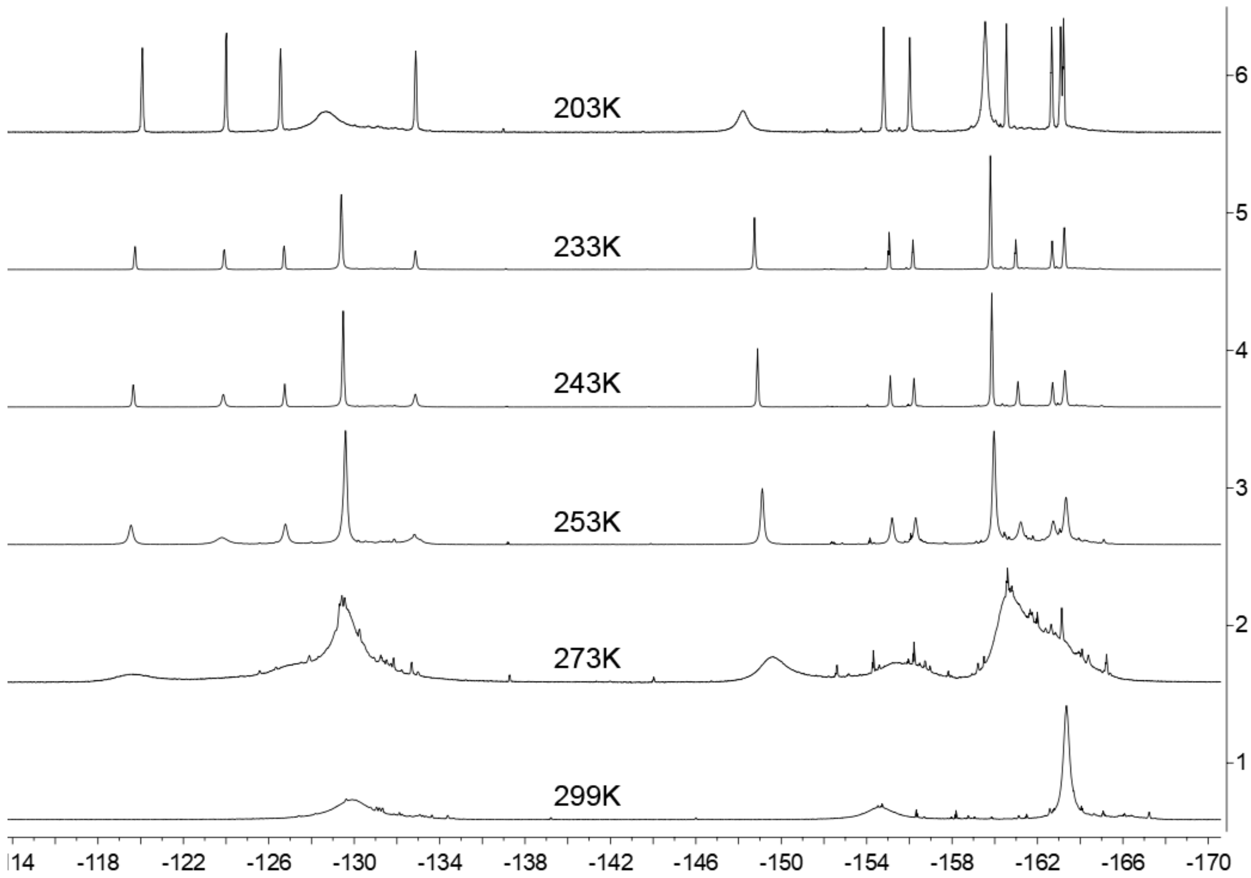

Figure S19. **^{19}F NMR** (564 MHz, CD_2Cl_2) spectra of compound **10** at different temperatures.

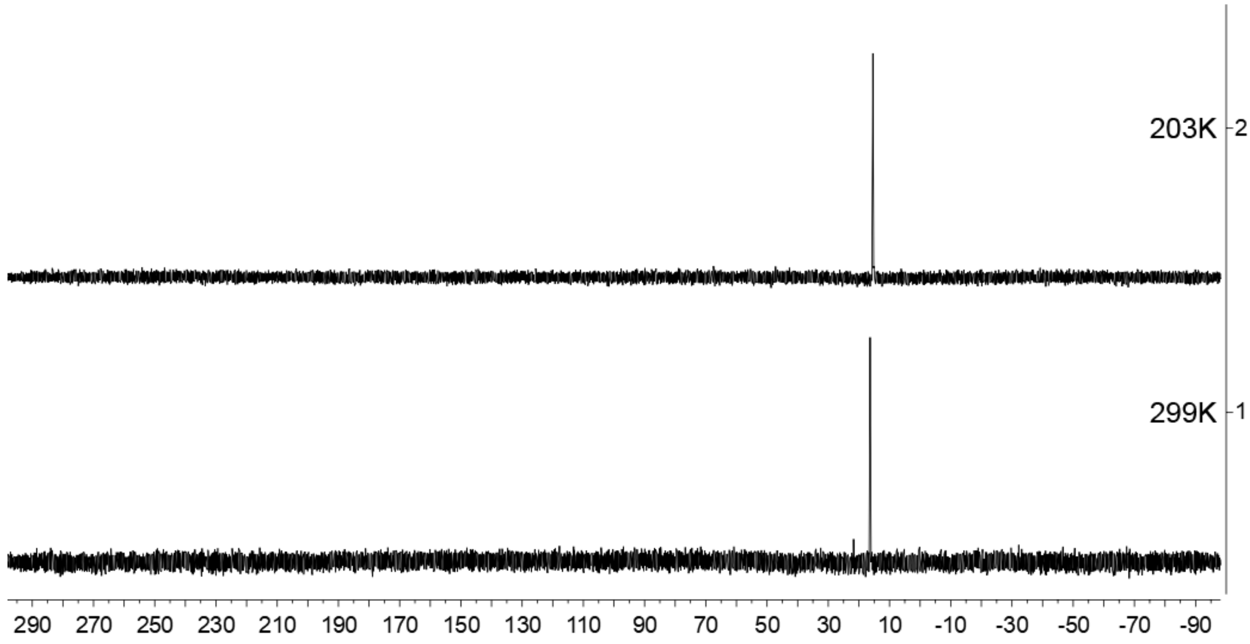

Figure S20. ^{31}P NMR (243 MHz, CD_2Cl_2) spectra of compound **10** at (1) 299K and (2) 203K.

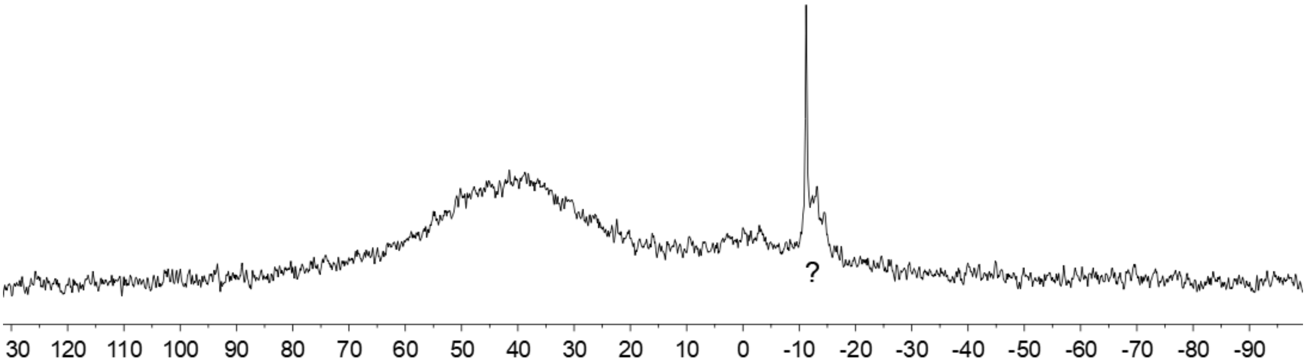

Figure S21. **^{10}B NMR** (64 MHz, CD_2Cl_2, 299 K) spectra of compound **10** (? unknown compound).

Preparation of compound 11

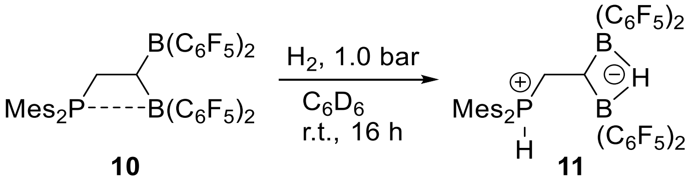

10 **11**

Scheme S4

Compound **10** (98 mg, 0.10 mmol) was dissolved in C_6D_6 (2 mL) in a Schlenk tube. Then, this solution was stirred for 16 hours at room temperature in an H_2 atmosphere (1.0 bar). All volatiles were removed *in vacuo* and pentane (3 mL) was added. After stirring the obtained suspension for 15 minutes, the supernatant was removed and the remaining solid was dried *in vacuo* to give compound **11** as a pale yellow powder (70 mg, 0.07 mmol, 71%).

Melting point: 156 °C

Elemental Analysis calcd for $C_{44}H_{27}B_2F_{20}P$ (988.26 g/mol): C, 53.48; H, 2.75. Found: C, 53.18; H, 2.52.

^{1}H NMR (600 MHz, CD_2Cl_2, 299 K): δ = 7.58 (dt, $^1J_{PH}$ ~ 478.6 Hz, $^3J_{HH}$ = 5.9 Hz 1H, PH), 7.07 (d, $^4J_{PH}$ = 4.2 Hz, 4H, *m*-Mes), 5.45 (br m, 1H, BH)[t], 2.84 (ddm, $^3J_{HH}$ = 10.2 Hz, 1H, PCH_2), 2.36 (s, 12H, *o*-Me^{Mes}), 2.35 (s, 6H, *p*-Me^{Mes}), 1.80 (m, 1H, BCH), [t tentatively assigned].

^{13}C{^{1}H} NMR (151 MHz, CD_2Cl_2, 299 K): δ = 146.2 (d, $^4J_{PC}$ = 2.9 Hz, *p*-Mes), 143.5 (d, $^2J_{PC}$ = 10.1 Hz, *o*-Mes), 132.2 (d, $^3J_{PC}$ = 11.2 Hz, *m*-Mes), 113.2 (d, $^1J_{PC}$ = 76.4 Hz, *i*-Mes), 27.8 (d, $^1J_{PC}$ = 42.9 Hz, PCH_2), 22.3 (d, $^3J_{PC}$ = 6.9 Hz, *o*-Me^{Mes}), 21.3 (*p*-Me^{Mes}), 7.6 (br, BCH), [C_6F_5 not listed].

^{19}F NMR (564 MHz, CD_2Cl_2, 299 K): δ [-129.6, -132.2](each br, each 2F, *o*-C_6F_5), [-157.9, -158.8] (each br m, each 1F, *p*-C_6F_5), [-163.7, -165.2](each br m, each 2F, *m*-C_6F_5).

^{11}B NMR (192 MHz, CD_2Cl_2, 299 K): δ = -18.1 ($\nu_{1/2}$ ~ 450 Hz).

^{11}B{^{1}H} NMR (192 MHz, CD_2Cl_2, 299 K): δ = -18.1 ($\nu_{1/2}$ ~ 450 Hz).

^{31}P NMR (243 MHz, CD_2Cl_2, 299 K): δ = -3.7 (dm, $^1J_{PH}$ ~ 480 Hz).

^{31}P{^{1}H} NMR (243 MHz, CD_2Cl_2, 299 K): δ = -3.7 ($\nu_{1/2}$ ~ 20 Hz).

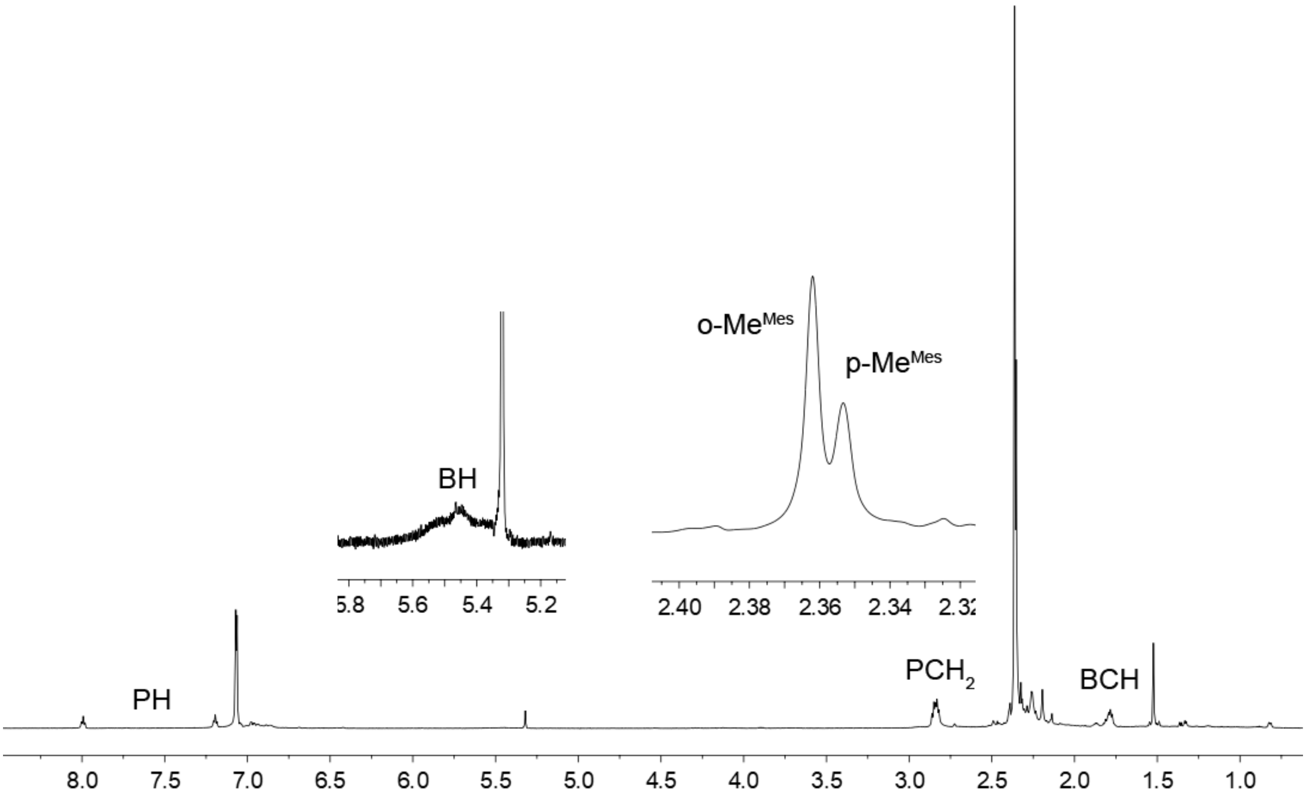

Figure S22. **^{1}H NMR** (600 MHz, CD_2Cl_2, 299 K) spectrum of compound **11**.

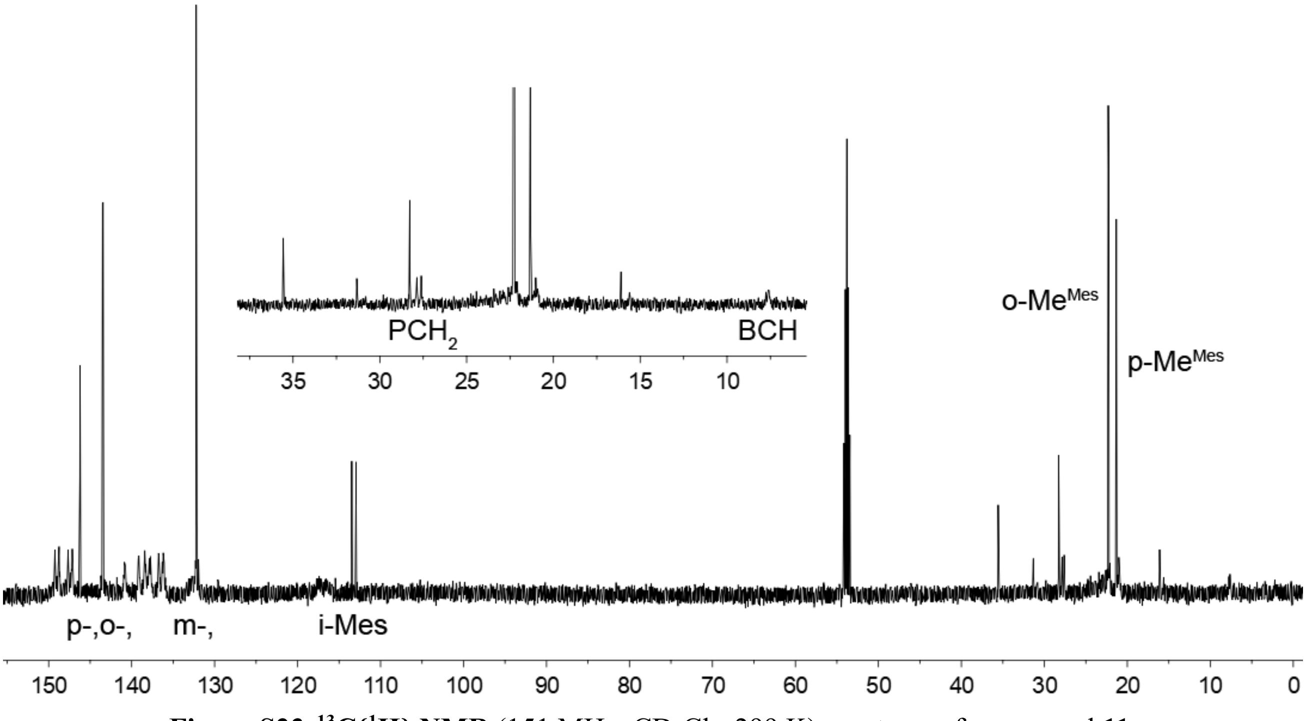

Figure S23. **$^{13}C\{^1H\}$ NMR** (151 MHz, CD_2Cl_2, 299 K) spectrum of compound **11**.

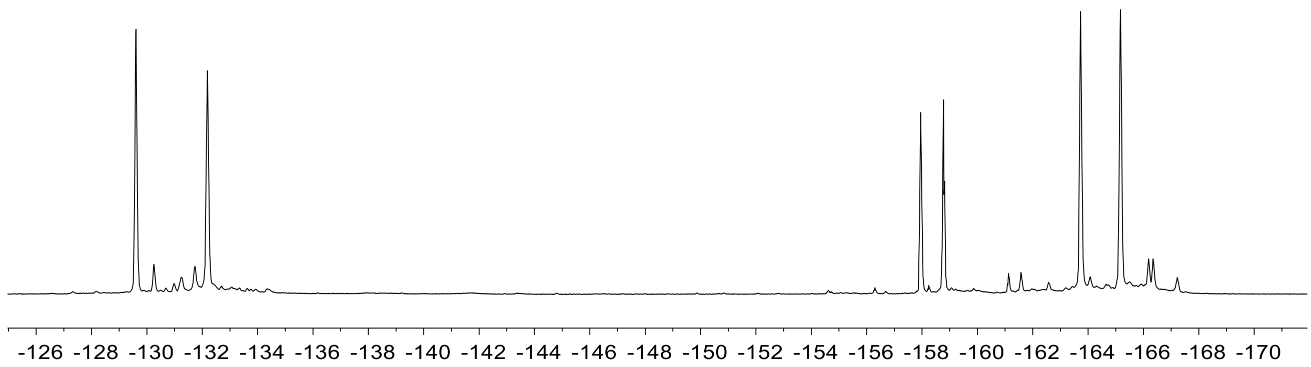

Figure S24. **^{19}F NMR** (564 MHz, CD_2Cl_2, 299 K) spectrum of compound **11**.

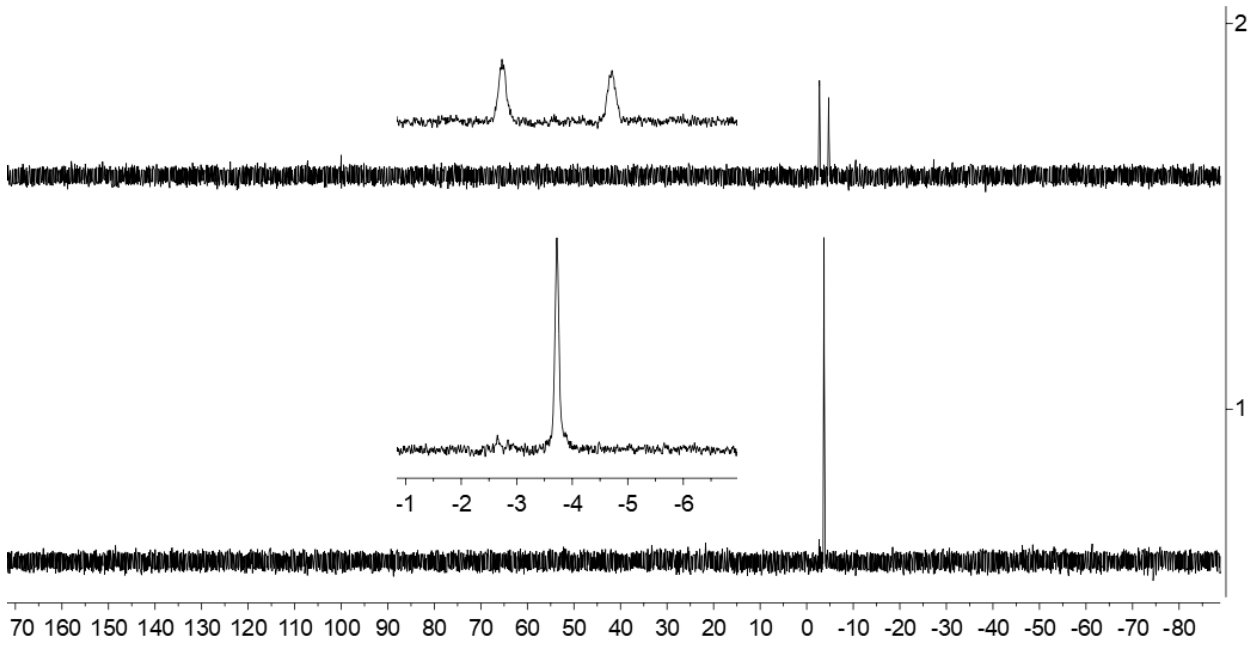

Figure S25. (1) **^{31}P{^{1}H}** and (2) **^{31}P NMR** (243 MHz, CD_2Cl_2, 299 K) spectra of compound **11**.

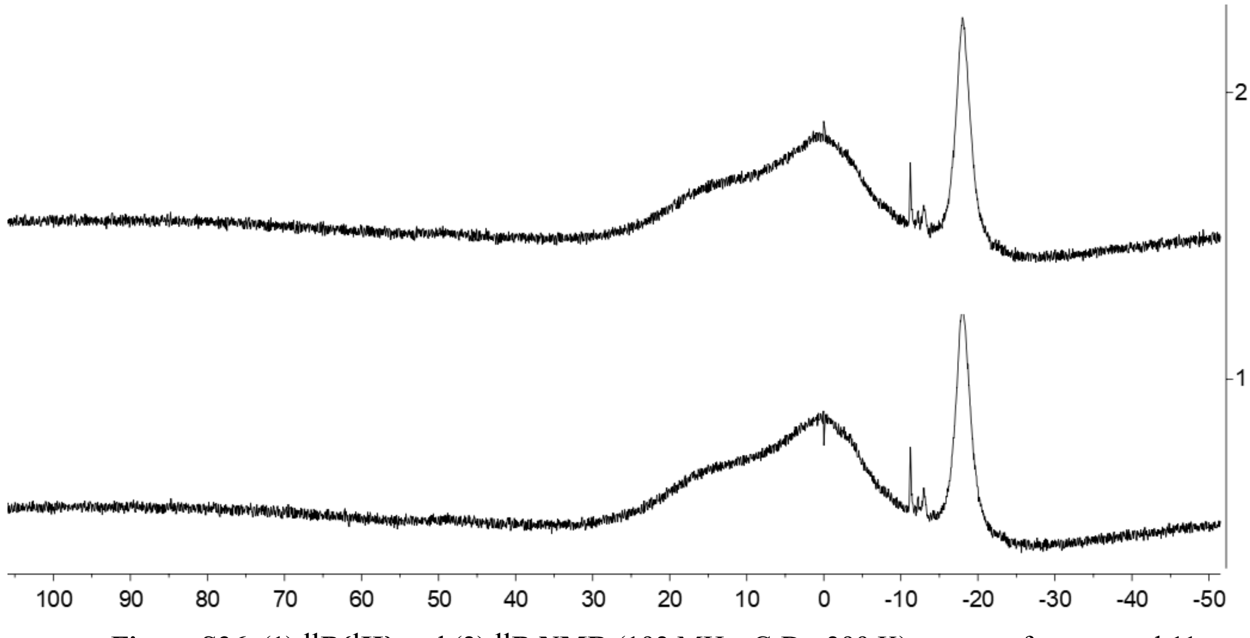

Figure S26. (1) **^{11}B{^{1}H}** and (2) **^{11}B NMR** (192 MHz, C_6D_6, 299 K) spectra of compound **11**.

Crystals suitable for X-ray crystal structure analysis were obtained from slow diffusion of pentane into saturated solution of compound **11** in CH_2Cl_2 at -30 °C.

X-ray crystal structure analysis of compound 11 (erk8821): A colorless prism-like specimen of $C_{44}H_{27}B_2F_{20}P$, approximate dimensions 0.087 mm x 0.141 mm x 0.274 mm, was used for the X-ray crystallographic analysis. The X-ray intensity data were measured. A total of 1653 frames were collected. The total exposure time was 28.65 hours. The frames were integrated with the Bruker SAINT software package using a wide-frame algorithm. The integration of the data using a triclinic unit cell yielded a total of 24980 reflections to a maximum θ angle of 68.40° (0.83 Å resolution), of which 8162 were independent (average redundancy 3.061, completeness = 97.2%, R_{int} = 4.15%, R_{sig} = 4.36%) and 6547 (80.21%) were greater than 2σ(F^2). The final cell constants of a = 10.4373(3) Å, b = 11.5018(3) Å, c = 20.0520(6) Å, α = 101.416(2)°, β = 99.845(2)°, γ = 98.080(2)°, volume = 2286.61(11) $Å^3$, are based upon the refinement of the XYZ-centroids of 9943 reflections above 20 σ(I) with 8.247° < 2θ < 136.6°. Data were corrected for absorption effects using the multi-scan method (SADABS). The ratio of minimum to maximum apparent transmission was 0.769. The calculated minimum and maximum transmission coefficients (based on crystal size) are 0.6740 and 0.8760. The structure was solved and refined using the Bruker SHELXTL Software Package, using the space group *P*-1, with Z = 2 for the formula unit, $C_{44}H_{27}B_2F_{20}P$. The final anisotropic full-matrix least-squares refinement on F^2 with 618 variables converged at R1 = 4.65%, for the observed data and wR2 = 11.57% for all data. The goodness-of-fit was 1.022. The largest peak in the final difference electron density synthesis was 1.196 $e^-/Å^3$ and the largest hole was -0.421 $e^-/Å^3$ with an RMS deviation of 0.053 $e^-/Å^3$. On the basis of the final model, the calculated density was 1.435 g/cm^3 and F(000), 992 e^-. The hydrogen at P1 atom and the hydrogen between the B1 and B2 atoms were refined freely. CCDC number: 1960304.

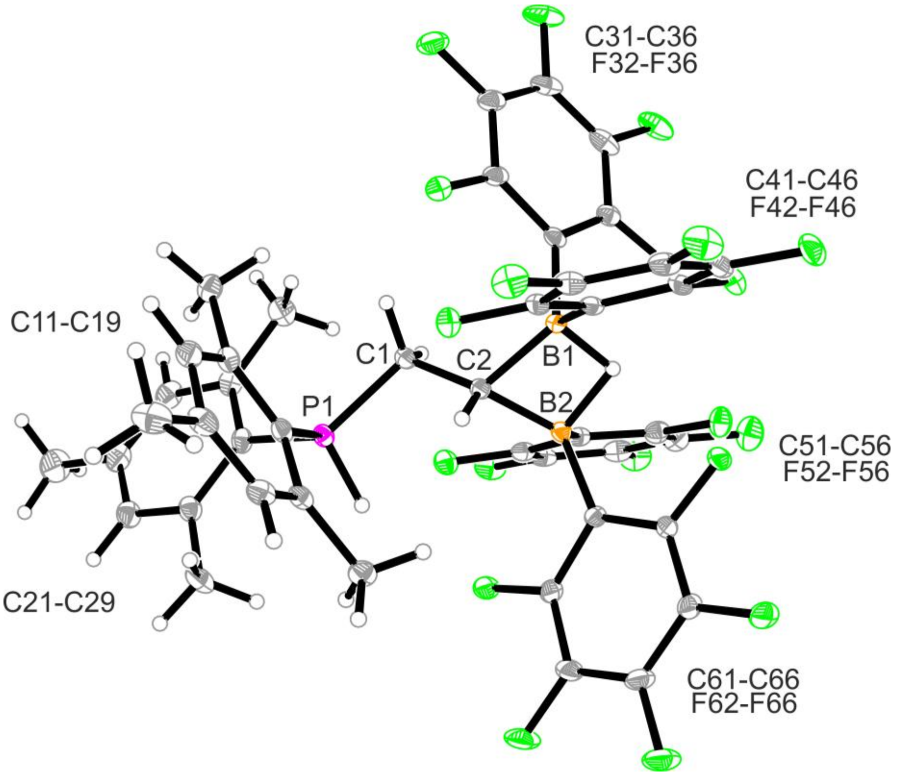

Figure S27. Crystal structure of compound **11** (thermal ellipsoids: 30% probability.

Preparation of compound 12

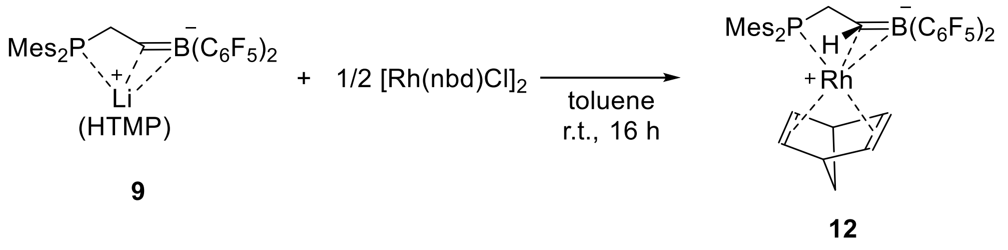

Scheme S5

1st Experiment: A mixture of compound **9** (947.4 g, 1.2 mmol) and bicyclo[2.2.1]hepta-2,5-diene-rhodium(I) chloride dimer (276.6 mg, 0.6 mmol) in toluene (12 mL) was stirred for 16 hours at room temperature. Then, all volatiles were removed *in vacuo*. The obtained residue was washed with *n*-pentane (3 × 10 mL) and dried *in vacuo* to give compound **12** as a yellow powder (710 mg, 0.85 mmol, 70%). The sample contains lithium chloride.

Decomposing point: 171 °C

HRMS (ESI, acetonitrile): *m/z* calc. for $[C_{39}H_{33}BF_{10}PRh+H]^+$ 837.1388; found 837.1399.

^{1}H NMR (600 MHz, THF-d$_8$, 299 K): δ = [6.97 (br), 6.78 (d, $^4J_{PH}$ = 2.3 Hz)](each 2H, *m*-Mes), [5.01, 4.08, 3.92, 3.86](s, each 1H, =CH^{nbd}), [4.18, 3.99](each m, each 1H, PCH$_2$), [3.84, 3.75] (s, each 1H, CH^{nbd}), 3.74 (m, 1H, BCH), [2.69 (br), 2.22 (s)](each 6H, *o*-Me^{Mes}), [2.28, 2.20](each s, each 3H, *p*-Me^{Mes}), 1.34 (s, 2H, CH$_2$^{nbd}).

^{13}C{^{1}H} NMR (151 MHz, THF-d$_8$, 299 K): δ = [142.4 (d, $^2J_{PC}$ = 8.7 Hz), 141.8 (d, $^2J_{PC}$ = 9.2 Hz)](*o*-Mes), [140.7 (d, $^4J_{PC}$ = 1.8 Hz), 140.4 (d, $^4J_{PC}$ = 1.7 Hz)](*p*-Mes), [131.5 (d, $^3J_{PC}$ = 7.5 Hz), 131.0 (d, $^3J_{PC}$ = 7.1 Hz)](*m*-Mes), [130.5 (d, $^1J_{PC}$ = 38.0 Hz), 127.8 (d, $^1J_{PC}$ = 23.5 Hz)](*i*-Mes), [81.7 (dd, $^1J_{RhC}$ = 11.1 Hz, $^2J_{PC}$ = 7.0 Hz), 78.7 (m), 67.2 (dm, $^1J_{RhC}$ = 8.9 Hz), 64.6 (d, $^1J_{RhC}$ = 7.8 Hz)](=CH^{nbd}), 66.7 (d, $^3J_{RhC}$ = 4.3 Hz, CH$_2$^{nbd}), 60.4 (br, BCH), [53.9, 53.1](CH^{nbd}), 43.3 (dd, $^1J_{PC}$ = 26.1, $^2J_{RhC}$ = 3.9 Hz, PCH$_2$), [23.6 (br), 23.4 (d, $^3J_{PC}$ = 8.2 Hz)](*o*-Me^{Mes}), [20.9, 20.8](*p*-Me^{Mes}), [C_6F_5 not listed].

^{11}B NMR (192 MHz, THF-d$_8$, 299 K): δ = 24.5 ($\nu_{1/2}$ ~ 700 Hz).

^{11}B NMR (192 MHz, CD_2Cl_2, 299 K): δ = 24.5 ($\nu_{1/2}$ ~ 600 Hz).

^{31}P NMR (243 MHz, THF-d$_8$, 299 K): δ = -88.3 (br d, $^1J_{RhP}$ ~ 120 Hz).

^{31}P NMR (243 MHz, CD_2Cl_2, 299 K): δ = -89.0 (br d, $^1J_{RhP}$ ~ 120 Hz).

^{19}F NMR (564 MHz, THF-d$_8$, 299 K): δ = [-129.3 (br), -130.6 (m)](each 2F, *o*-C_6F_5), [-158.9, -160.9](each t, $^3J_{FF}$ = 20.2 Hz, each 1F, *p*-C_6F_5), [-165.4, -165.7](each m, each 2F, *m*-C_6F_5).

^{19}F NMR (564 MHz, CD_2Cl_2, 299 K): δ = [-129.2 (br), -130.2 (m)](each 2F, *o*-C_6F_5), [-157.8, -159.8](each t, $^3J_{FF}$ = 20.2 Hz, each 1F, *p*-C_6F_5), [-164.6, -164.9](each m, each 2F, *m*-C_6F_5).

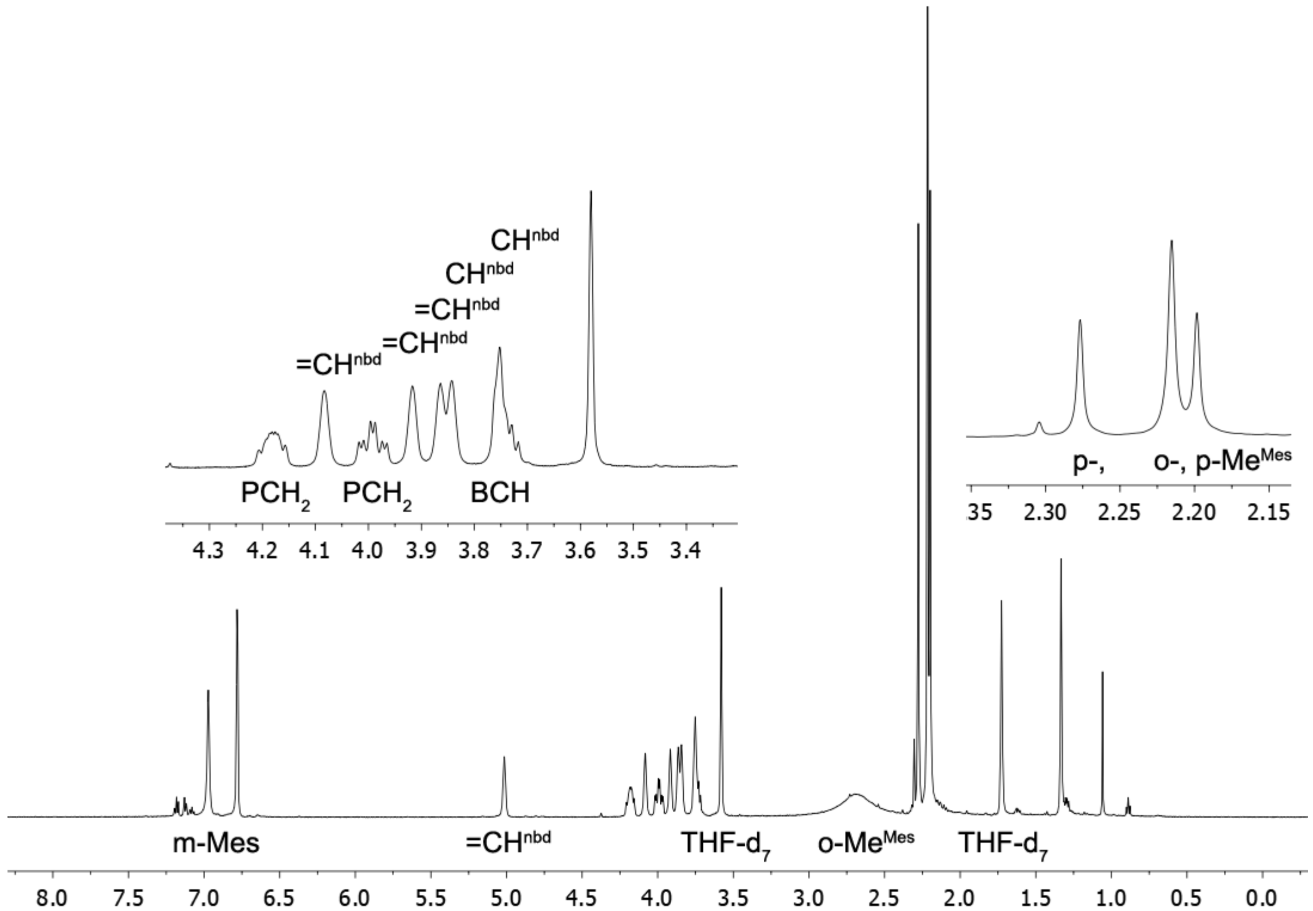

Figure S28. **^{1}H NMR** (600 MHz, THF-d_8, 299 K) spectrum of compound **12**.

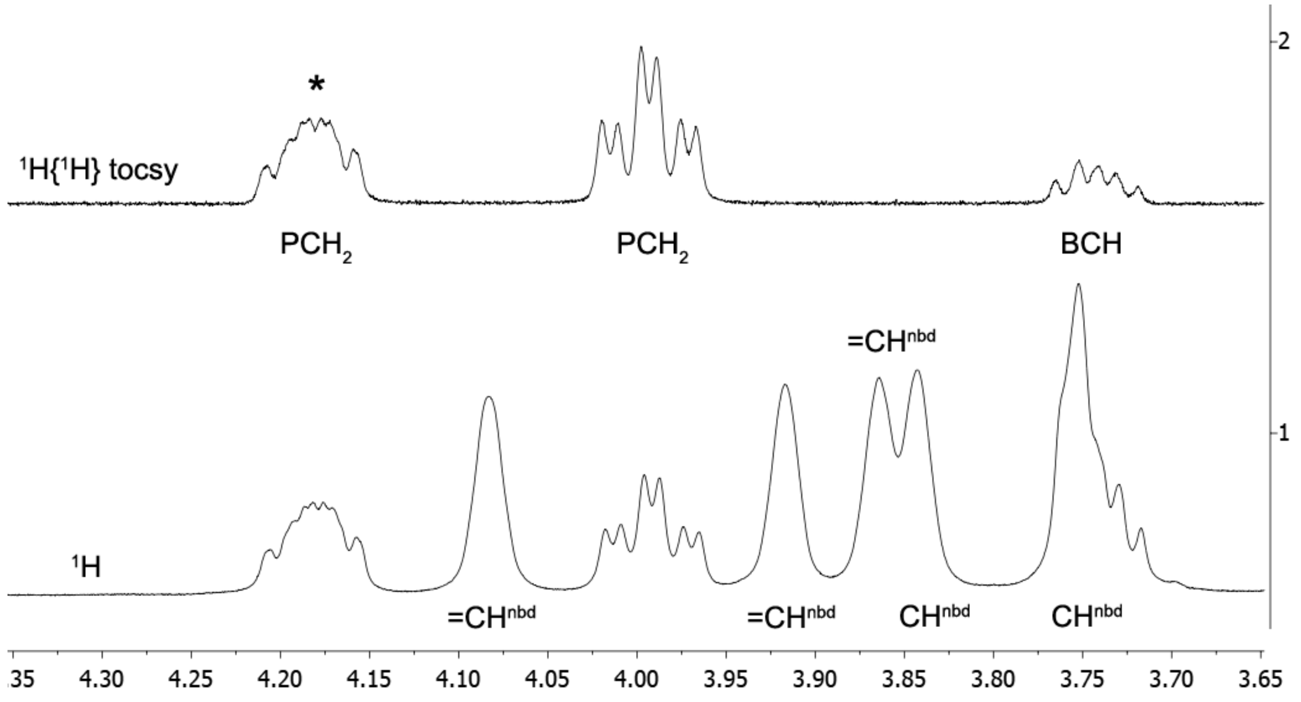

Figure S29. (1) **^{1}H NMR** (600 MHz, THF-d_8, 299 K) and (2) **^{1}H{^{1}H} TOCSY** [* irradiation point: δ $^1H_{irr}$ = 4.18 (PCH_2)] spectra of compound **12**.

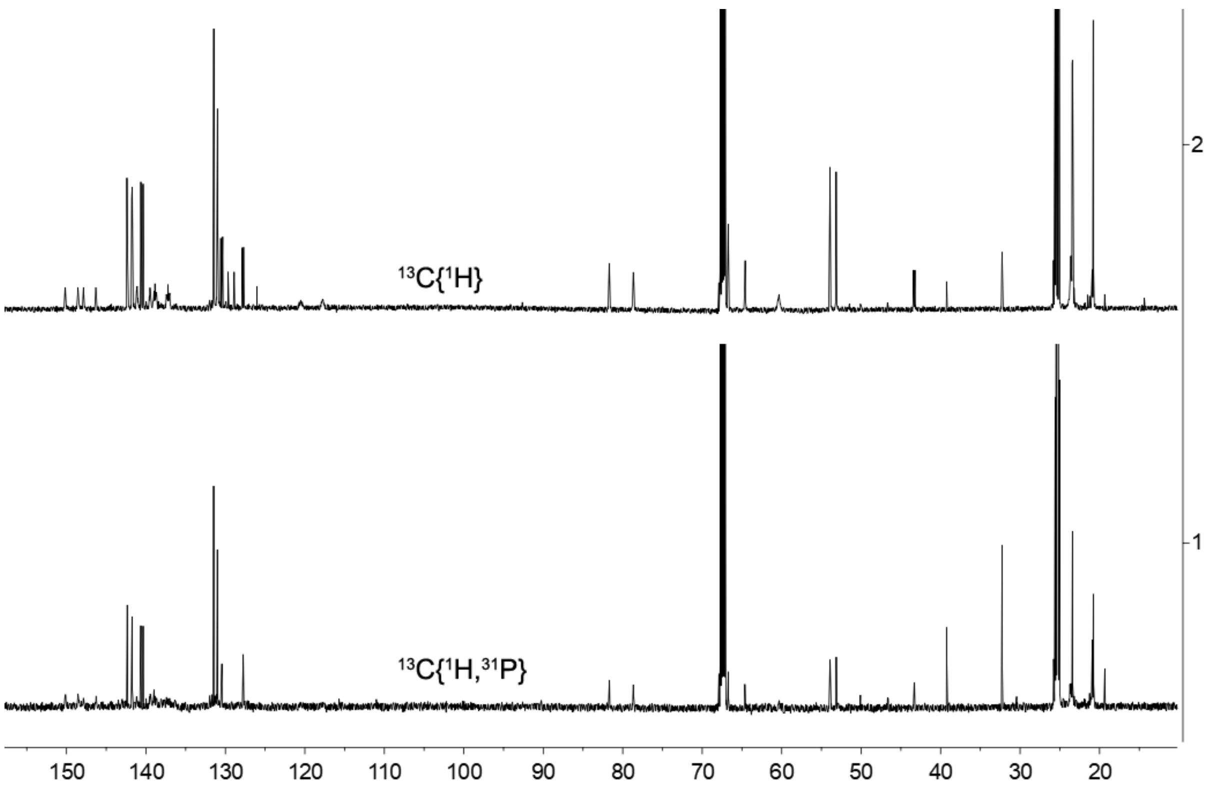

Figure S30. (1) ^{13}**C{^{1}H,^{31}P} NMR** (151 MHz, THF-d$_8$, 299 K) and (2) ^{13}**C{^{1}H} NMR** spectra of compound **12**.

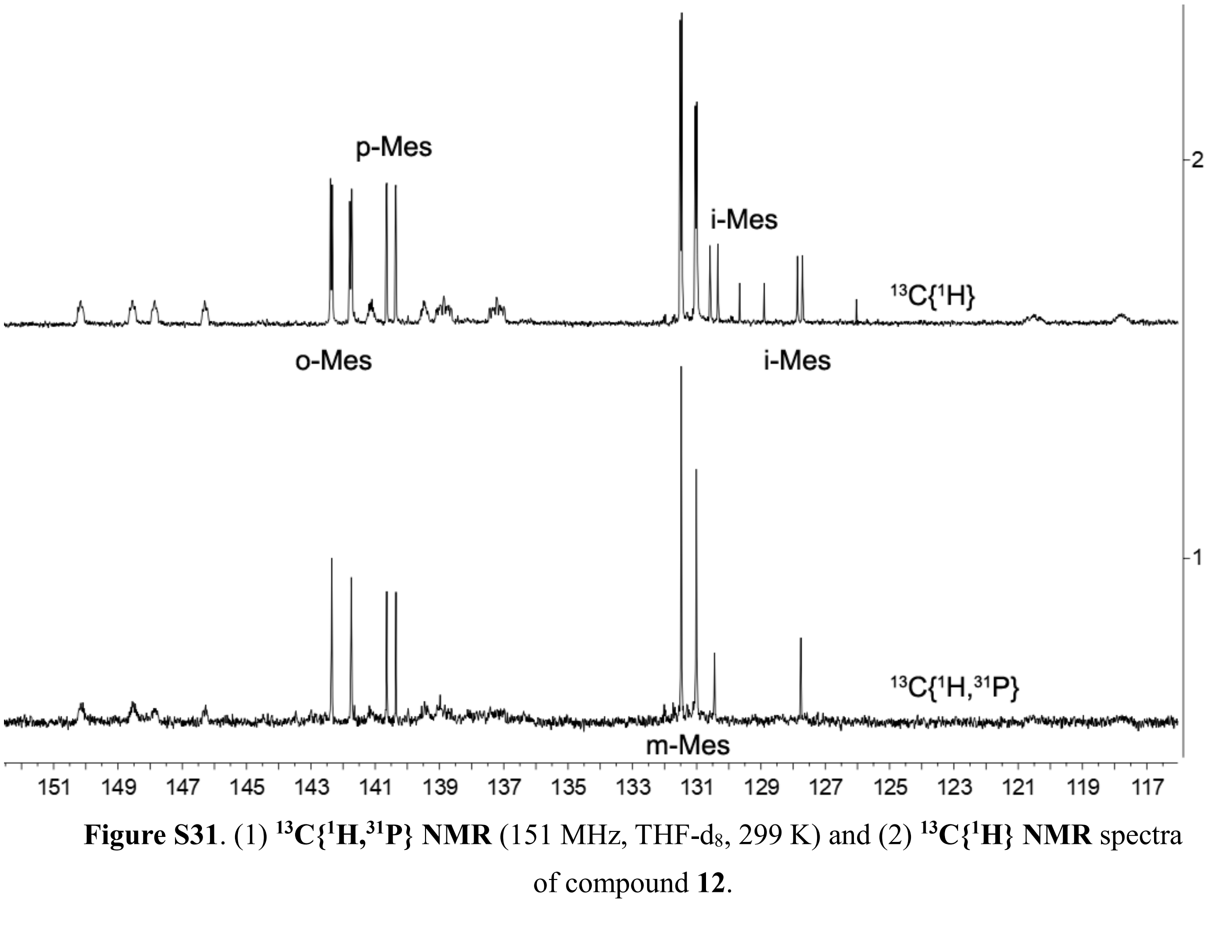

Figure S31. (1) ^{13}**C{^{1}H,^{31}P} NMR** (151 MHz, THF-d$_8$, 299 K) and (2) ^{13}**C{^{1}H} NMR** spectra of compound **12**.

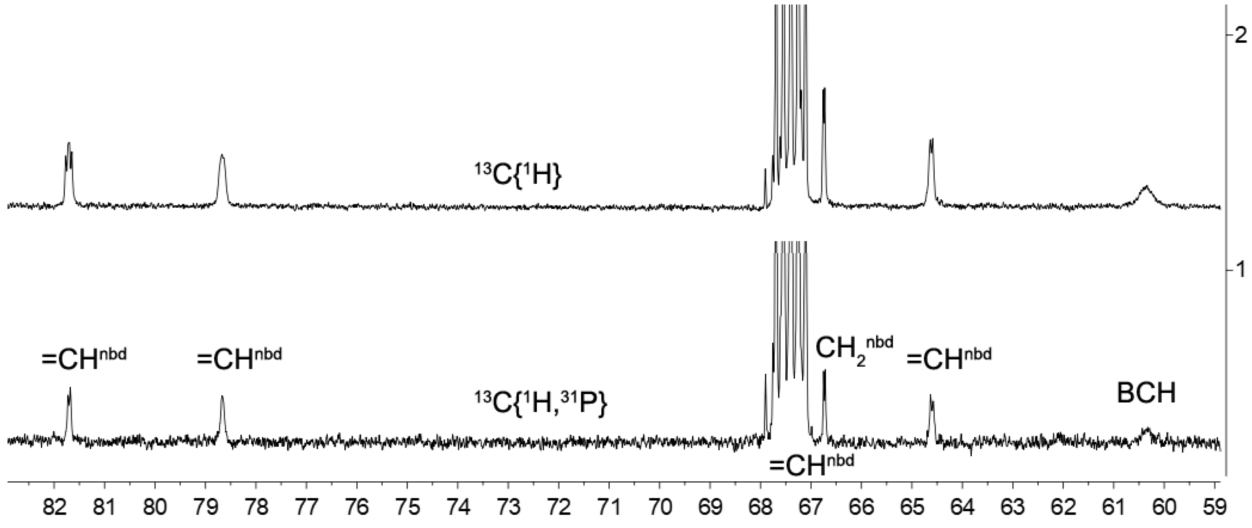

Figure S32. (1) **$^{13}C\{^{1}H,^{31}P\}$ NMR** (151 MHz, THF-d$_8$, 299 K) and (2) **$^{13}C\{^{1}H\}$ NMR** spectra of compound **12**.

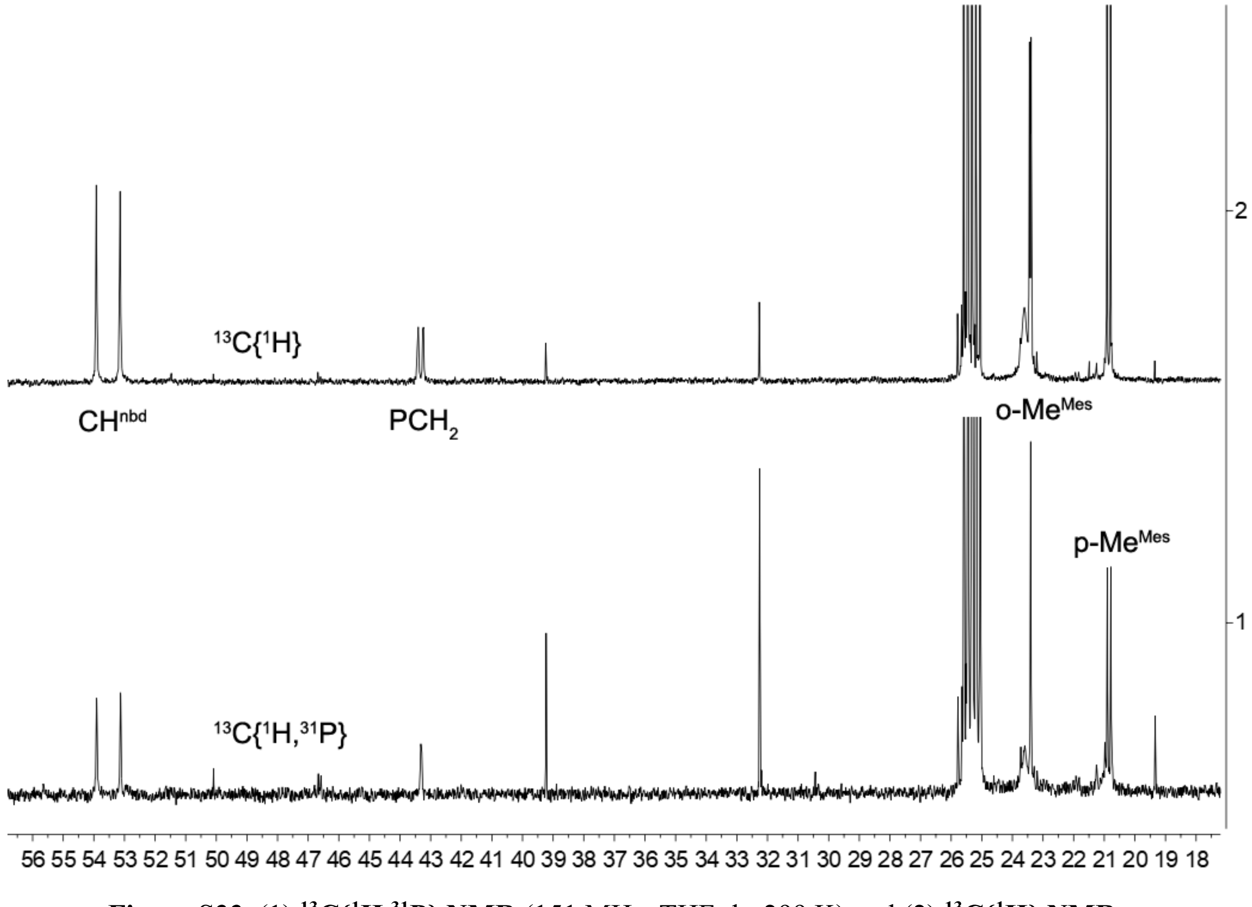

Figure S33. (1) **$^{13}C\{^{1}H,^{31}P\}$ NMR** (151 MHz, THF-d$_8$, 299 K) and (2) **$^{13}C\{^{1}H\}$ NMR** spectra of compound **12**.

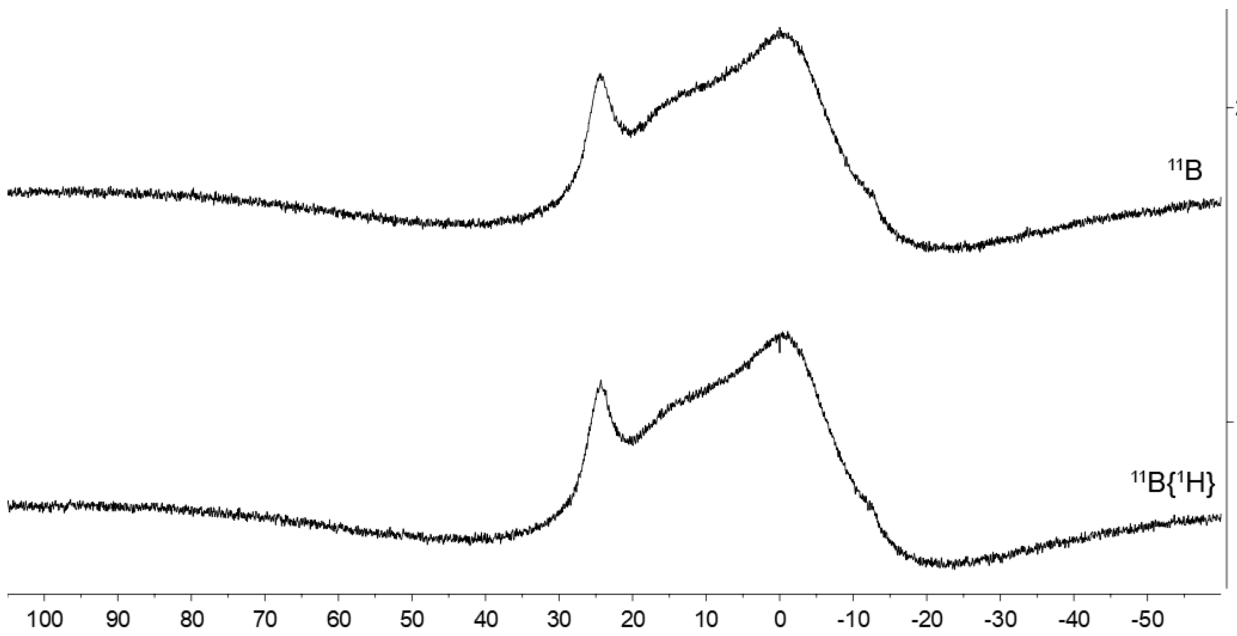

Figure S34. (1) **^{11}B{^{1}H}** and (2) **^{11}B NMR** (192 MHz, THF-d_8, 299 K) spectrum of compound **12**.

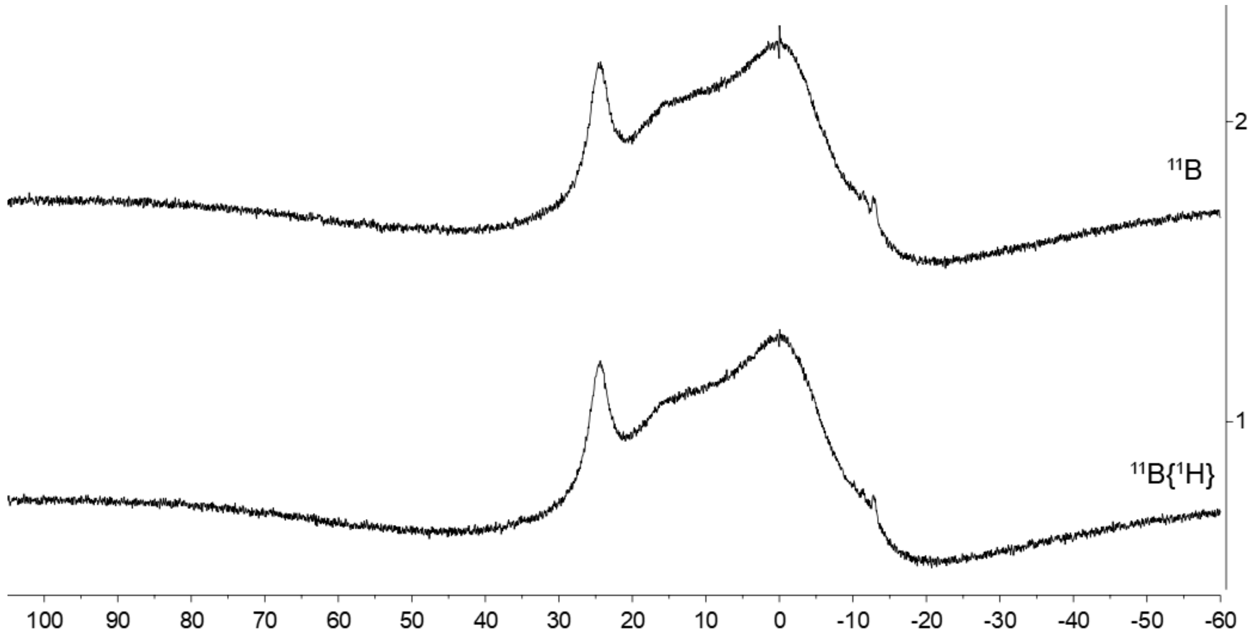

Figure S35. (1) **^{11}B{^{1}H}** and (2) **^{11}B NMR** (192 MHz, CD_2Cl_2, 299 K) spectrum of compound **12**.

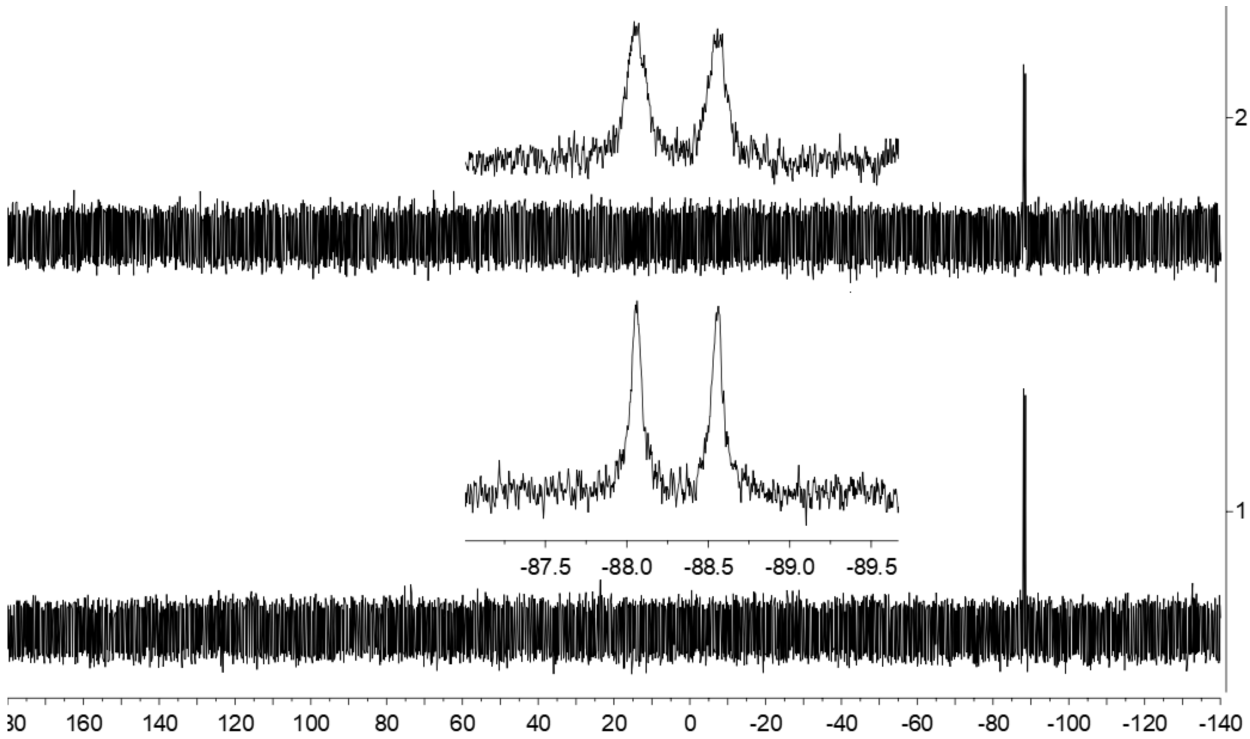

Figure S36. (1) **^{31}P{^{1}H}** and (2) **^{31}P NMR** (243 MHz, THF-d$_8$, 299 K) spectra of compound **12**.

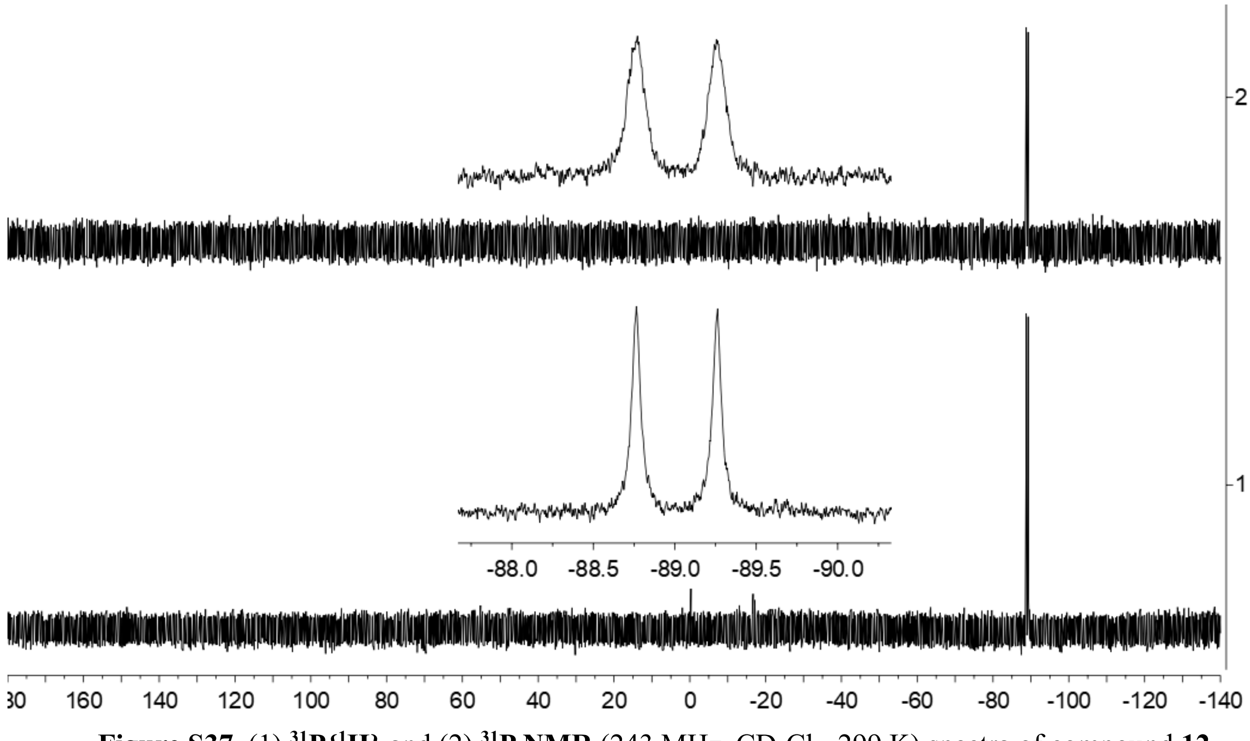

Figure S37. (1) **^{31}P{^{1}H}** and (2) **^{31}P NMR** (243 MHz, CD_2Cl_2, 299 K) spectra of compound **12**.

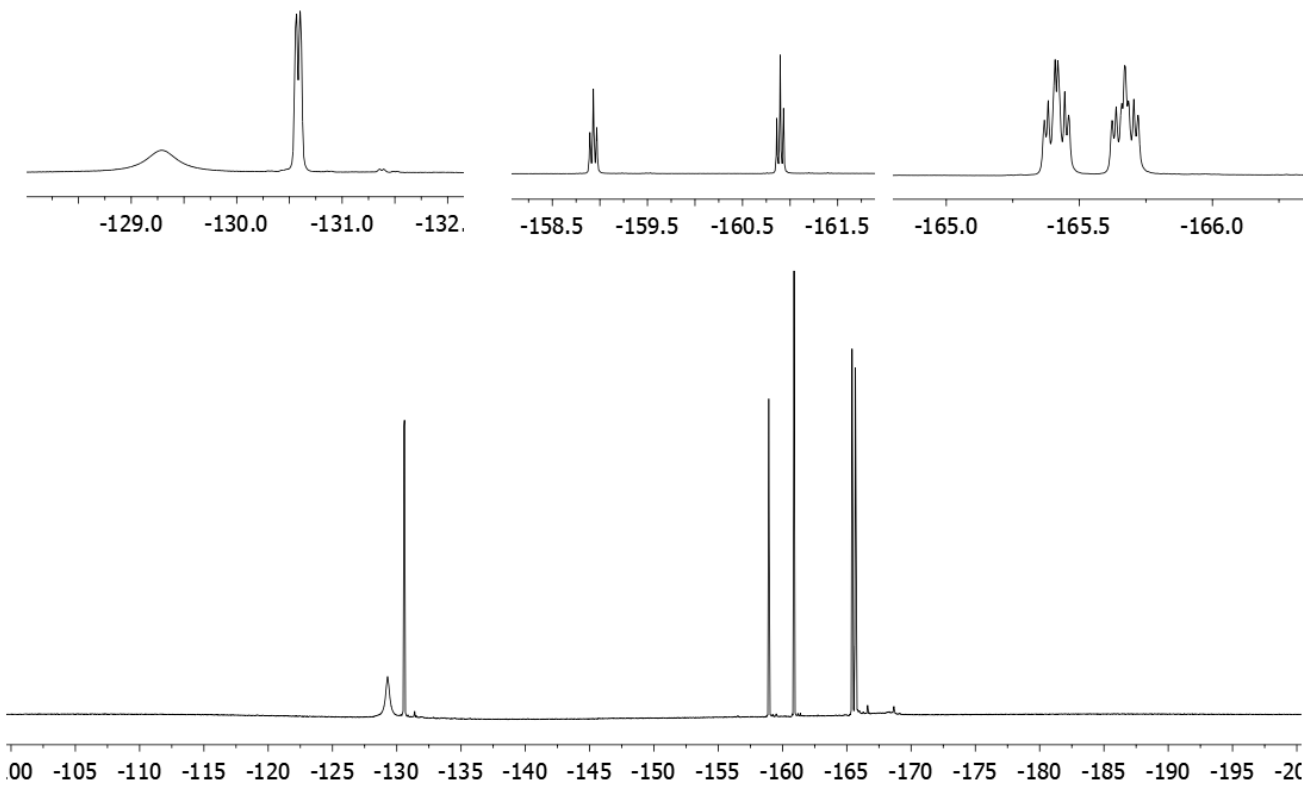

Figure S38. **^{19}F NMR** (564 MHz, THF-d_8 299 K) spectra of compound **12**.

Crystals suitable for the X-ray crystal structure analysis were obtained from slow diffusion of pentane into a saturated solution of compound **12** in CH_2Cl_2 at -30 °C.

X-ray crystal structure analysis of compound 12 (erk9354): A yellow needle-like specimen of $C_{39}H_{33}BF_{10}PRh$, approximate dimensions 0.052 mm x 0.142 mm x 0.242 mm, was used for the X-ray crystallographic analysis. The X-ray intensity data were measured. A total of 832 frames were collected. The total exposure time was 5.78 hours. The frames were integrated with the Bruker SAINT software package using a narrow-frame algorithm. The integration of the data using a monoclinic unit cell yielded a total of 71688 reflections to a maximum θ angle of 27.56° (0.77 Å resolution), of which 7718 were independent (average redundancy 9.288, completeness = 99.4%, R_{int} = 6.50%, R_{sig} = 3.46%) and 6353 (82.31%) were greater than 2σ(F^2). The final cell constants of a = 11.3860(4) Å, b = 24.4438(8) Å, c = 12.1411(4) Å, β = 95.5790(10)°, volume = 3363.1(2) Å^3, are based upon the refinement of the XYZ-centroids of 9804 reflections above 20 σ(I) with 4.740° < 2θ < 54.71°. Data were corrected for absorption effects using the multi-scan method (SADABS). The ratio of minimum to maximum apparent transmission was 0.945. The calculated minimum and maximum transmission coefficients (based on crystal size) are 0.8600 and 0.9670. The structure was solved and refined using the Bruker SHELXTL Software Package, using the space group $P2_1/c$, with Z = 4 for the formula unit, $C_{39}H_{33}BF_{10}PRh$. The final anisotropic full-matrix least-squares refinement on F^2 with 475 variables converged at R1 = 3.22%, for the observed data and wR2 = 6.88% for all data. The goodness-of-fit was 1.050. The largest peak in the final difference electron density synthesis was 0.519 e^-/Å^3 and the largest hole was -0.526 e^-/Å^3 with

an RMS deviation of 0.083 e$^-$/Å^3. On the basis of the final model, the calculated density was 1.652 g/cm^3 and F(000), 1688 e$^-$. CCDC number: 1960305.

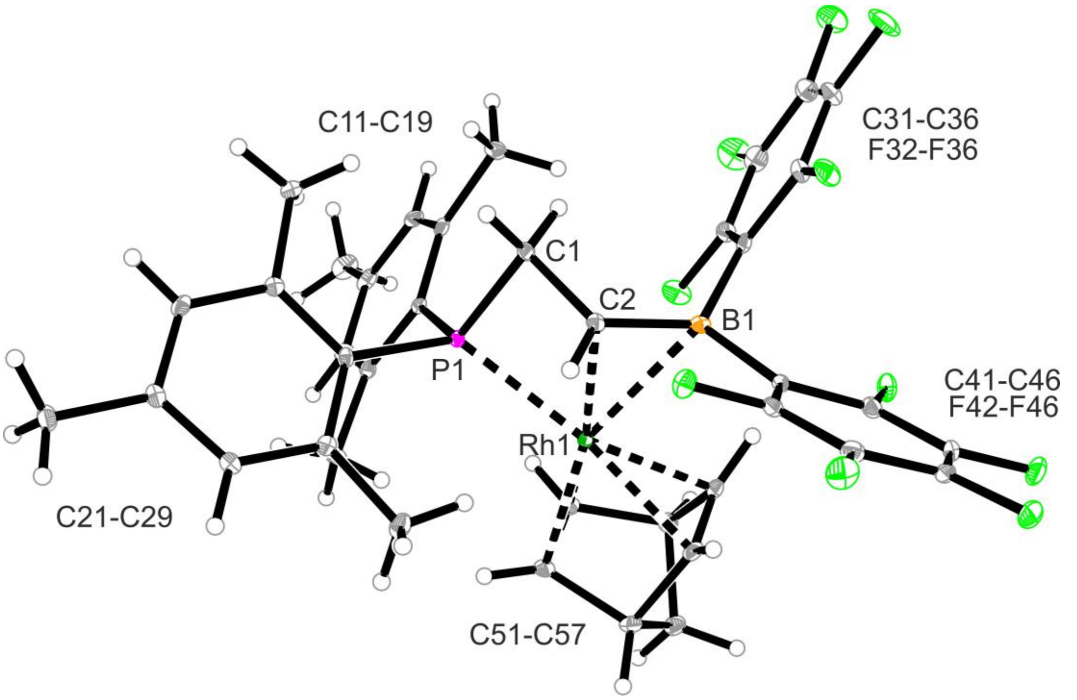

Figure S39. Crystal structure of compound **12** (thermal ellipsoids: 30% probability).

2nd Experiment: A mixture of compound **9** (394.5 mg, 0.5 mmol) and bicyclo[2.2.1]hepta-2,5-diene-rhodium(I) chloride dimer (115.2 mg, 0.25 mmol) in toluene (5 mL) was stirred for 18 hours at room temperature to yield a deep red turbid mixture. Then, the reaction mixture was filtered through Celite plug, and all volatiles of the filtrate were removed *in vacuo*. The obtained red semi-solid material was suspended in pentane (5 mL) and vigorously stirred for 30 minutes to give a suspension. Then the deep red supernatant liquid was decanted and the remaining orange residue was washed with pentane (3x5 mL). The remaining solid residue was dried *in vacuo* to give compound **12** as an orange powder (280 mg, 0.335 mmol, 67% yield).

Elemental Analysis calcd for $C_{39}H_{33}BF_{10}PRh$ (836.34 g/mol): C, 56.00; H, 3.98; found: C, 55.74; H, 3.68.
^{1}H NMR (600 MHz, CD_2Cl_2, 299 K): δ = [6.94 (br), 6.75 (d, $^4J_{PH}$ = 3.0 Hz)](each 2H, *m*-Mes), [4.88, 4.00, 3.84, 3.82](each s, 1H, =CH^{nbd}), [4.14, 3.90](each m, 1H, PCH_2), [3.80, 3.70](each s, 1H, CH^{nbd}), 3.68 (m, 1H, BCH), [2.61 (br), 2.16 (s)] (each 6H, *o*-Me^{Mes}), [2.25, 2.17](each s, 3H, *p*-Me^{Mes}), [1.29, 1.07] (each s, 1H, CH_2^{nbd}).
^{13}C{^{1}H} NMR (151 MHz, CD_2Cl_2, 299 K): δ = [141.8 (d, $^2J_{PC}$ = 8.7 Hz), 141.3 (d, $^2J_{PC}$ = 9.1 Hz)](*o*-Mes), [140.1 (d, $^4J_{PC}$ = 1.9 Hz), 139.9 (d, $^4J_{PC}$ = 2.0 Hz)](*p*-Mes), [131.0 (d, $^3J_{PC}$ = 7.4 Hz), 130.4 (d, $^3J_{PC}$ = 7.3 Hz)](*m*-Mes), [129.7 (d, $^1J_{PC}$ = 35.9 Hz), 127.3 (d, $^1J_{PC}$ = 35.9 Hz)](*i*-Mes), [81.1 (m), 78.0 (m), 66.7 (d, *J* = 9.1 Hz), 64.5 (d, *J* = 8.7 Hz)](=CH^{nbd}), 66.5 (d, $^3J_{RhC}$ = 4.7 Hz, CH_2^{nbd}), 59.8 (br, BCH), [53.3, 52.6](CH^{nbd}), 42.9 (dd, *J* = 25.7 Hz, 4.3 Hz, PCH_2), [23.5 (br), 20.9 (d, $^3J_{PC}$ = 19.0 Hz](*o*-Me^{Mes}), [23.3, 23.2](*p*-Me^{Mes}) [C_6F_5 not listed].
^{11}B NMR (192 MHz, CD_2Cl_2, 299 K): δ = 24.3 ($\nu_{1/2}$ ~ 600 Hz).

^{31}P NMR (243 MHz, CD_2Cl_2, 299 K): δ = -89.0 (d, $^{1}J_{RhP}$ ~ 120 Hz).

^{19}F NMR (564 MHz, CD_2Cl_2, 299 K): δ = [-129.2 (br), -130.2 (m)](each 2F, *o*-C_6F_5), [-157.8 (t, $^{3}J_{FF}$ = 20.4 Hz), -159.8 (t, $^{3}J_{FF}$ = 20.1 Hz)](each 1F, *p*-C_6F_5), [-164.6 (m), -164.9 (m)](each 2F, *m*-C_6F_5)

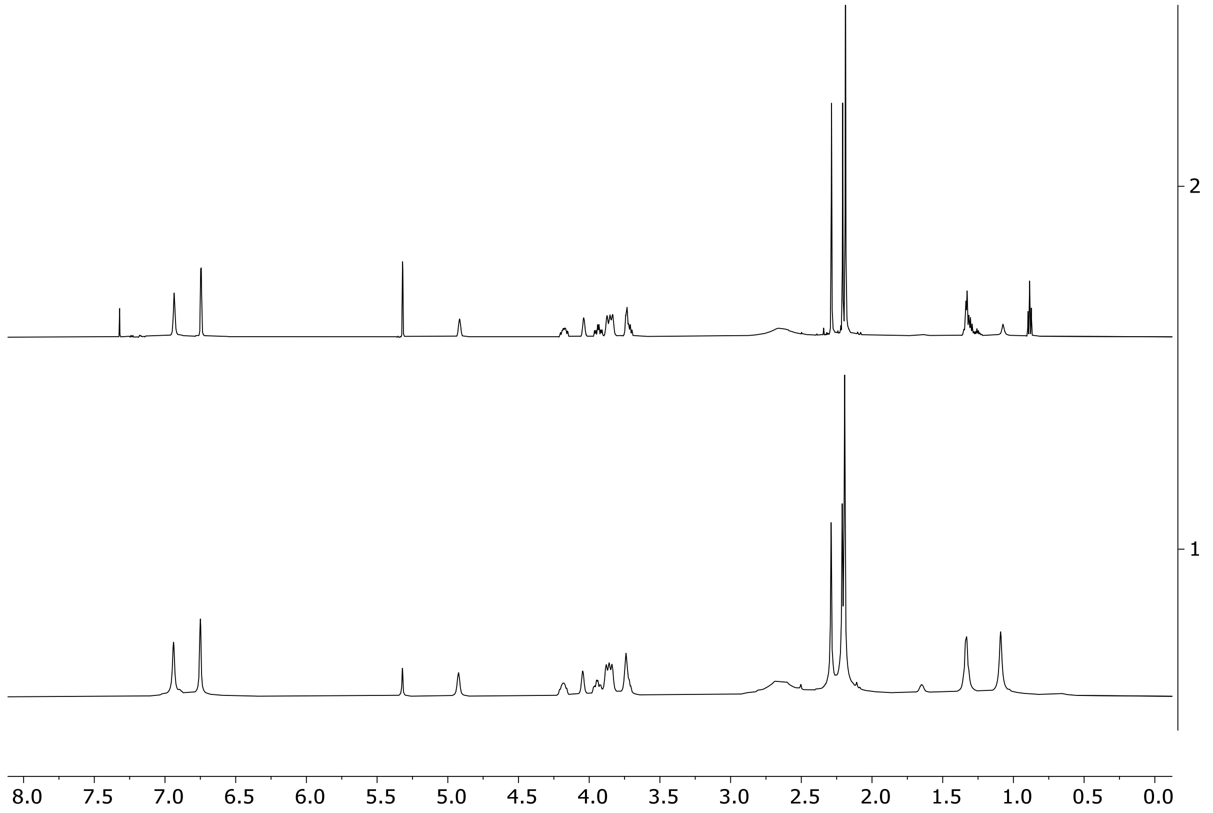

Figure 40. ^{1}H NMR (600 MHz, CD_2Cl_2, 299 K) spectra of compound **12**: (1) 1st experiment, (2) 2nd experiment.

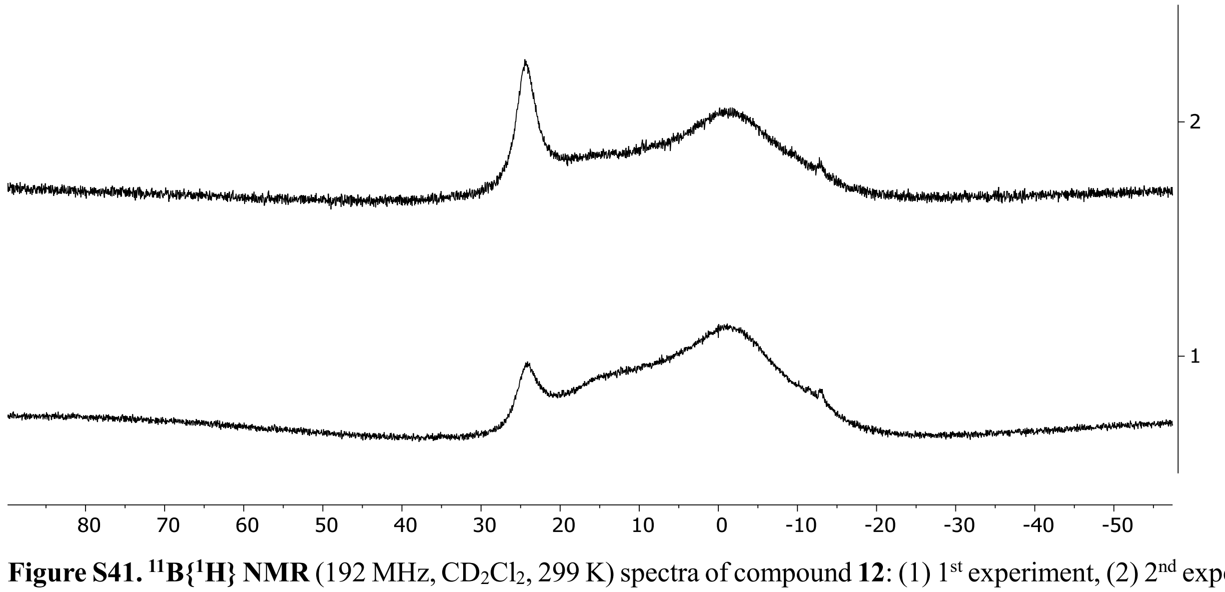

Figure S41. ^{11}B{^{1}H} NMR (192 MHz, CD_2Cl_2, 299 K) spectra of compound **12**: (1) 1st experiment, (2) 2nd experiment.

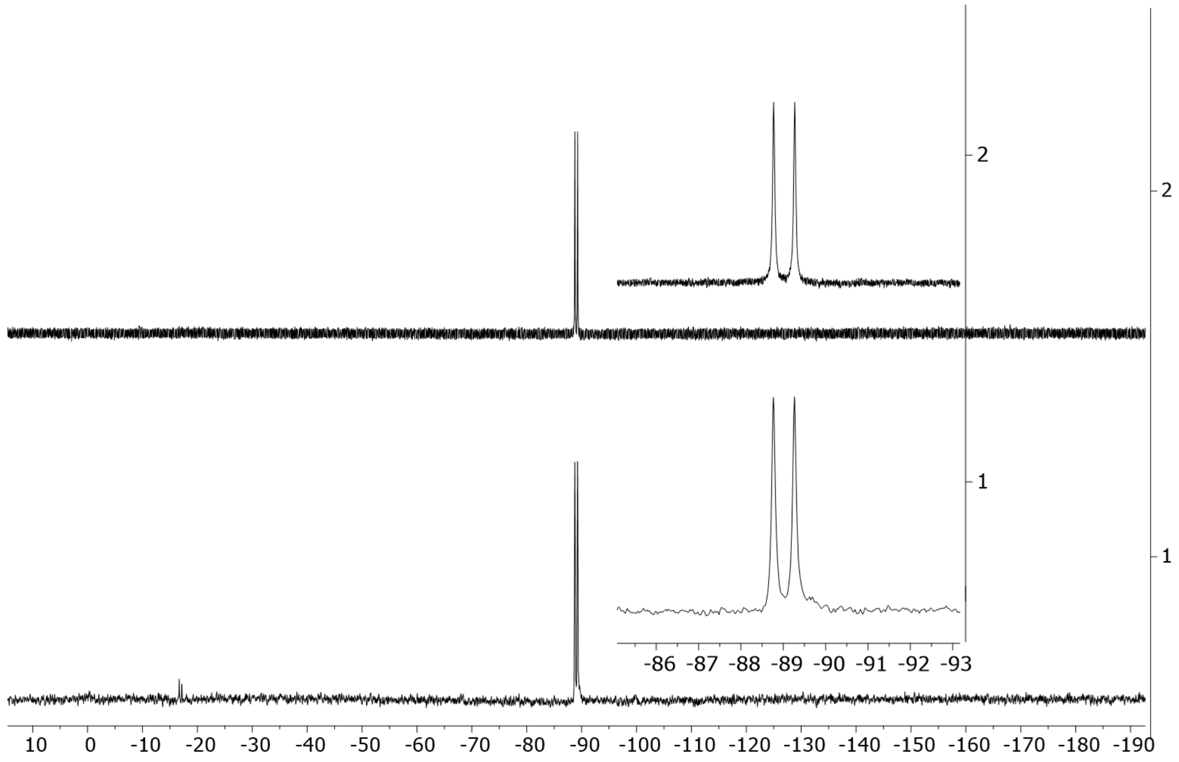

Figure S42. **$^{31}P\{^{1}H\}$ NMR** (243 MHz, CD_2Cl_2, 299 K) spectra of compound **12**: (1) 1st experiment, (2) 2nd experiment.

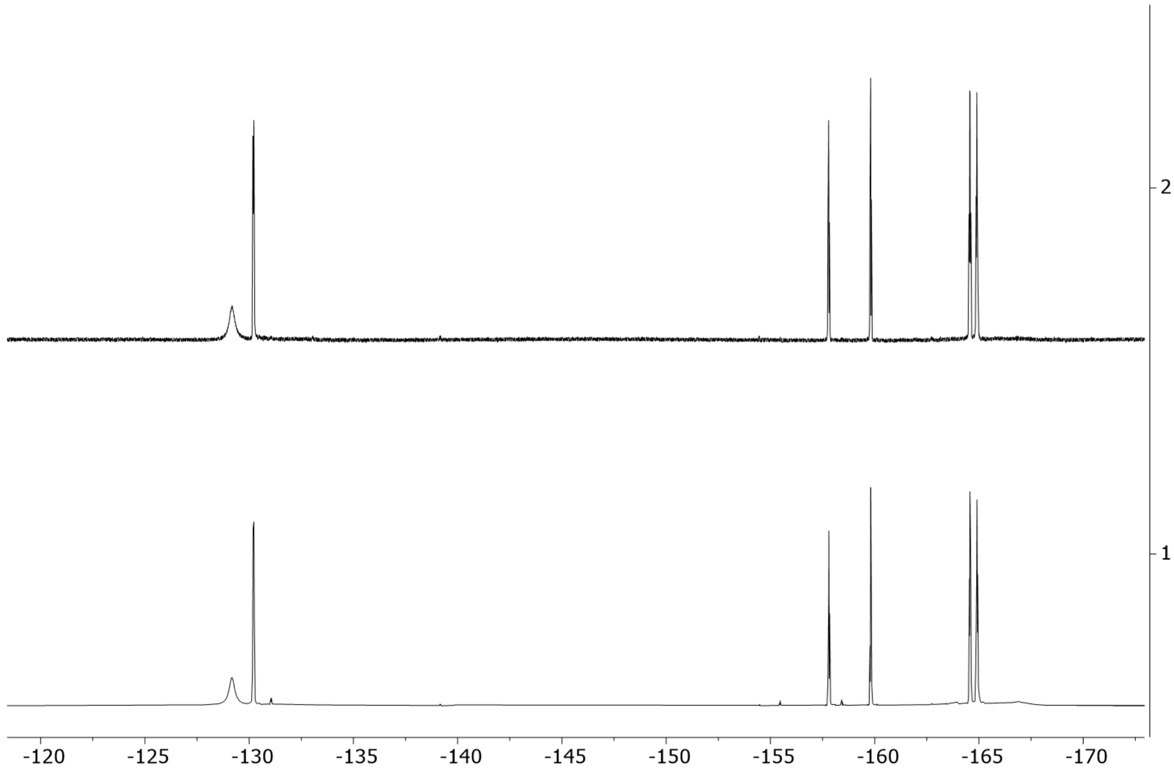

Figure S43. ^{19}F NMR (564 MHz, CD_2Cl_2, 299 K) spectra of compound **12**: (1) 1st experiment, (2) 2nd experiment.

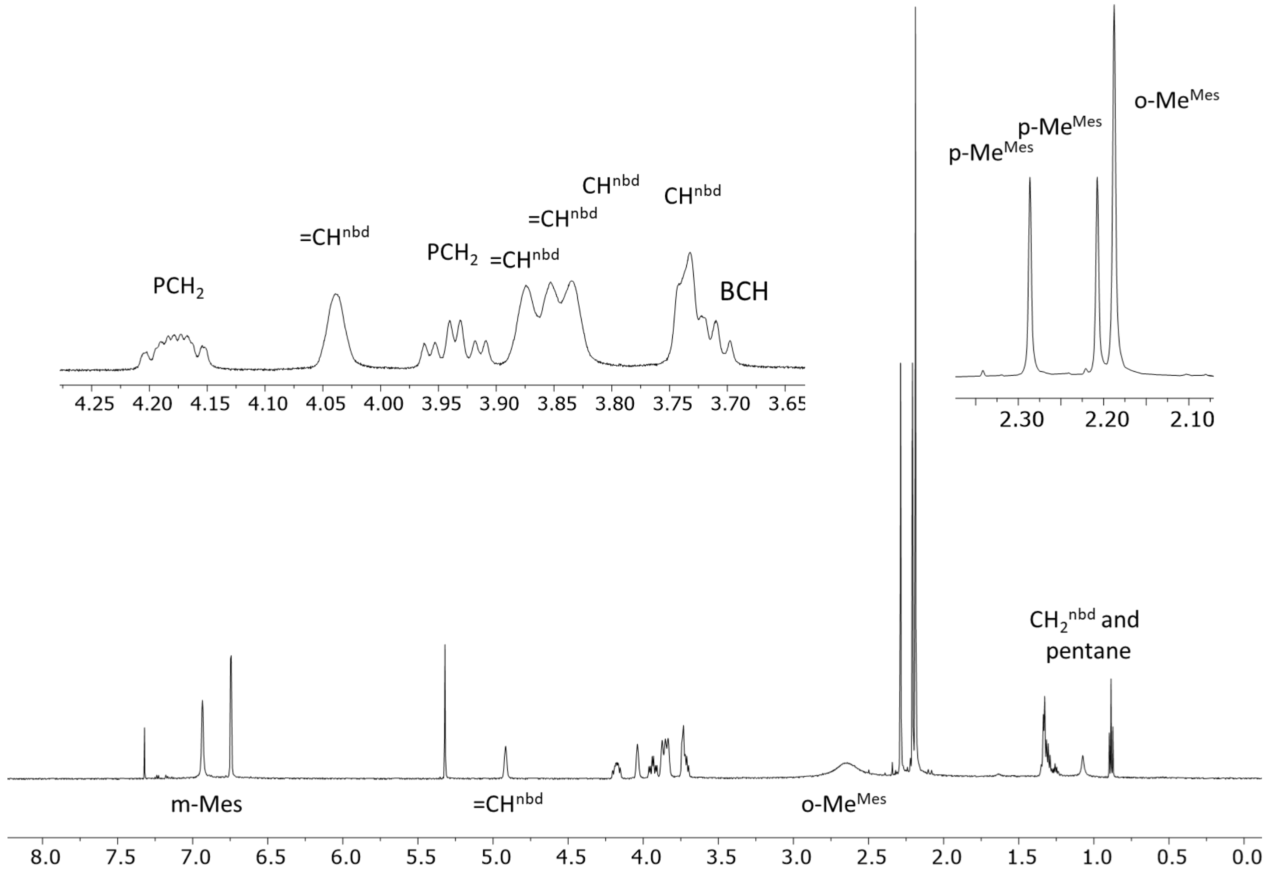

Figure S44. **^{1}H NMR** (600 MHz, CD_2Cl_2, 299 K) spectrum of compound **12**: 2nd experiment.

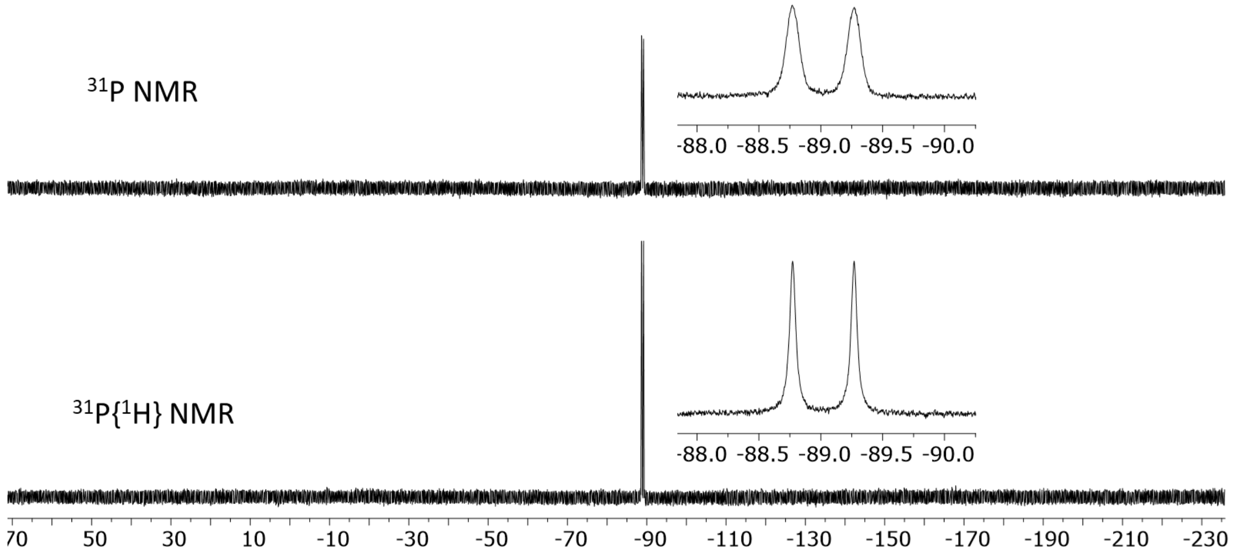

Figure S45. **^{31}P** and **^{31}P{^{1}H} NMR** (243 MHz, CD_2Cl_2, 299 K) spectra of compound **12**: 2nd experiment.

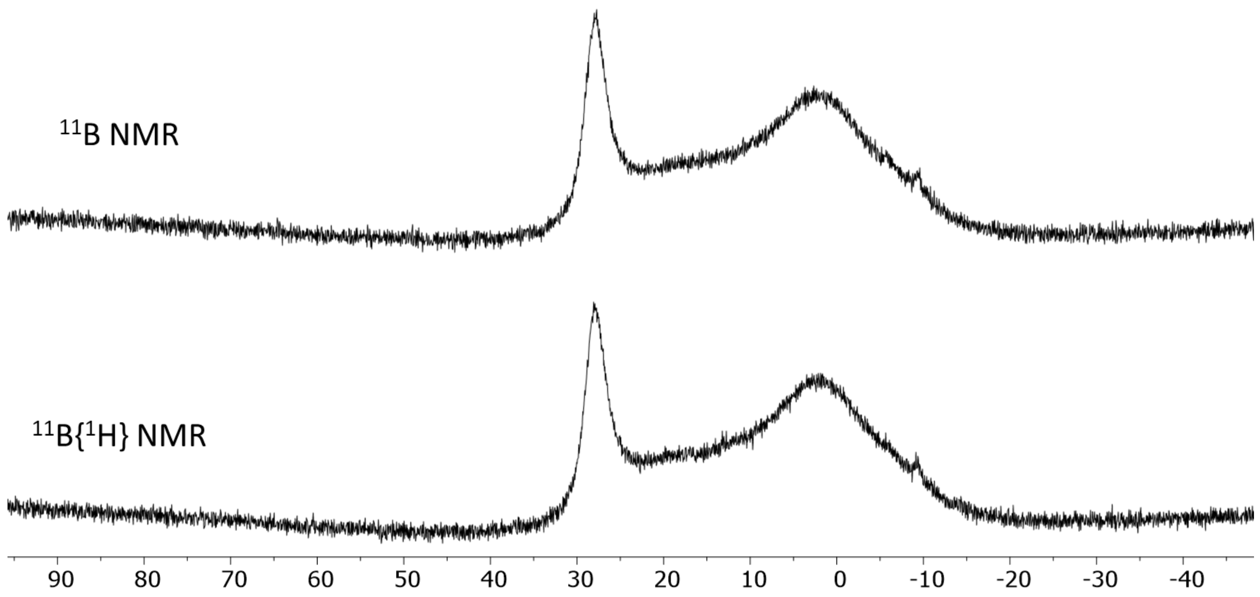

Figure S46. ^{11}B and **^{11}B{^{1}H} NMR** (192 MHz, CD_2Cl_2, 299 K) spectra of compound **12**: 2nd experiment.

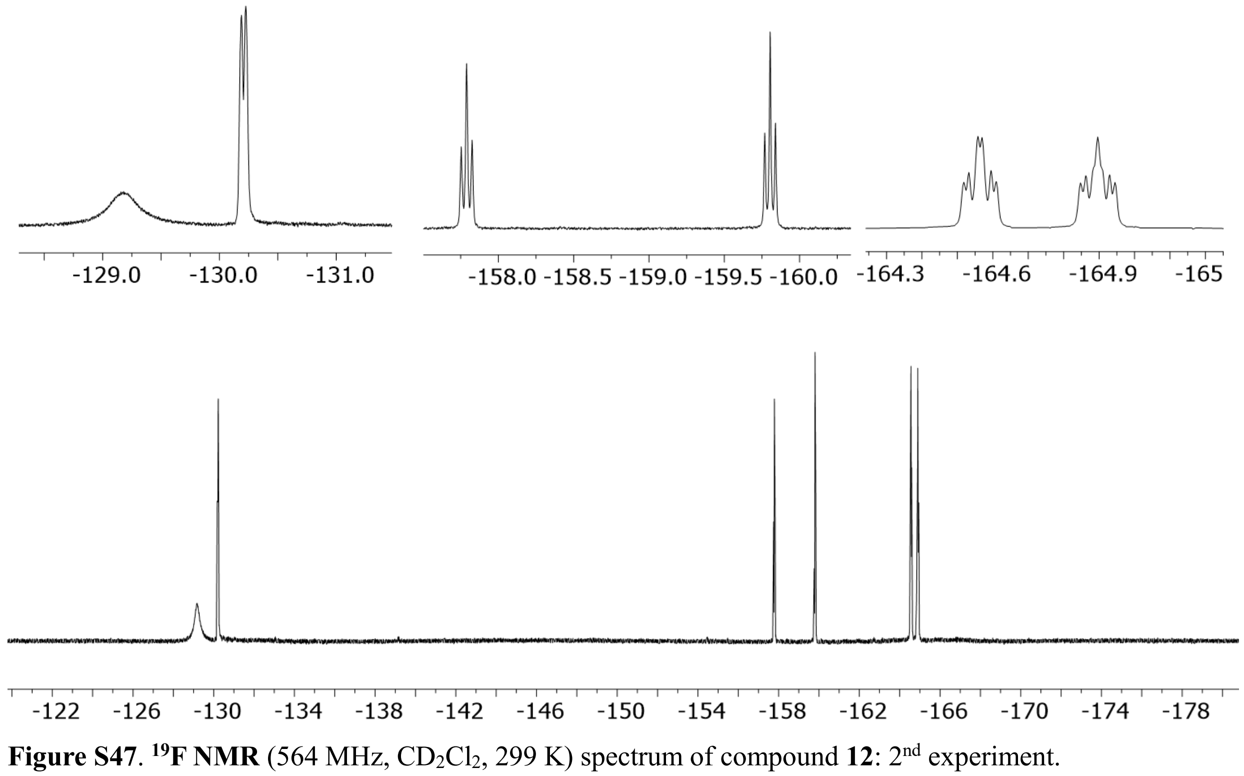

Figure S47. **^{19}F NMR** (564 MHz, CD_2Cl_2, 299 K) spectrum of compound **12**: 2nd experiment.

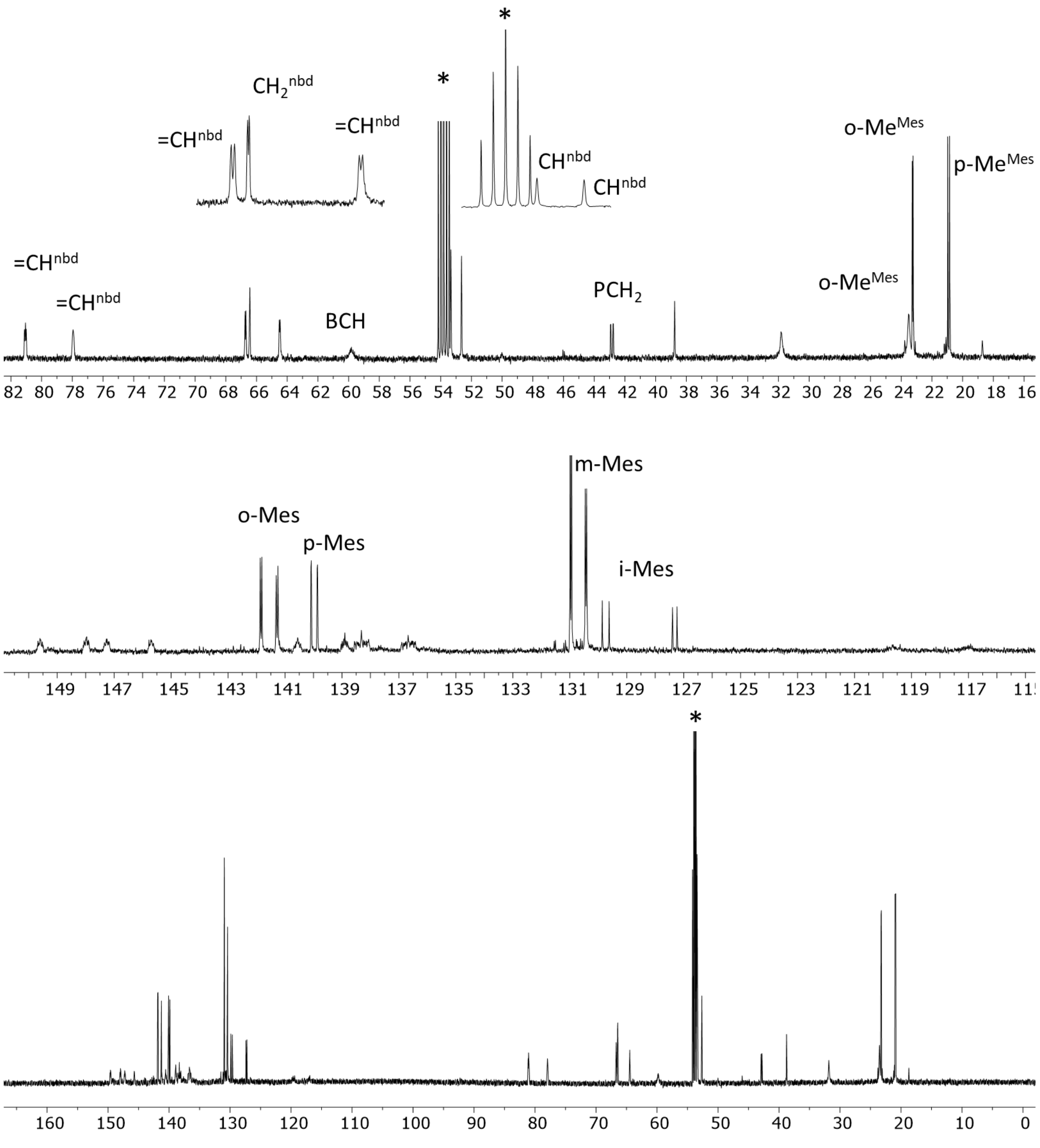

Figure S48. **$^{13}C\{^1H\}$ NMR** (151 MHz, CD_2Cl_2*, 299 K) spectrum of compound **12**: 2nd experiment.

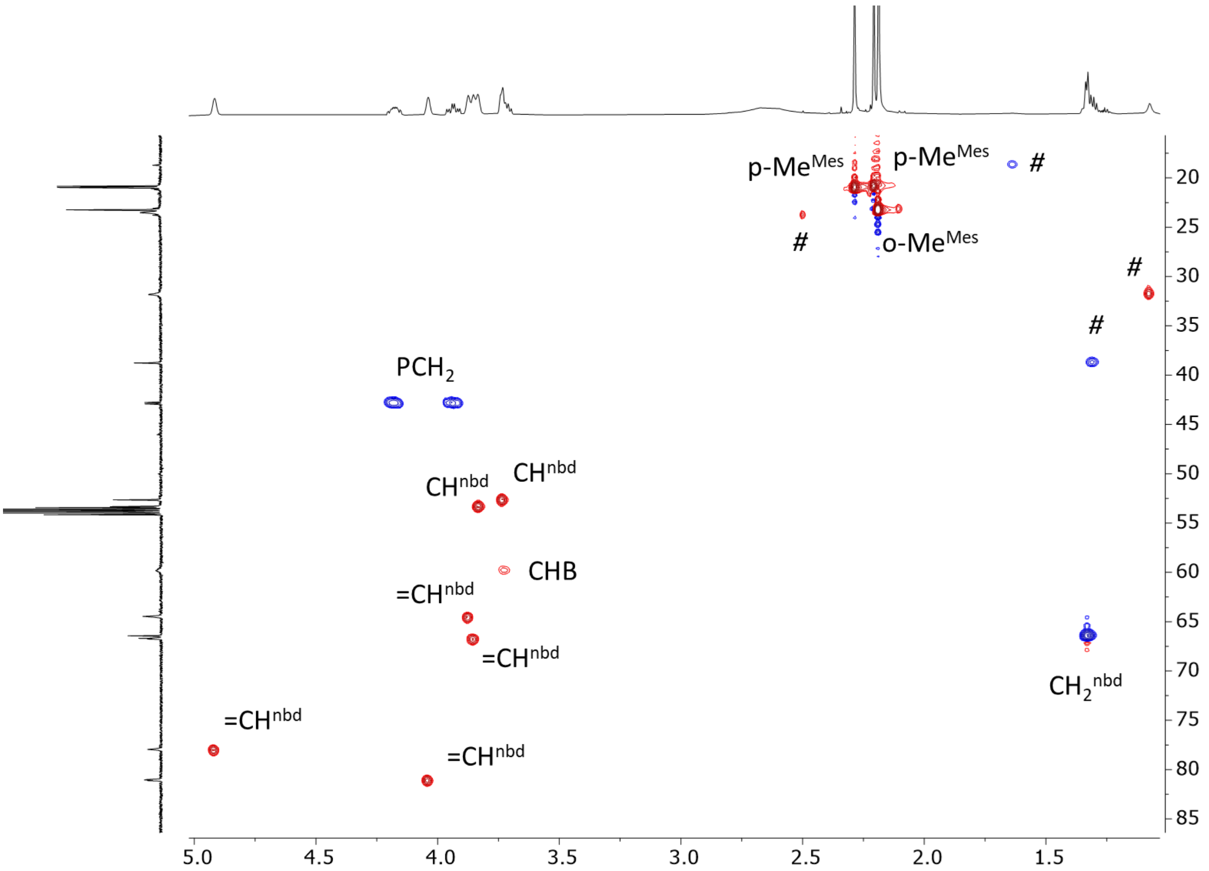

Figure S49. ^{1}**H,**^{13}**C{**^{1}**H} ghsqc** (600 /151 MHz, CD_2Cl_2, 299 K) of compound **12**: 2nd experiment. # denotes crosspeaks due to impurities.

Preparation of compound 13

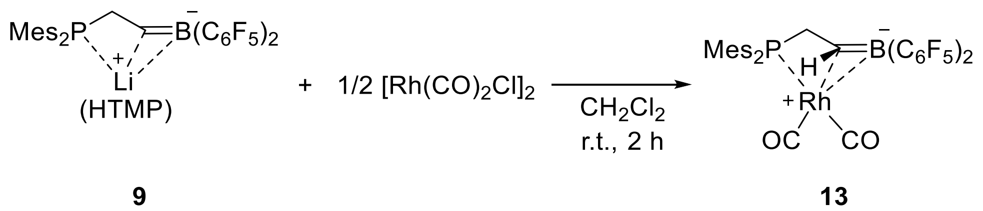

Scheme S6

A mixture of compound **9** (78.9 mg, 0.1 mmol) and di-μ-chlorotetracarbonyldirhodium(I) (19.4 mg, 0.05 mmol) were combined in dichloromethane (4 mL) and stirred for 2 hours at room temperature. Then, all volatilities were removed in vacuo. Subsequently pentane (ca 4 mL) was added to the obtained residue. The resulting mixture was filtered and stored in a freezer (-35°C). Yellow crystals of the product formed over several days. The solid was collected by decantation, washed with a small amount of cold pentane and dried in vacuo. The product was isolated as a yellow solid (36.8 mg, 0.046 mmol, 46% yield).

NOTE: Attempts to prepare complex **13** starting from the (nbd)Rh derivative **12** by treatment with CO gas (1 atm) in C_6D_6 were not successful.

Melting point: 120.3 °C

Elemental Analysis calcd for $C_{34}H_{25}BF_{10}O_2PRh$ (800.23 g/mol): C, 51.03; H, 3.15; found: C, 50.38; H, 3.42.

HRMS (ESI): m/z calcd for [$C_{34}H_{25}BF_{10}O_2PRh+H^+$] 801.0659; found: 801.0657.

IR (ATR) ν (cm^{-1}) = 2069 (s), 1997 (s)

^{1}H NMR (600 MHz, CD_2Cl_2, 299 K): δ = [7.00 (d, $^4J_{PH}$ = 3.3 Hz), 6.80 (d, $^4J_{PH}$ = 3.6 Hz](each 2H, *m*-Mes), [4.41, 3.86](each m, each 1H, PCH_2), 3.18 (m, 1H, BCH), [2.58, 2.12](each s, each 6H, *o*-Me^{Mes}), [2.32, 2.23](each s, each 3H, *p*-Me^{Mes}).

^{13}C{^{1}H} NMR (151 MHz, CD_2Cl_2, 299 K)[selected resonances]: δ = 184.8 (dd, $^1J_{RhC}$ = 70.0 Hz, $^2J_{PC}$ = 3.7 Hz, CO), [142.5 (d, $^2J_{PC}$ = 8.8 Hz), 140.8 (d, $^2J_{PC}$ = 9.7 Hz)](o-Mes), [141.9 (d, $^4J_{PC}$ = 2.1 Hz), 141.0 (d, $^4J_{PC}$ = 2.4 Hz)](p-Mes), [131.4 (d, $^3J_{PC}$ = 8.4 Hz), 131.3 (d, $^3J_{PC}$ = 8.7 Hz)](*m*-Mes), [128.0 (d,), 126.1 (d, $^1J_{PC}$ = 33.5 Hz)](i-Mes), 49.1 (br, BCH), 40.4 (dd, J = 27.7 Hz, J = 4.3 Hz, PCH_2), [24.1 (d, $^3J_{PC}$ = 8.2 Hz), 23.6 (d, $^3J_{PC}$ = 7.5 Hz)](*o*-Me^{Mes}), [21.1, 20.9](*p*-Me^{Mes}).

^{11}B{^{1}H} NMR (192 MHz, CD_2Cl_2, 299 K): δ = 27.3 ($\nu_{1/2}$ ~ 650 Hz)

^{31}P{^{1}H} NMR (243 MHz, CD_2Cl_2, 299 K): δ = -105.0 (d, $^1J_{RhP}$ = 88.5 Hz)

^{19}F NMR (564 MHz, CD_2Cl_2, 299 K): δ [-128.3, -129.0](each m, each 2F, *o*-C_6F_5), [-155.7 (tt, $^3J_{FF}$ = 20.4 Hz, $^4J_{FF}$ = 3.5 Hz), -157.7 (t, $^3J_{FF}$ = 20.1 Hz)](each 1F, *p*-C_6F_5), [-164.0, -164.2](each m, each 2F, *m*-C_6F_5)

Comment: The complex decomposed during the ^{13}C{^{1}H} NMR measurement (CD_2Cl_2).

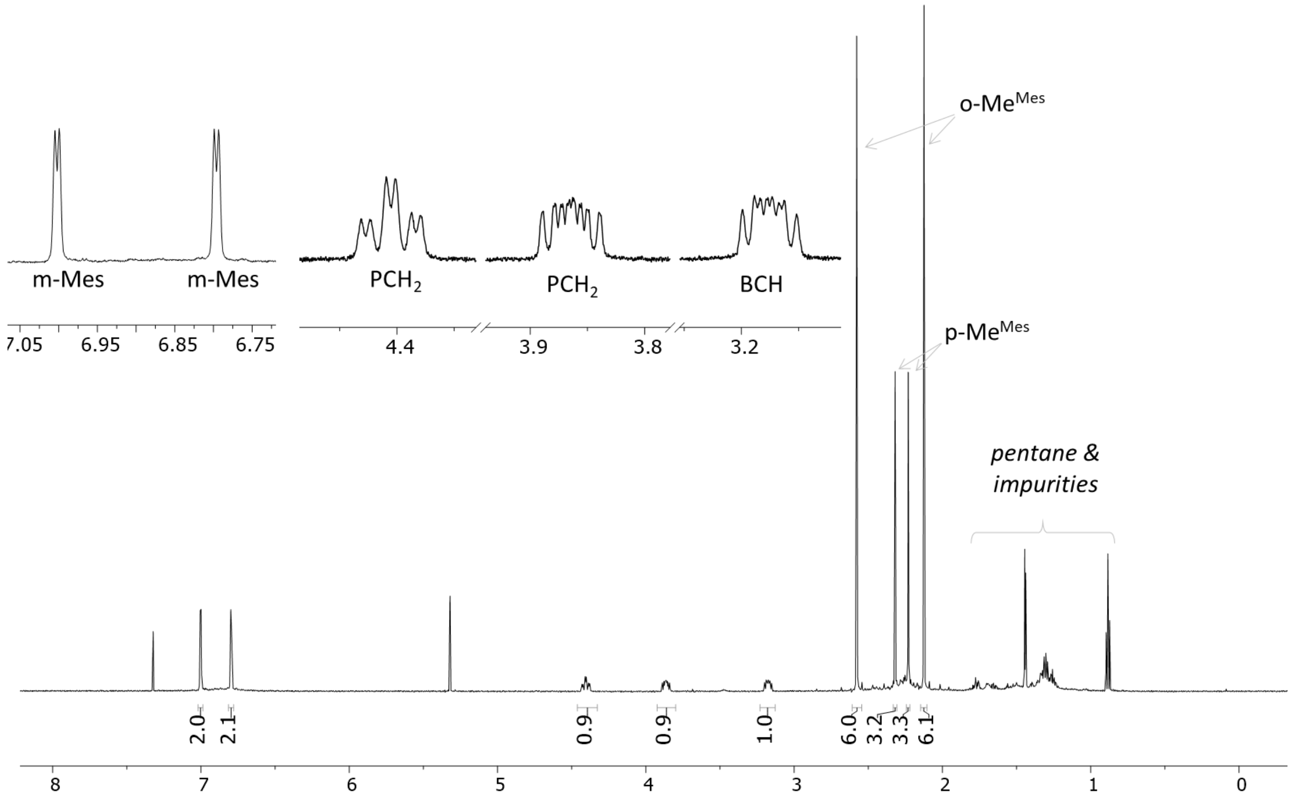

Figure S50. **^{1}H NMR** (600 MHz, CD_2Cl_2, 299 K) spectrum of compound **13**.

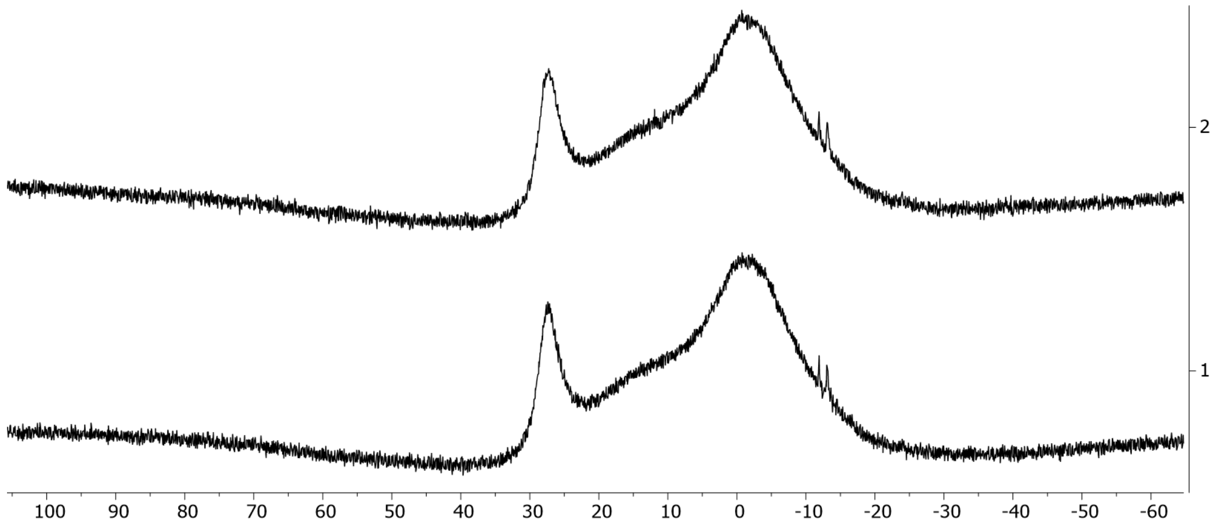

FigureS51. (1) **^{11}B{^{1}H}** and (2) **^{11}B NMR** (192 MHz, CD_2Cl_2, 299 K) spectra of compound **13**.

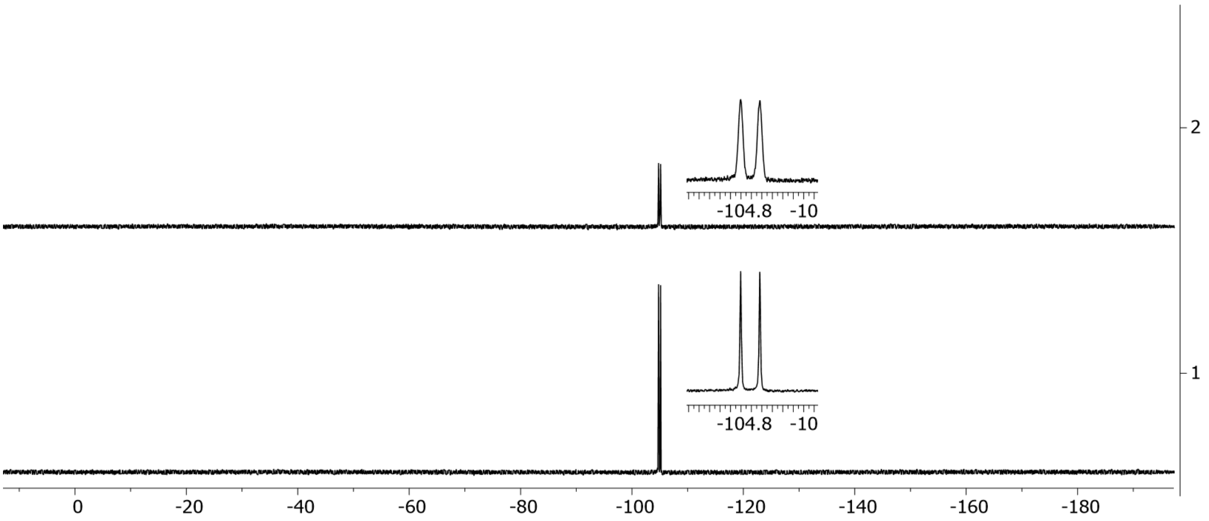

Figure S52. (1) ^{31}**P{^{1}H}** and (2) ^{31}**P NMR** (243 MHz, CD_2Cl_2, 299 K) spectra of compound **13**.

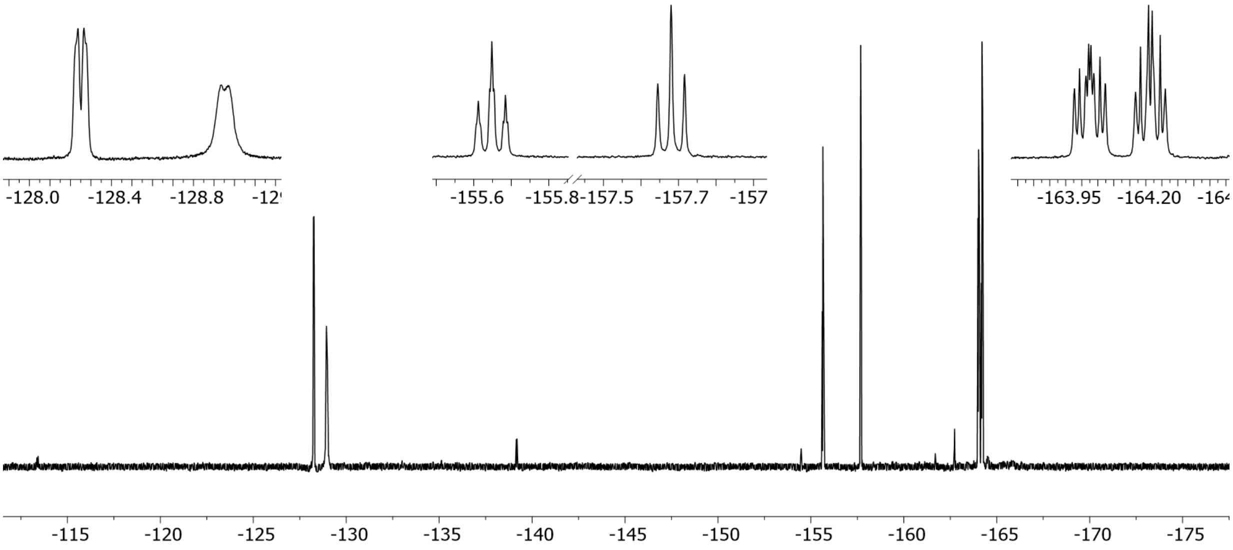

Figure S53. ^{19}**F NMR** (564 MHz, CD_2Cl_2, 299 K) spectrum of compound **13**.

Crystal suitable for X-ray crystal structure analysis were obtained from a solution of compound **13** in pentane at -30°C.

X-ray crystal structure analysis of 13 (erk9844): A yellow plate-like specimen of $C_{39}H_{37}BF_{10}O_2PRh$, approximate dimensions 0.030 mm x 0.099 mm x 0.103 mm, was used for the X-ray crystallographic analysis. The X-ray intensity data were measured on a Bruker D8 Venture PHOTON III Diffractometer system equipped with a micro focus tube Mo ImS (MoKα, λ = 0.71073 Å) and a MX mirror monochromator. A total of 650 frames were collected. The total exposure time was 2.71 hours. The frames were integrated with the Bruker SAINT software package using a narrow-frame algorithm. The integration of the data using a monoclinic unit cell yielded a total of 63397 reflections to a maximum θ angle of 25.35° (0.83 Å resolution), of which 6914 were independent (average redundancy 9.169, completeness = 99.9%, R_{int} = 13.39%, R_{sig} = 8.06%) and 5188 (75.04%) were greater than 2σ(F^2). The final cell

constants of a = 11.1535(6) Å, b = 28.4831(16) Å, c = 12.0712(6) Å, β = 100.231(2)°, volume = 3773.9(3) $Å^3$, are based upon the refinement of the XYZ-centroids of 3915 reflections above 20 σ(I) with 4.465° < 2θ < 42.50°. Data were corrected for absorption effects using the Multi-Scan method (SADABS). The ratio of minimum to maximum apparent transmission was 0.877. The calculated minimum and maximum transmission coefficients (based on crystal size) are 0.9430 and 0.9830. The structure was solved and refined using the Bruker SHELXTL Software Package, using the space group $P2_1/c$, with Z = 4 for the formula unit, $C_{39}H_{37}BF_{10}O_2PRh$. The final anisotropic full-matrix least-squares refinement on F^2 with 500 variables converged at R1 = 4.82%, for the observed data and wR2 = 12.40% for all data. The goodness-of-fit was 1.022. The largest peak in the final difference electron density synthesis was 0.939 $e^-/Å^3$ and the largest hole was -0.659 $e^-/Å^3$ with an RMS deviation of 0.099 $e^-/Å^3$. On the basis of the final model, the calculated density was 1.535 g/cm^3 and F(000), 1768 e^-. The hydrogen at C2 atom was refined freely. CCDC number: 2008240.

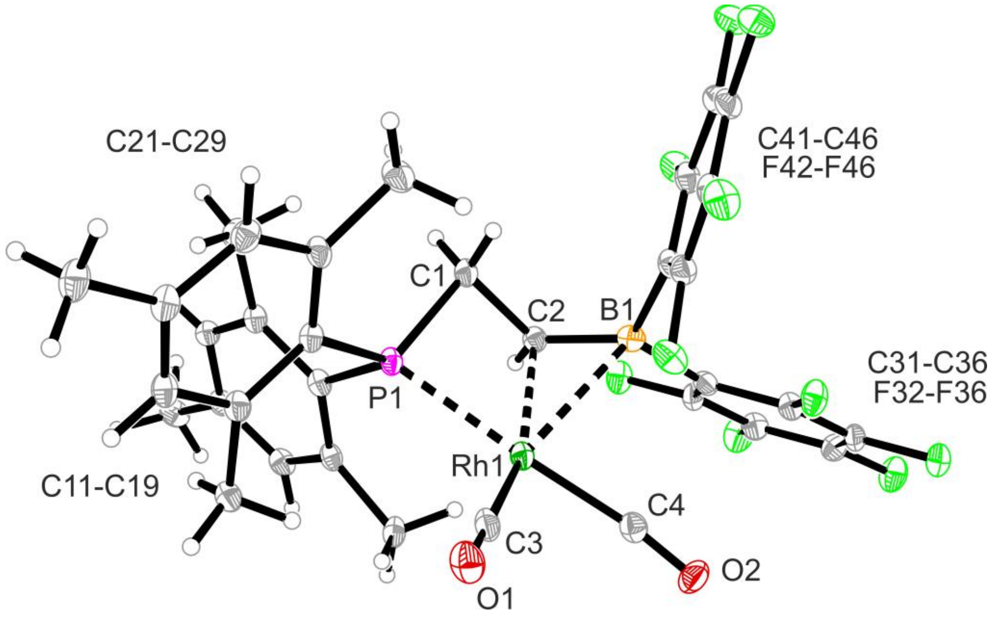

Figure S54. Crystal structure of compound **13** (thermal ellipsoids: 30% probability).

Preparation of compound 15

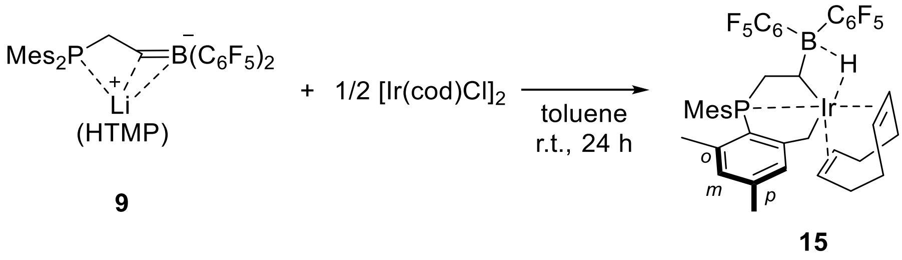

Scheme S7

1st Experiment: A mixture of compound **9** (1.1843 g, 1.5 mmol) and (1,5-cyclooctadiene)iridium(I) chloride dimer (503.8 mg, 0.75 mmol) in toluene (5 mL) was stirred for 24 hours at room temperature. Then, all volatiles were removed in *vacuo*. The obtained residue was washed with *n*-pentane (3 × 5 mL) and dried in *vacuo* to give compound **15** as a brown powder (900 mg, 0.96 mmol, 64%). The sample contains lithium chloride.

Decomposing point: 149 °C

HRMS (ESI, acetonitrile + AgTFA): *m/z* calc. for $[C_{40}H_{37}BF_{10}PIr+Ag]^+$ 1049.1240; found 1049.1257.

^{1}H NMR (600 MHz, CD_2Cl_2, 299 K): δ = [7.06, 6.95](each s, each 1H, *m*-Mes), 6.69 (s, 1H, *m*′-C=), 6.52 (s, 1H, *m*-C=), [5.91, 3.01](each m, each 1H, PCH_2), [4.23, 3.90, 3.63, 2.04](each m, each 1H, $=CH^{cod}$), [3.21/3.11, 2.30/1.61, 2.17/1.76, 1.91/1.45] (each m, each 1H, CH_2^{cod}), [2.99, 1.98](each s, each 3H, *o*-Me^{Mes}), [2.84, 2.40](each d, each $^2J_{HH}$ = 15.6 Hz, each 1H, $IrCH_2$), 2.35 (s, 3H, *p*-Me^{Mes}), 2.14 (s, 3H, *p*-$Me^{C=}$)), 1.75 (br, 1H, BCH), 1.72 (s, 3H, *o*-$Me^{C=}$), -10.4 (d, $^2J_{PH}$ = 69.1 Hz, 1H, IrH).

^{13}C{^{1}H} NMR (151 MHz, CD_2Cl_2, 299 K): δ = 155.6 (d, $^2J_{PC}$ = 36.2 Hz, *o*′-C=), [145.0 (d, $^2J_{PC}$ = 12.4 Hz), 140.4 (d, $^2J_{PC}$ = 5.4 Hz)](*o*-Mes), 141.4 (d, $^4J_{PC}$ = 1.2 Hz, *p*-Mes), 140.5 (d, $^4J_{PC}$ = 1.1 Hz, *p*-C=), 138.4 (*o*-C=), [132.0 (d, $^3J_{PC}$ = 8.3 Hz), 131.4 (d, $^3J_{PC}$ = 8.6 Hz)](*m*-Mes), 131.8 (d, $^1J_{PC}$ = 55.0 Hz, *i*-C=), 128.7 (d, $^3J_{PC}$ = 8.0 Hz, *m*-C=), 128.0 (d, $^3J_{PC}$ = 20.2 Hz, *m*′-C=), 121.0 (d, $^1J_{PC}$ = 31.7 Hz, *i*-Mes), [85.4, 83.9, 76.6 (d, $^2J_{PC}$ = 4.8 Hz), 71.0 (d, $^2J_{PC}$ = 3.3 Hz)](=CH^{cod}), 43.7 (d, $^1J_{PC}$ = 37.1 Hz, PCH_2), [38.5, 33.4 (d, $^3J_{PC}$ = 2.3 Hz), 29.7, 28.4](CH_2^{cod}), [24.7 (d, $^3J_{PC}$ = 2.9 Hz), 23.1 (d, $^3J_{PC}$ = 10.4 Hz)](o-Me^{Mes}), 21.1 (*p*-Me^{Mes}), 20.9 (*p*-$Me^{C=}$), 20.8 (d, $^3J_{PC}$ = 2.7 Hz, *o*-$Me^{C=}$), 17.7 ($IrCH_2$), 12.2 (br, BCH), [C_6F_5 not listed].

^{11}B NMR (192 MHz, CD_2Cl_2, 299 K): δ = -17.5 ($\nu_{1/2}$ ~ 300 Hz).

^{31}P NMR (243 MHz, CD_2Cl_2, 299 K): δ = -104.0 (br d, $^2J_{PH}$ ~ 70 Hz).

^{31}P{^{1}H} NMR (243 MHz, CD_2Cl_2, 299 K): δ = -104.0 (m).

^{19}F NMR (564 MHz, CD_2Cl_2, 299 K): δ = [-128.2 (br, 2F), -130.0 (br, 1F), -132.4 (br m, 1F)](*o*-C_6F_5), [-159.2, -159.5](each t, $^3J_{PC}$ = 20.3 Hz, each 1F, *p*-C_6F_5), [-163.9 (m), -164.7 (br)](each 2F, *m*-C_6F_5).

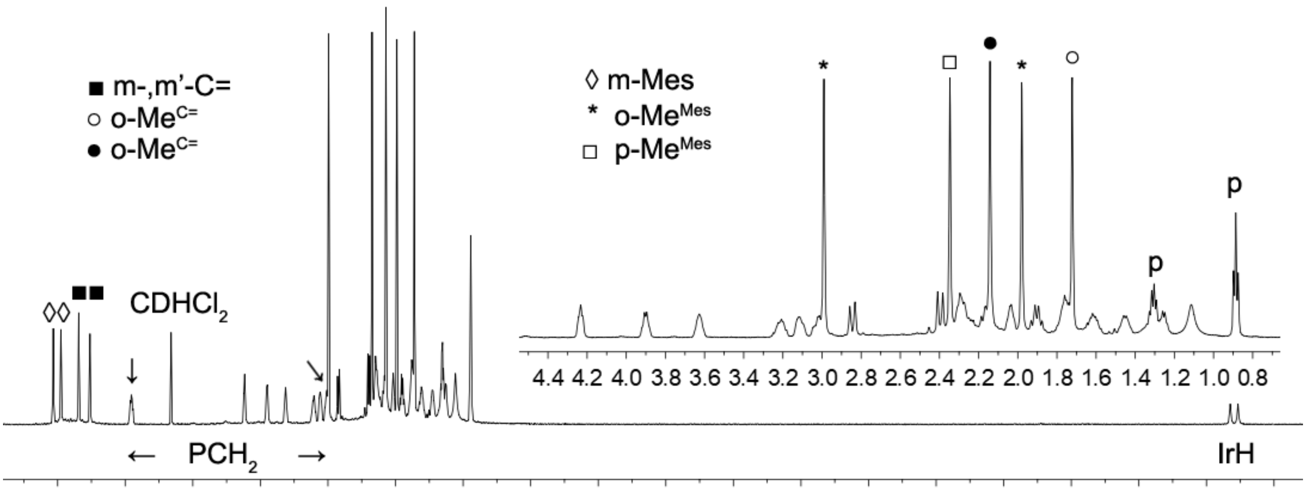

Figure S55. **^{1}H NMR** (600 MHz, CD_2Cl_2, 299K) spectrum of compound **13** [admixed with pentane (p)].

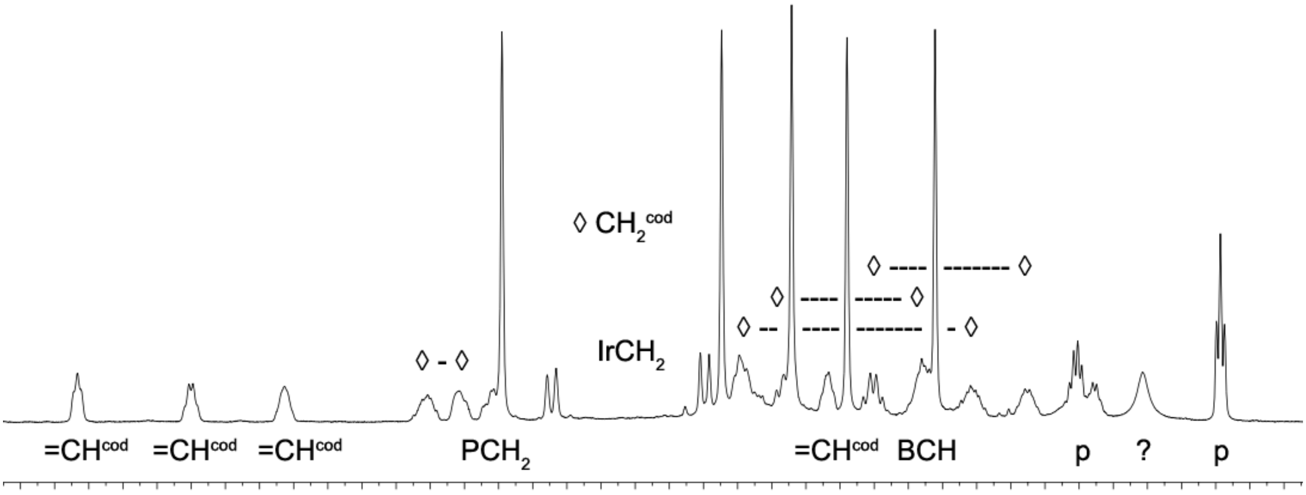

Figure S56. **^{1}H NMR** (600 MHz, CD_2Cl_2, 299K) spectrum of compound **15** [admixed with pentane (p)].

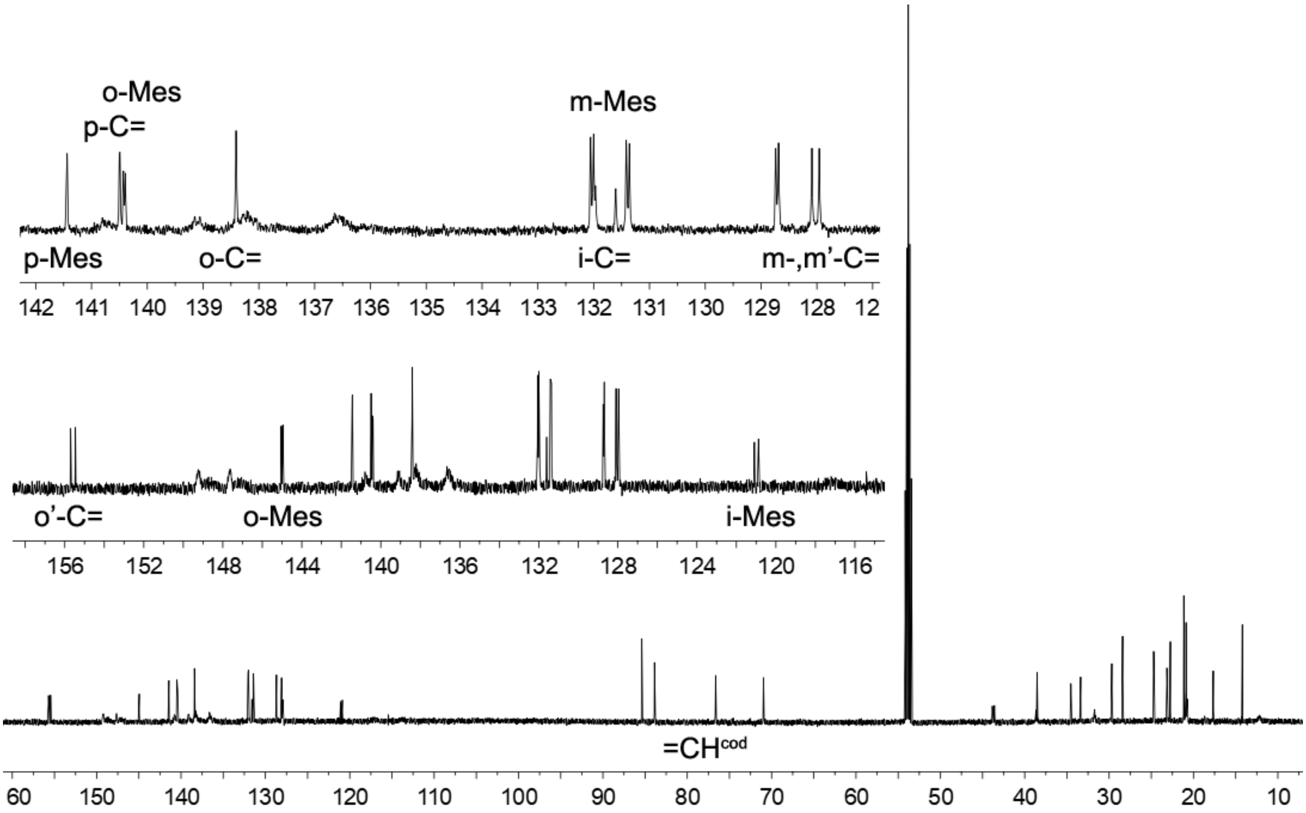

Figure S57. ^{13}**C{**^{1}**H} NMR** (151 MHz, CD_2Cl_2, 299K) spectrum of compound **15**.

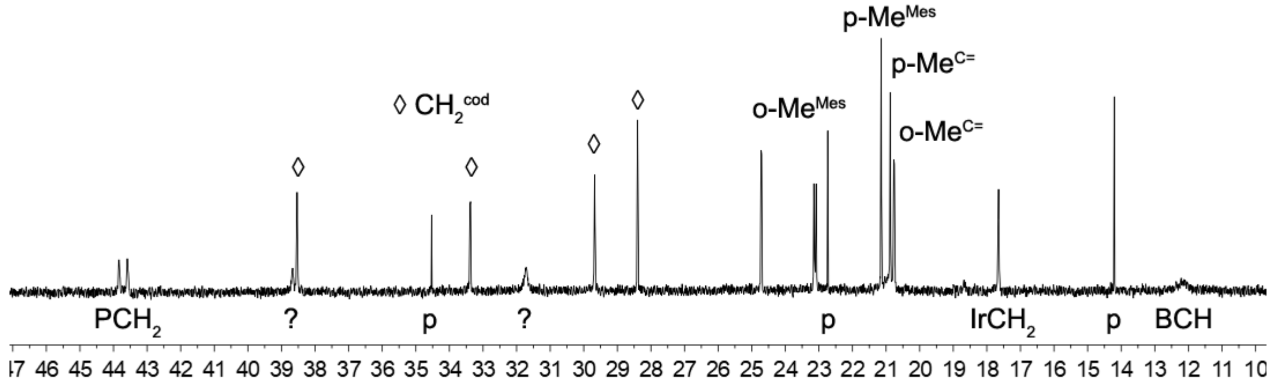

Figure S58. ^{13}**C{**^{1}**H} NMR** (151 MHz, CD_2Cl_2, 299K) spectrum of compound **15**.

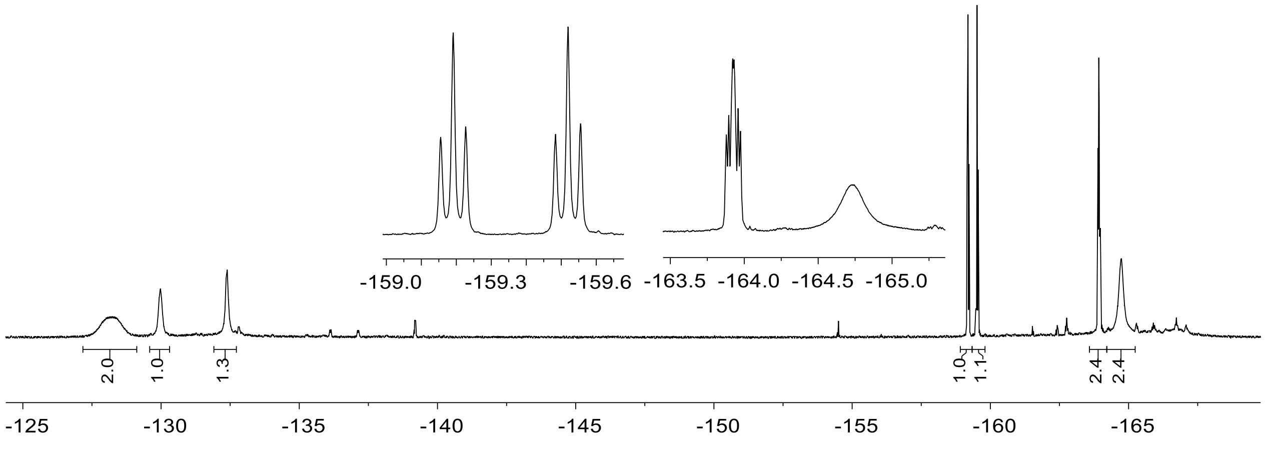

Figure S59. ^{19}**F NMR** (564 MHz, CD_2Cl_2, 299 K) spectrum of compound **15**.

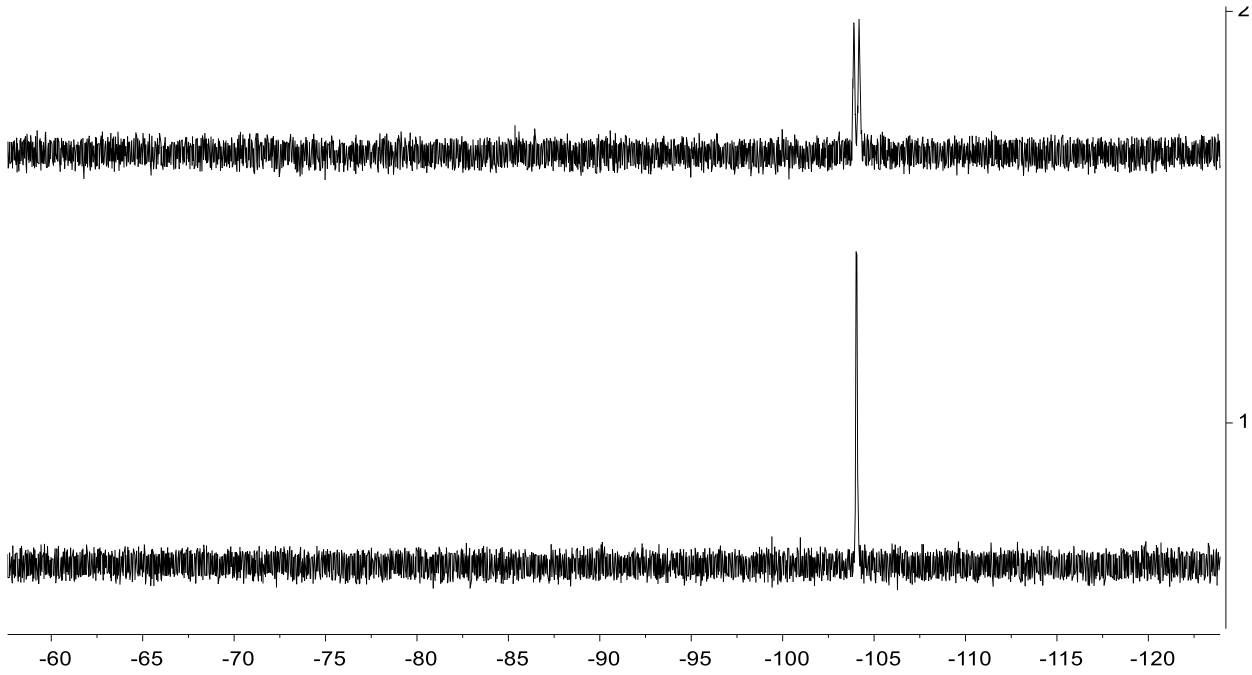

Figure S60. (1) **$^{31}P\{^1H\}$** and (2) **^{31}P NMR** (243 MHz, CD_2Cl_2, 299K) spectra of compound **15**.

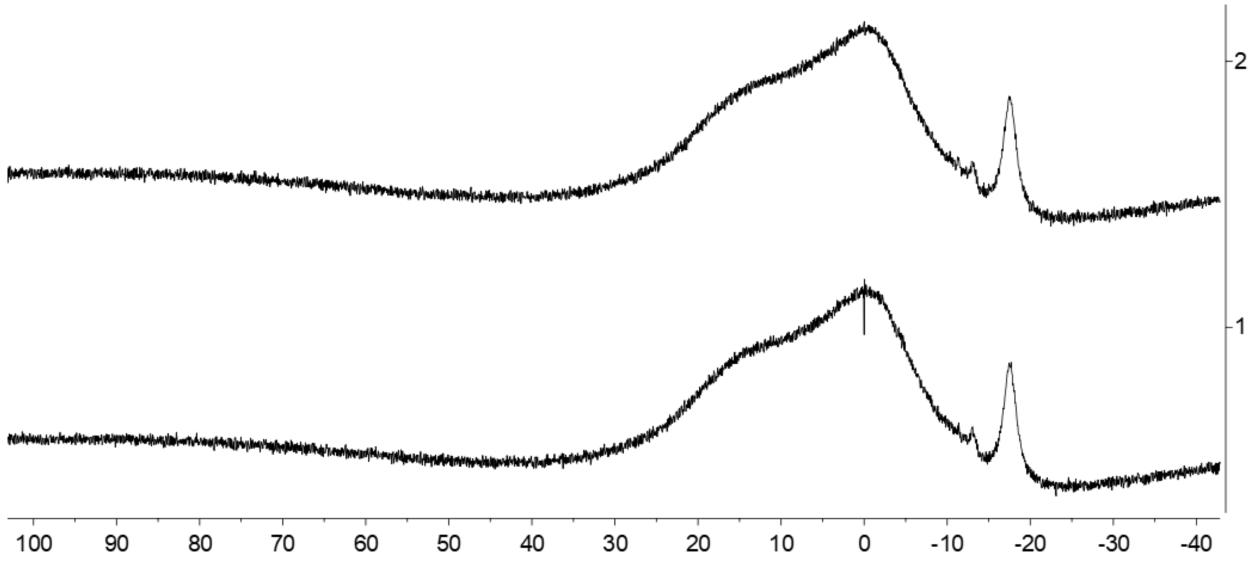

Figure S61. (1) **$^{11}B\{^1H\}$** and (2) **^{11}B NMR** (192 MHz, CD_2Cl_2, 299 K) spectra of compound **15**.

Crystals suitable for the X-ray crystal structure analysis were obtained by crystallization from a solution of compound **15** in *n*-pentane at -30 °C.

X-ray crystal structure analysis of compound 15 (erk9340): A pale yellow prism-like specimen of $C_{40}H_{37}BF_{10}IrP$, approximate dimensions 0.091 mm x 0.158 mm x 0.216 mm, was used for the X-ray crystallographic analysis. The X-ray intensity data were measured. A total of 699 frames were collected. The total exposure time was 4.85 hours. The frames were integrated with the Bruker SAINT software package using a narrow-frame algorithm. The integration of the data using a triclinic unit cell yielded a total of 87155 reflections to a maximum θ angle of 26.73° (0.79 Å resolution), of which 16436 were independent (average redundancy 5.303, completeness = 99.7%, R_{int} = 4.76%, R_{sig} = 3.77%) and 13687 (83.27%) were greater than

$2\sigma(F^2)$. The final cell constants of a = 11.0013(5) Å, b = 13.0591(5) Å, c = 28.8517(13) Å, α = 102.8050(10)°, β = 92.594(2)°, γ = 105.0650(10)°, volume = 3879.7(3) $Å^3$, are based upon the refinement of the XYZ-centroids of 9694 reflections above 20 σ(I) with 4.699° < 2θ < 55.06°. Data were corrected for absorption effects using the multi-scan method (SADABS). The ratio of minimum to maximum apparent transmission was 0.824. The calculated minimum and maximum transmission coefficients (based on crystal size) are 0.5140 and 0.7380. The structure was solved and refined using the Bruker SHELXTL Software Package, using the space group *P*-1, with Z = 4 for the formula unit, $C_{40}H_{37}BF_{10}IrP$. The final anisotropic full-matrix least-squares refinement on F^2 with 981 variables converged at R1 = 3.33%, for the observed data and wR2 = 7.26% for all data. The goodness-of-fit was 1.038. The largest peak in the final difference electron density synthesis was 4.591 $e^-/Å^3$ and the largest hole was -1.490 $e^-/Å^3$ with an RMS deviation of 0.115 $e^-/Å^3$. On the basis of the final model, the calculated density was 1.612 g/cm^3 and F(000), 1856 e^-. CCDC number: 1960306.

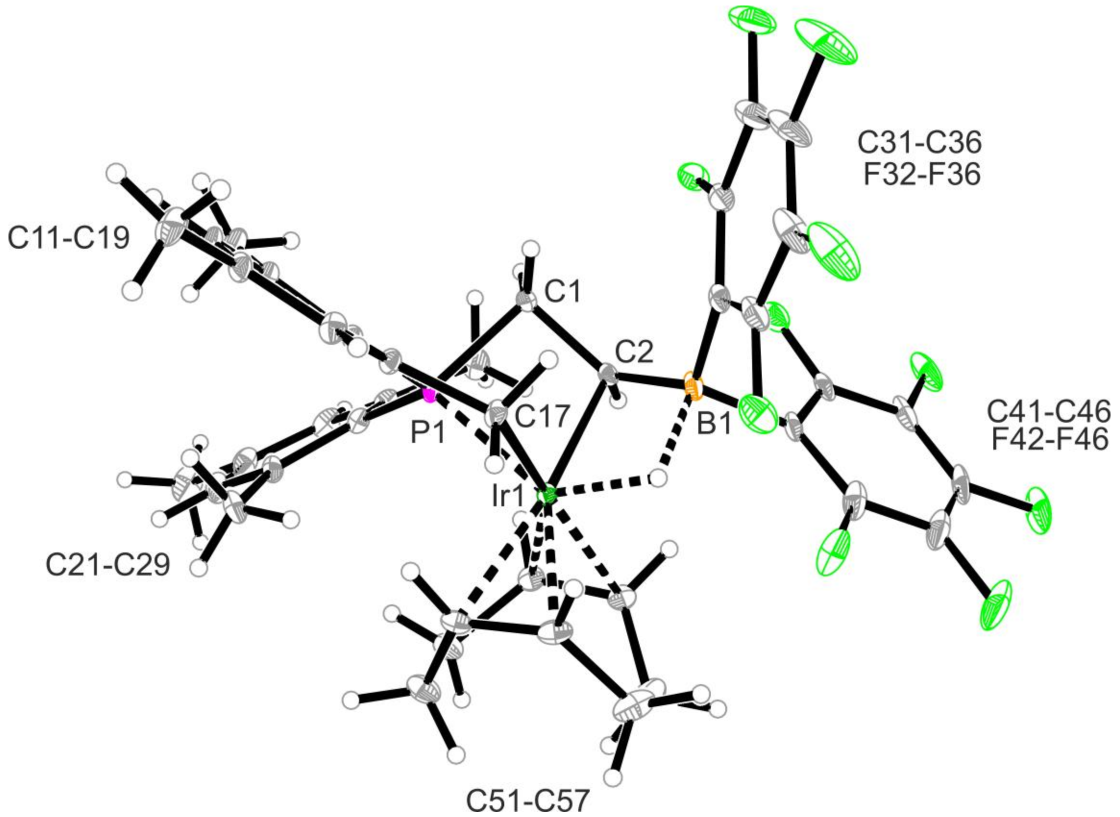

Figure S62. Crystal structure of compound **15** (thermal ellipsoids: 30% probability).

2nd Experiment: This synthetic procedure was similar to that described for the 1st experiment, but with small modifications to remove generated LiCl. A mixture of compound **9** (394.8 mg, 0.5 mmol) and (1,5-cyclooctadiene)iridium(I) chloride dimer (167.9 mg, 0.25 mmol) in toluene (40 mL) was stirred for 24 hours at room temperature, Then LiCl was filtered off by cannula filtration and all volatiles of the filtrate were removed in *vacuo*. The obtained residue was washed with *n*-pentane (3×2 mL) and dried in *vacuo* to give compound **15** as a brown powder (210.7 mg, 0.22 mmol, 44 %). The characterization data are consistent to those described in the 1st experiment.

Elemental analysis: calc. for $C_{40}H_{37}BF_{10}PIr$ (941.72 g/mol): C, 51.02; H, 3.96. Found: C, 51.32, H, 4.49.

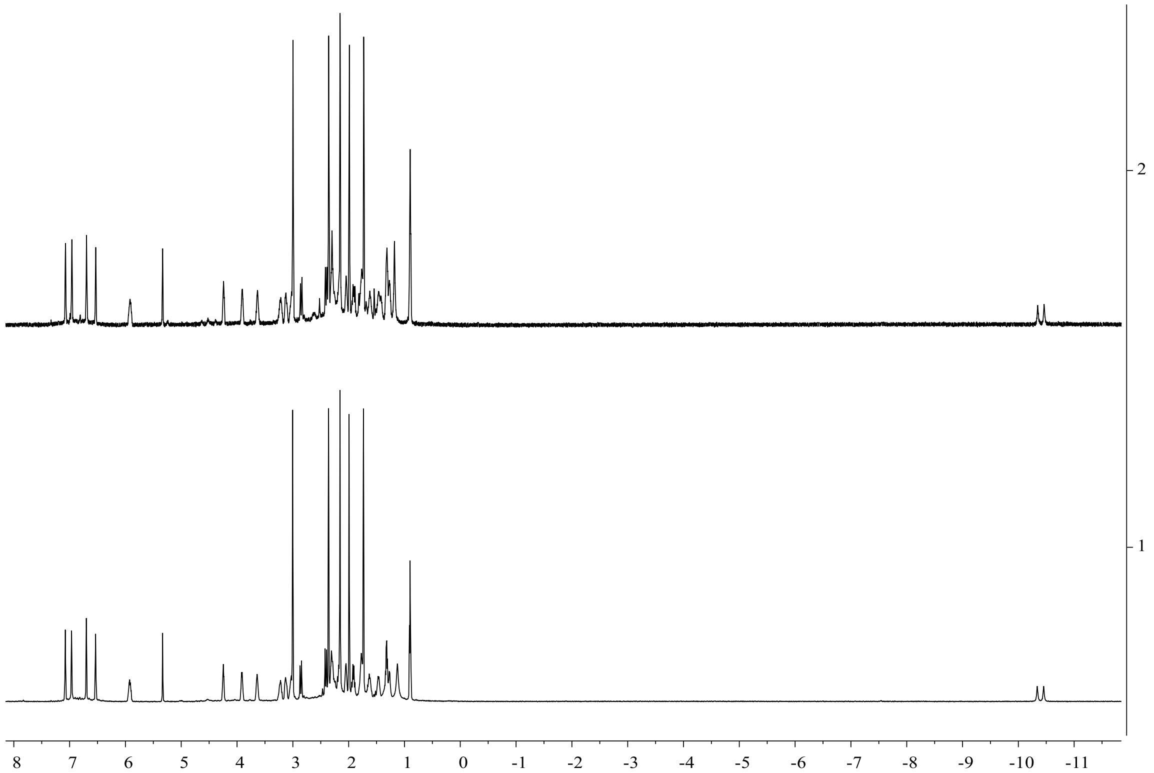

Figure S63. **^{1}H NMR** (600 MHz, CD_2Cl_2, 299K) spectra of compound **15:** (1) 1st experiment and (2) 2nd experiment.

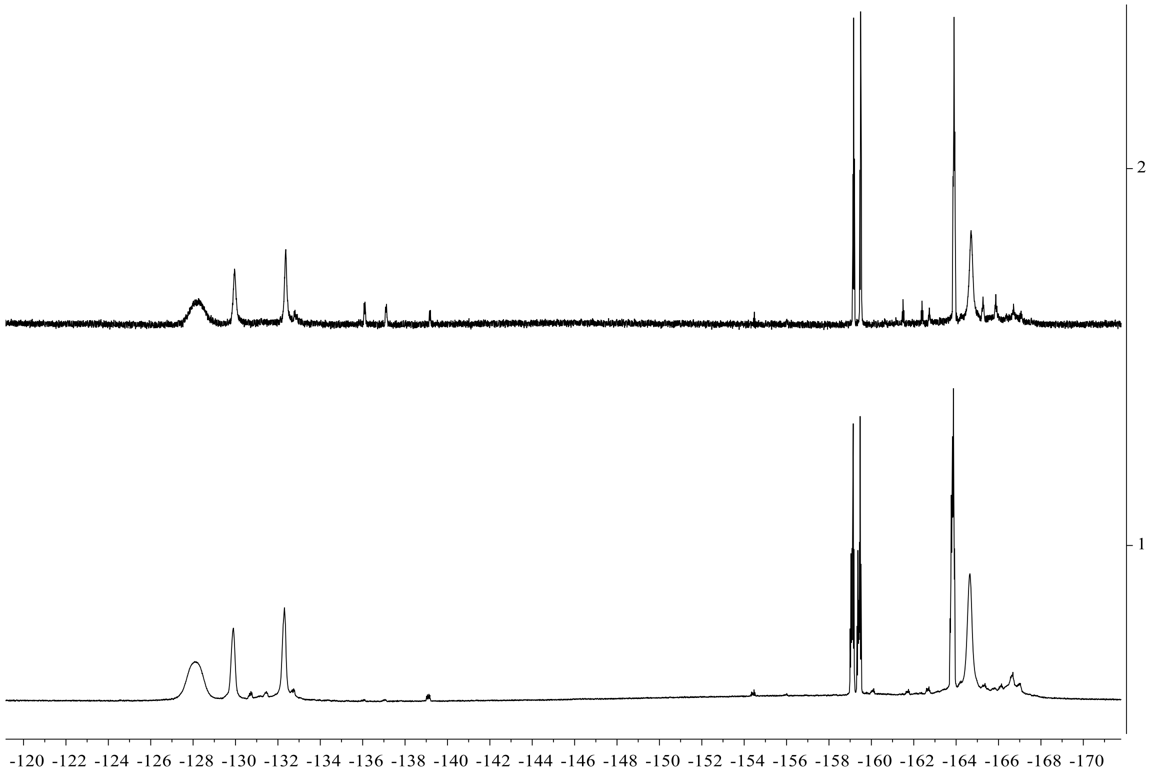

Figure S64. **^{19}F NMR** (564 MHz, CD_2Cl_2, 299 K) spectra of compound **15:** (1) 1st experiment and (2) 2nd experiment.

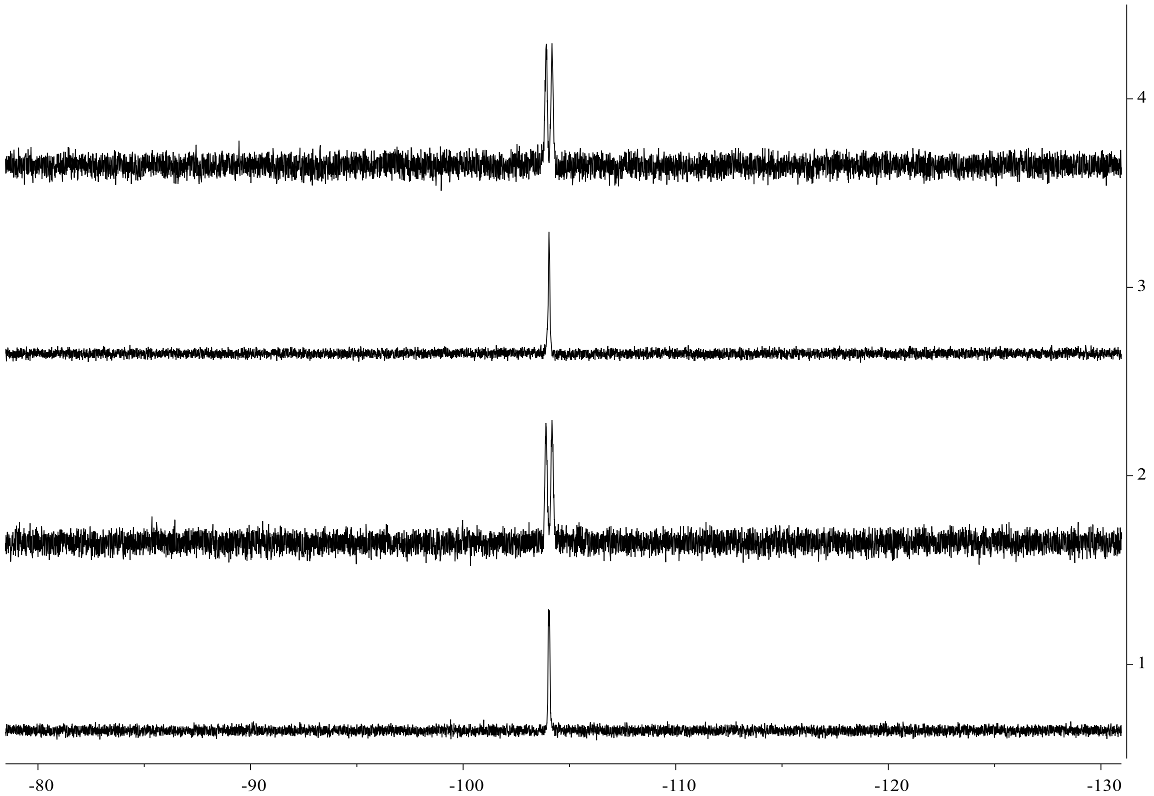

Figure S65. (1,3) ^{31}**P{^{1}H}** and (2,4) ^{31}**P NMR** (243 MHz, CD_2Cl_2, 299K) spectra of compound **15:** (1,2) 1st experiment and (3,4) 2nd experiment.

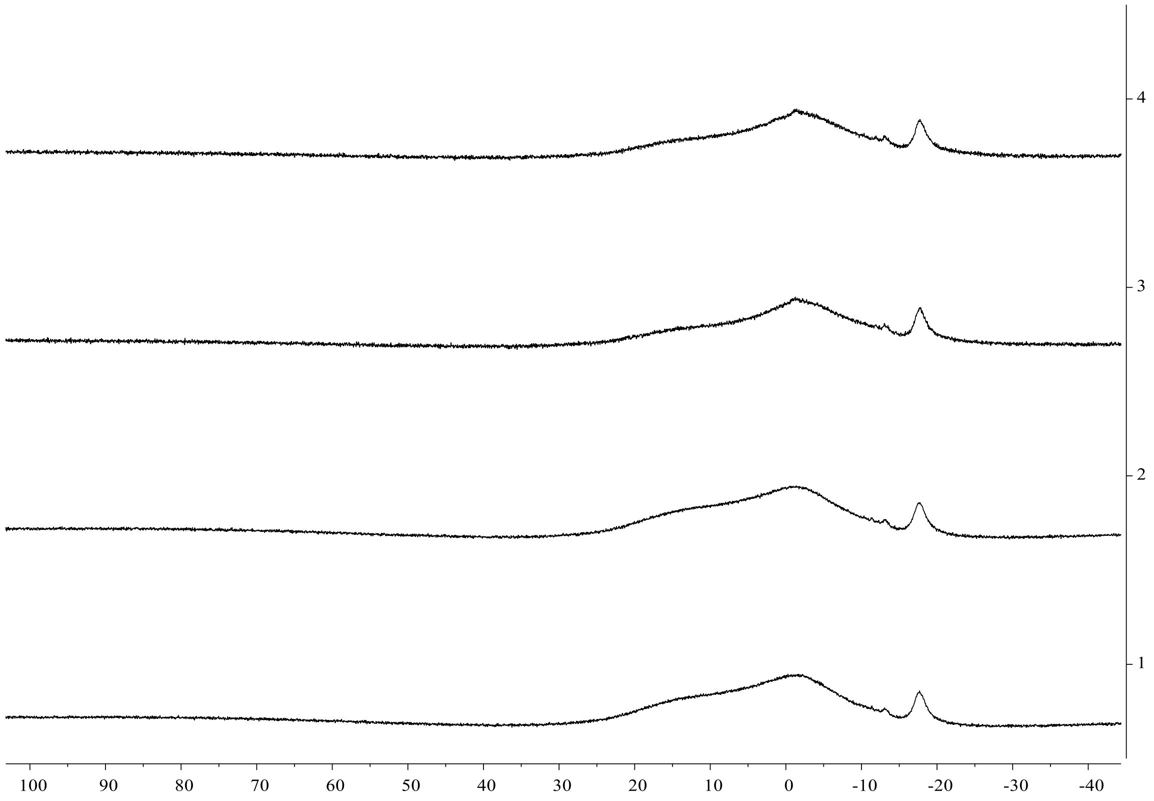

Figure S66. ^{11}**B{^{1}H}** and ^{11}**B NMR** (192 MHz, CD_2Cl_2, 299 K) spectra of compound **15:** (1,2) 1st experiment and (3,4) 2nd experiment.

Catalytic reactions: Rh complex **12** and Ir complex **15** obtained from the 1st experiment were used for the catalytic experiments.

Catalytic hydrogenation

Experiment 1: Hydrogenation of styrene in the presence of the Ir complex 15 (0.5 mol%)

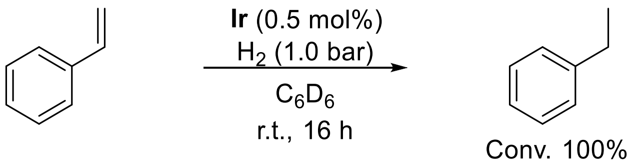

Scheme S8

In a glovebox with an argon atmosphere, a mixture of compound **13** (Ir complex) (2.4 mg, 0.0025 mmol, 0.5 mol%) and styrene (52.1 mg, 0.5 mmol) was dissolved in C_6D_6 (2.0 mL). Then the obtained solution was transferred to a Schlenk flask. After the mixture was degassed, it was stirred at room temperature for 16 hours in an H_2 atmosphere (1.0 bar). The obtained reaction mixture was characterized by ^{1}H NMR experiments: conversion of styrene was quantitative.

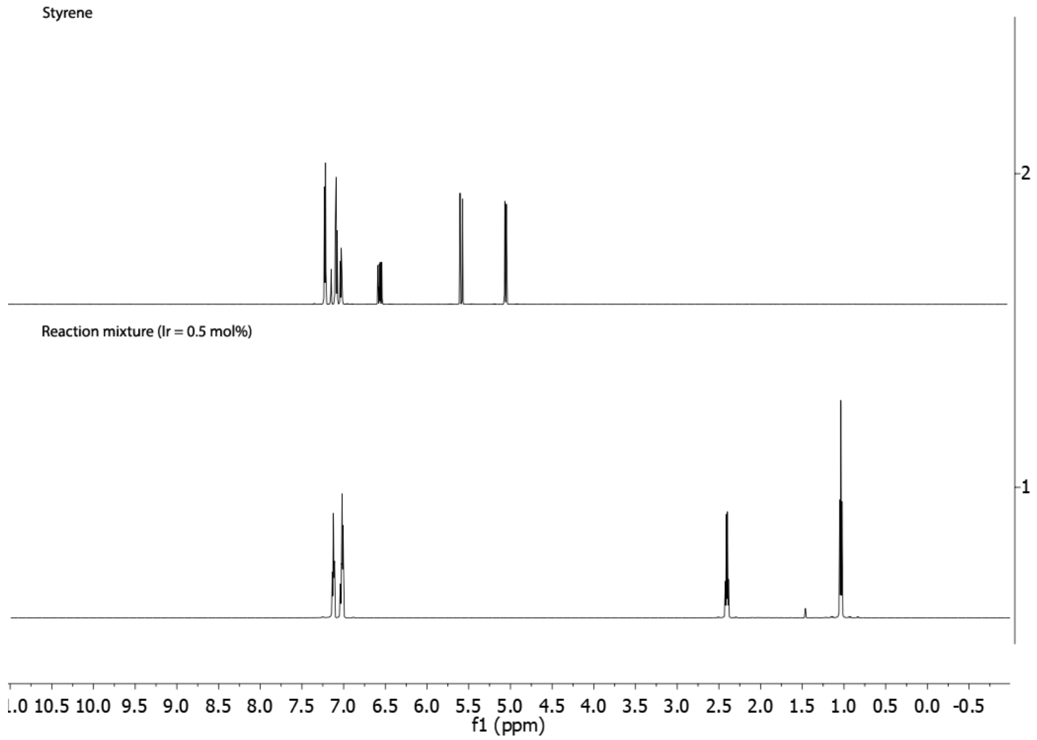

Figure S67. **^{1}H NMR** (600 MHz, C_6D_6, 299 K) spectra (1) of the obtained mixture and (2) of styrene.

Experiment 2: Hydrogenation of styrene in the presence of Ir complex 15 (0.1 mol%)

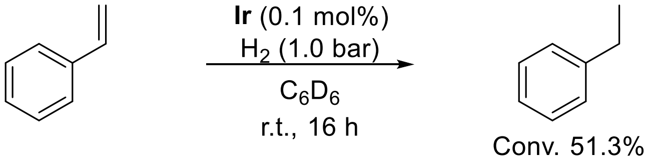

Scheme S9

In a glovebox with an argon atmosphere, a mixture of compound **15** (Ir complex) (2.4 mg, 0.0025 mmol, 0.1 mol%) and styrene (260.4 mg, 2.5 mmol) was dissolved in C_6D_6 (2.0 mL). Then the obtained solution was transferred to a Schlenk flask. After the mixture was degassed, it was stirred at room temperature for 16 hours in an H_2 atmosphere (1.0 bar). The obtained reaction mixture was characterized by ^{1}H NMR experiments: conversion of styrene was ca. 51 % (TON = 513).

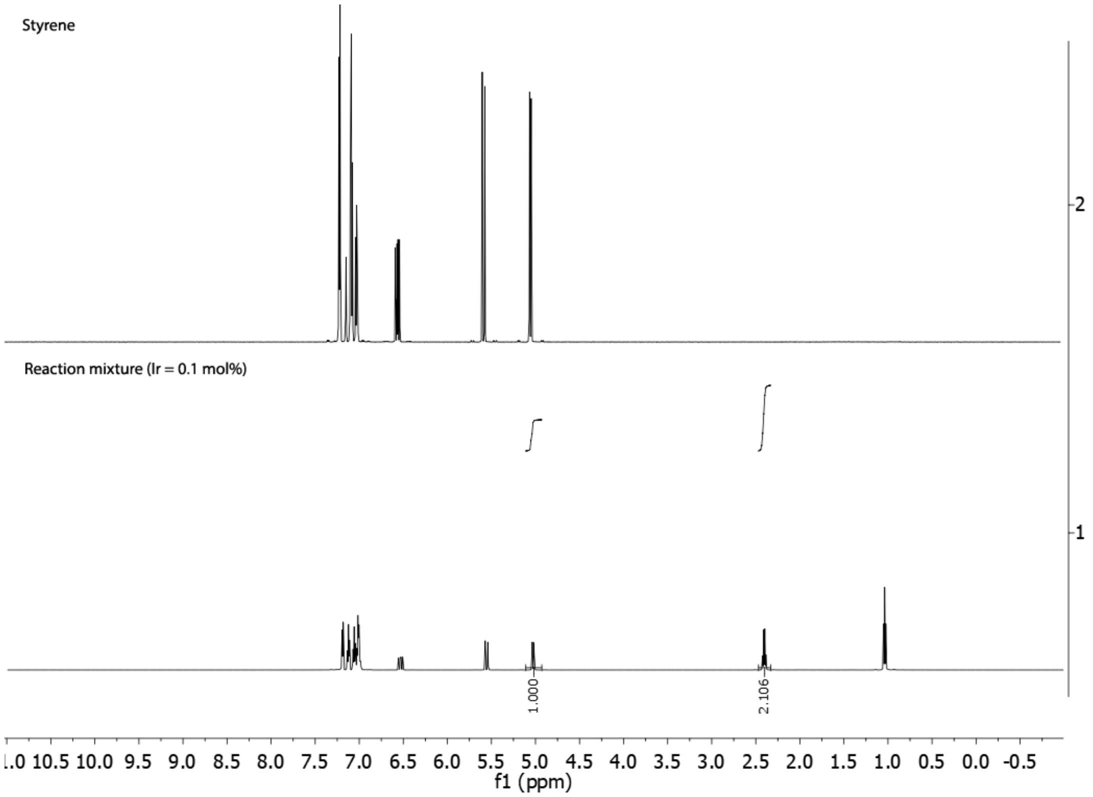

Figure S68. **^{1}H NMR** (600 MHz, C_6D_6, 299 K) spectra (1) of the obtained mixture and (2) of styrene.

Experiment 3: Hydrogenation of cyclohexene in the presence of Ir complex 15 (1.0 mol%)

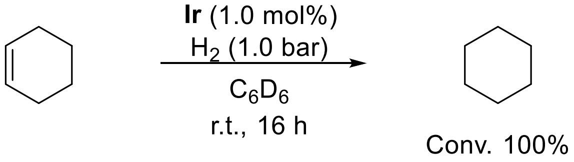

Scheme S10

In a glovebox with an argon atmosphere, a mixture of compound **15** (Ir complex) (4.8 mg, 0.0050 mmol, 1 mol%) and cyclohexene (41.1 mg, 0.5 mmol) was dissolved in C_6D_6 (2.0 mL). Then the obtained solution was transferred to a Schlenk flask. After the mixture was degassed, it was stirred at room temperature for 16 hours in an H_2 atmosphere (1.0 bar). The obtained reaction mixture was characterized by ^{1}H NMR experiments: conversion of cyclohexene was quantitative.

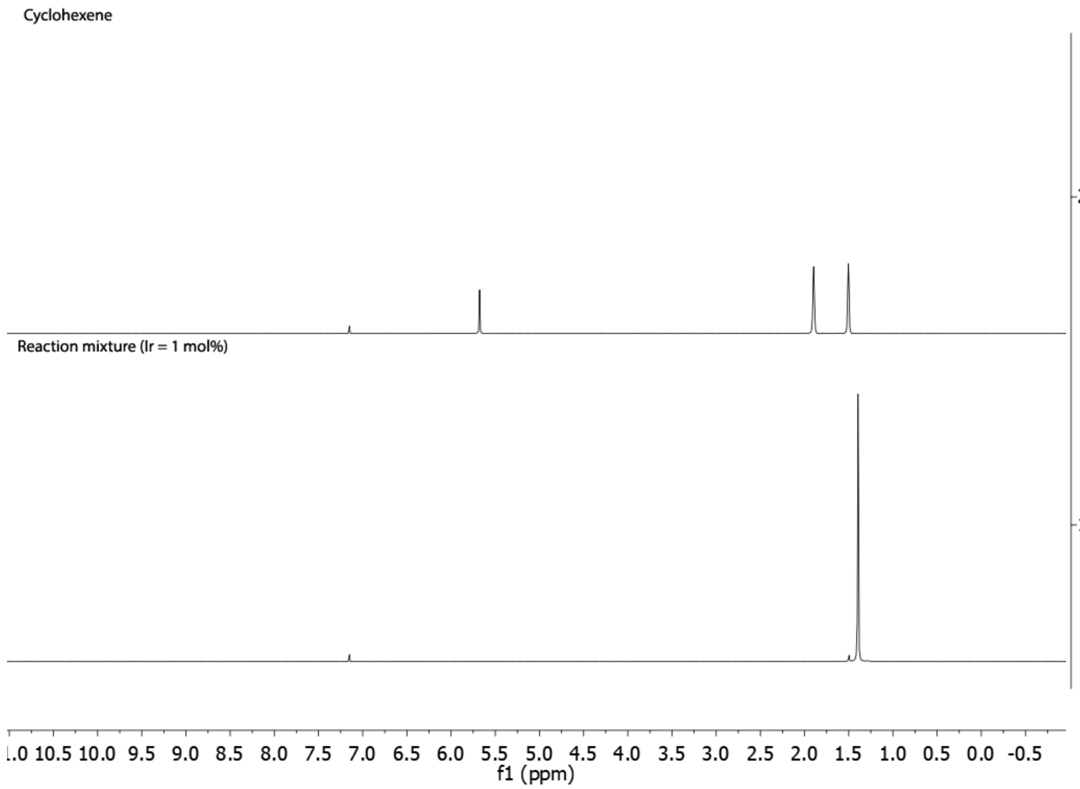

Figure S69. **^{1}H NMR** (600 MHz, C_6D_6, 299 K) spectra (1) of the obtained mixture and (2) of cyclohexene.

Experiment 4: Hydrogenation of 1-methyl-1-cyclohexene in the presence of Ir complex 15 (1.0 mol%)

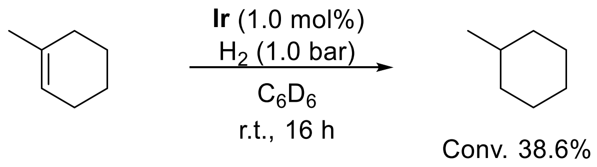

Scheme S11

In a glovebox with an argon atmosphere, a mixture of compound **15** (Ir complex) (4.8 mg, 0.0050 mmol, 1 mol%) and 1-methyl-1-cyclohexene (48.1 mg, 0.5 mmol) was dissolved in C_6D_6 (2.0 mL). Then the obtained solution was transferred to a Schlenk flask. After the mixture was degassed, it was stirred at room temperature for 16 hours in an H_2 atmosphere (1.0 bar). Mesitylene (13.6 mg, 0.113 mmol) was added to the obtained reaction mixture as an internal standard. The mixture was characterized by ^{1}H NMR experiments: conversion of 1-methyl-1-cyclohexene was ca. 38.6 mol% (TON = 39)

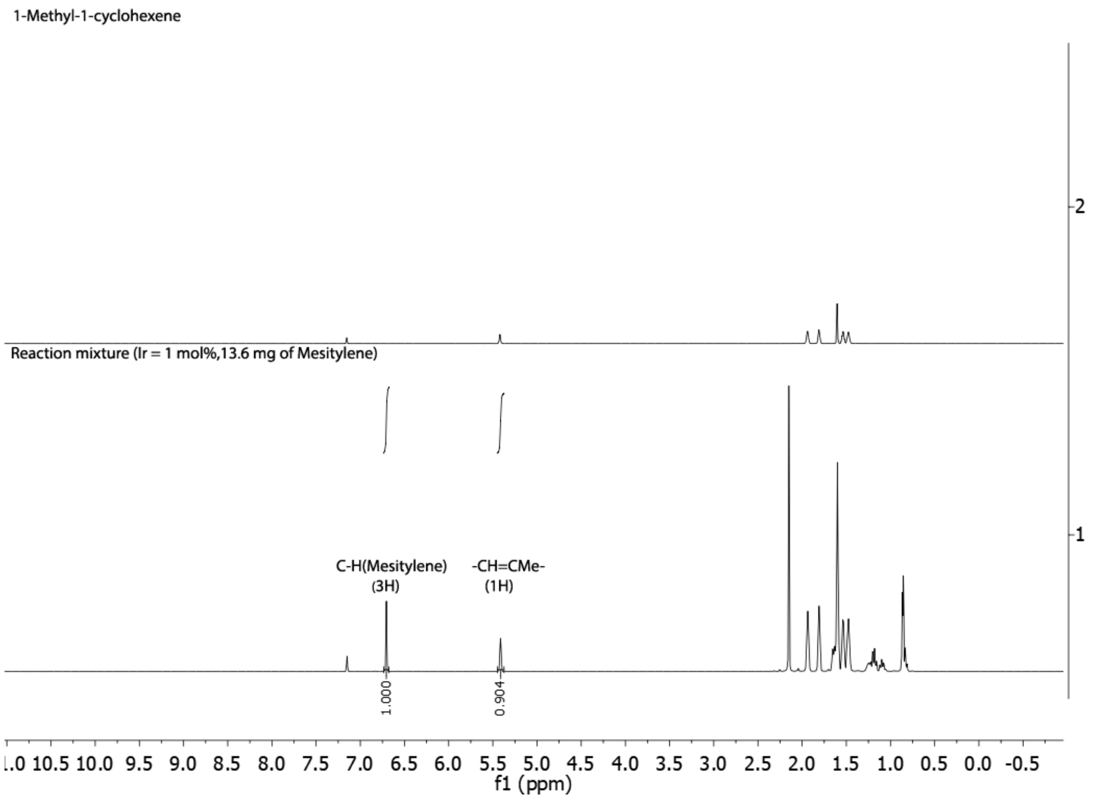

Figure S70. ^{1}H NMR (600 MHz, C_6D_6, 299 K) spectra (1) of the obtained mixture and (2) of 1-methyl-1-cyclohexene.

Experiment 5: Hydrogenation of phenylacetylene in the presence of Ir complex 15 (1.0 mol%)

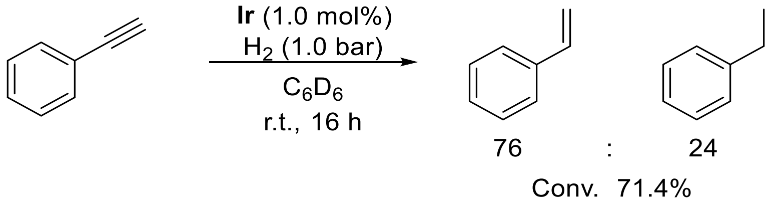

Scheme S12

In a glovebox with an argon atmosphere, a mixture of compound **15** (Ir complex) (4.7 mg, 0.0050 mmol, 1 mol%) and phenylacetylene (51.1 mg, 0.5 mmol) was dissolved in C_6D_6 (2.0 mL). Then the obtained solution was transferred to a Schlenk flask. After the mixture was degassed, it was stirred at room temperature for 16 hours in an H_2 atmosphere (1.0 bar). Then, the obtained reaction mixture was characterized by ^{1}H NMR spectroscopy: conversion of phenylacetylene was ca. 71 % (TON = 71). The ratio of styrene to ethylbenzene was ca. 76 : 24.

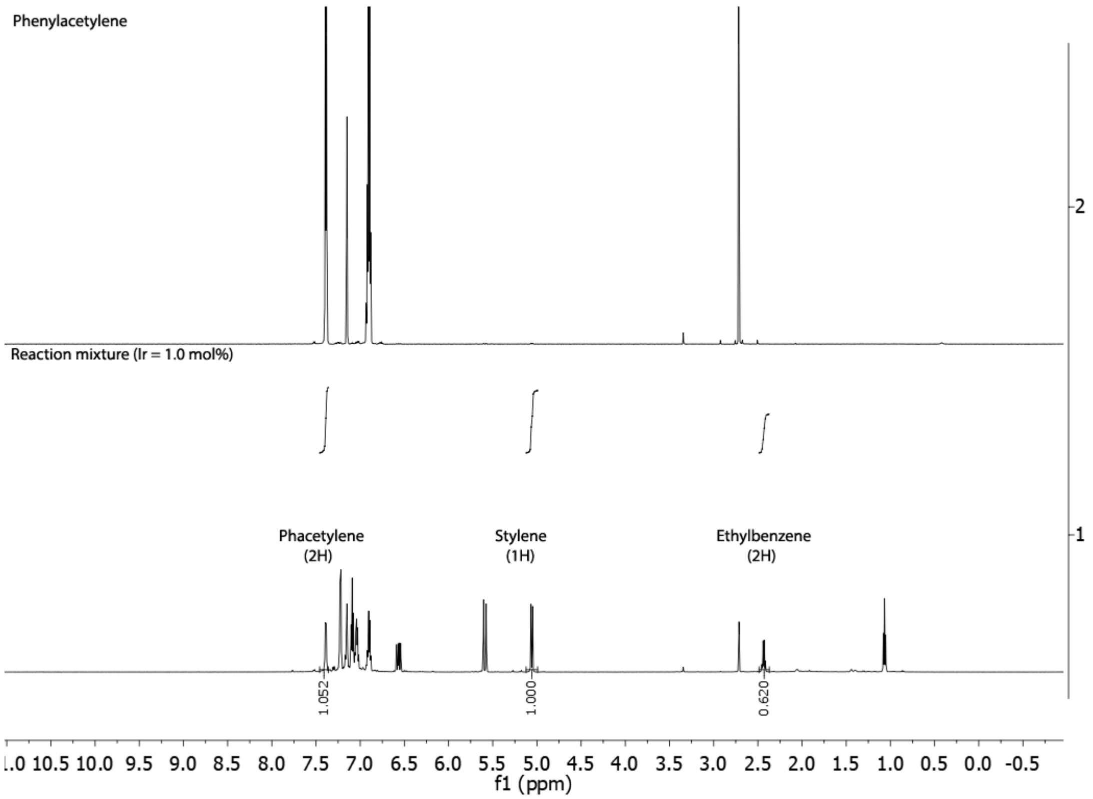

Figure S71. **^{1}H NMR** (600 MHz, C_6D_6, 299 K) spectra (1) of the obtained mixture and (2) of phenylacetylene.

Experiment 6: Hydrogenation of styrene in the presence of Rh complex 12 (0.5 mol%)

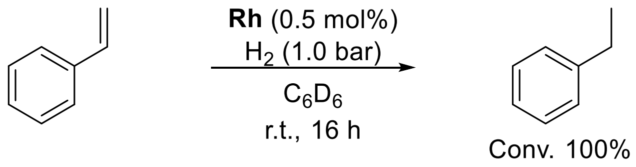

Scheme S13

In a glovebox with an argon atmosphere, a mixture of Rh complex **12** (2.1 mg, 0.0025 mmol, 0.5 mol%) and styrene (52.1 mg, 0.5 mmol) was dissolved in C_6D_6 (2.0 mL). Then the obtained solution was transferred to a Schlenk flask. After the mixture was degassed, it was stirred at room temperature for 16 hours in an H_2 atmosphere (1.0 bar). The obtained reaction mixture was characterized by ^{1}H NMR spectroscopy: conversion of styrene was quantitative.

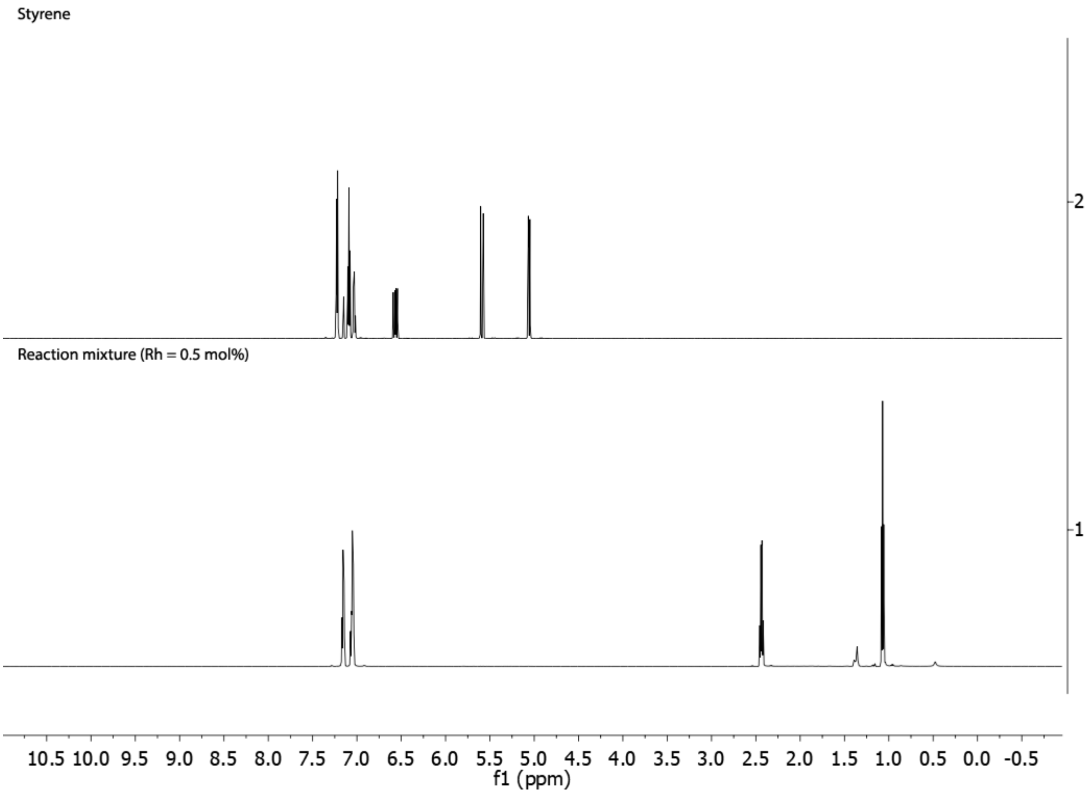

Figure S72. **^{1}H NMR** (600 MHz, C_6D_6, 299 K) spectra (1) of the obtained mixture and (2) of styrene.

Experiment 7: Hydrogenation of styrene in the presence of Rh complex 12 (0.1 mol%)

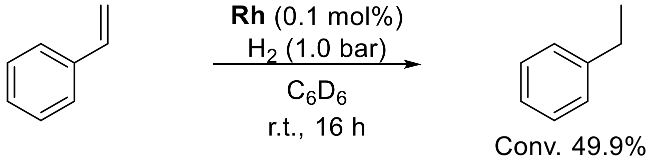

Scheme S14

In a glovebox with an argon atmosphere, a mixture of Rh complex **12** (2.1 mg, 0.0025 mmol, 0.1 mol%) and styrene (250.4 mg, 2.5 mmol) was dissolved in C_6D_6 (2.0 mL). Then the obtained solution was transferred to a Schlenk flask. After the mixture was degassed, it was stirred at room temperature for 16 hours in an H_2 atmosphere (1.0 bar). The obtained reaction mixture was characterized by ^{1}H NMR spectroscopy: conversion of styrene was ca. 50 % (TON = 500).

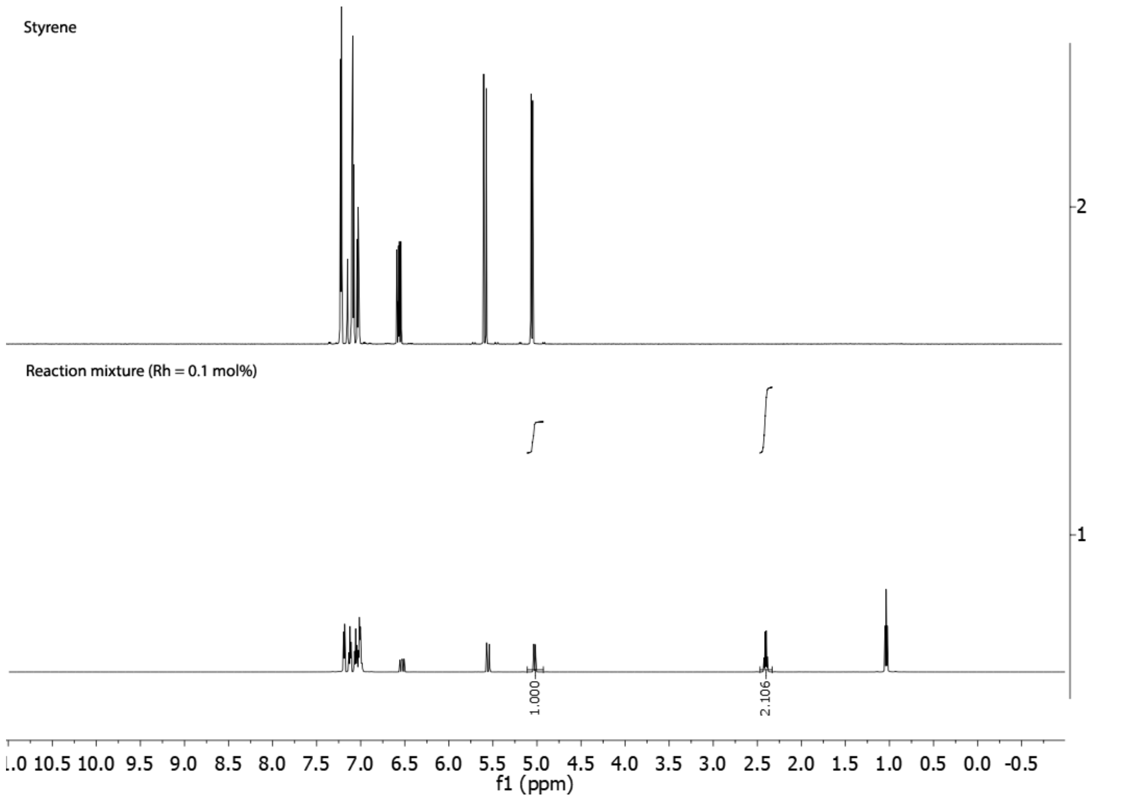

Figure S73. ^{1}H NMR (600 MHz, C_6D_6, 299 K) spectra (1) of the obtained mixture and (2) of styrene.

Experiment 8: Hydrogenation of styrene in the presence of Wilkinson catalyst (0.1 mol%)

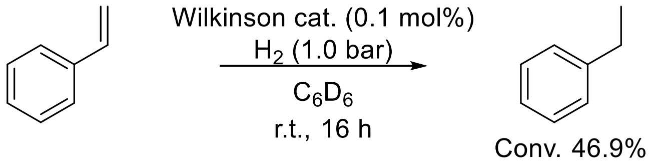

Scheme S15

In a glovebox with an argon atmosphere, a mixture of Wilkinson catalyst (2.3 mg, 0.0025 mmol, 0.1 mol%) and styrene (260.5 mg, 2.5 mmol) was dissolved in C_6D_6 (2.0 mL). Then the obtained solution was transferred to a Schlenk flask. After the mixture was degassed, it was stirred at room temperature for 16 hours in an H_2 atmosphere (1.0 bar). The obtained reaction mixture was characterized by ^{1}H NMR experiments: conversion of styrene was ca. 47 % (TON = 470).

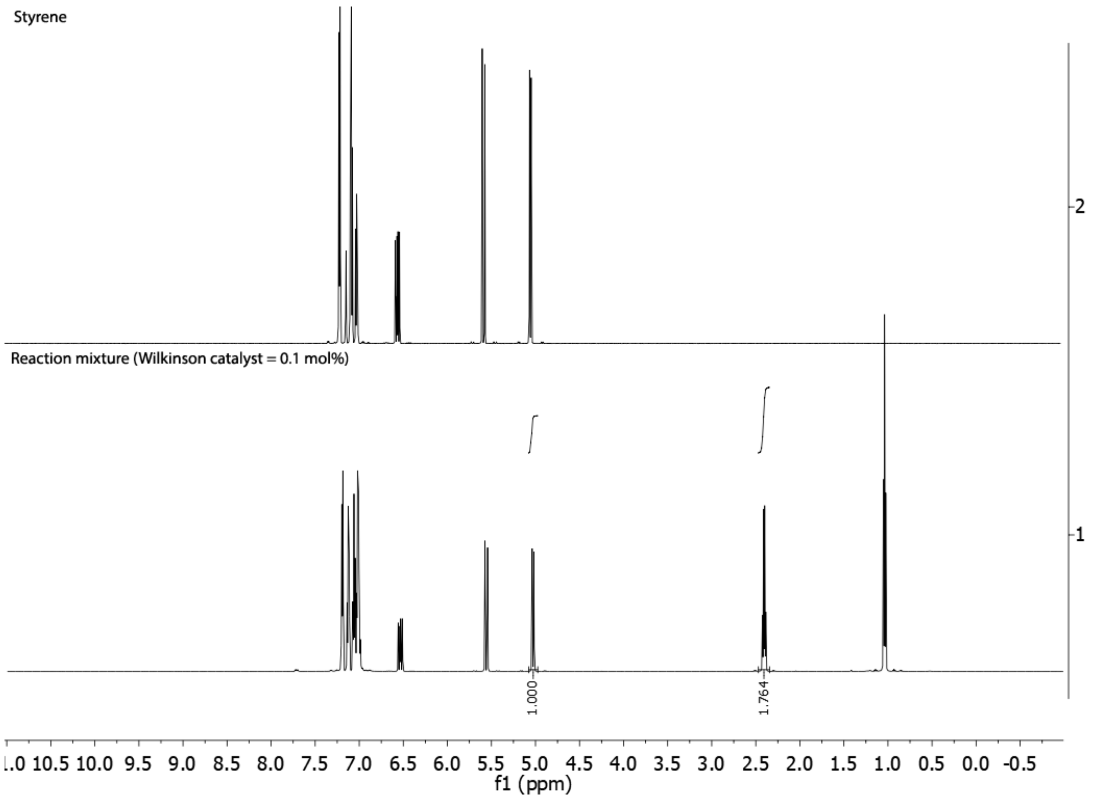

Figure S74. **^{1}H NMR** (600 MHz, C_6D_6, 299 K) spectra (1) of the obtained mixture and (2) of styrene.

Experiment 9: Hydrogenation of cyclohexene in the presence of Rh complex 12 (0.1 mol%)

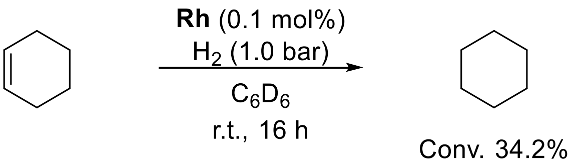

Scheme S16

In a glovebox with an argon atmosphere, a mixture of compound **12** (Rh complex) (2.1 mg, 0.0025 mmol, 0.1 mol%) and cyclohexene (205.4 mg, 2.5 mmol) was dissolved in C_6D_6 (2.0 mL). Then the obtained solution was transferred to a Schlenk flask. After the mixture was degassed, it was stirred at room temperature for 16 hours in an H_2 atmosphere (1.0 bar). The obtained reaction mixture was characterized by ^{1}H NMR spectroscopy: conversion of cyclohexene was ca. 34 % (TON = 340).

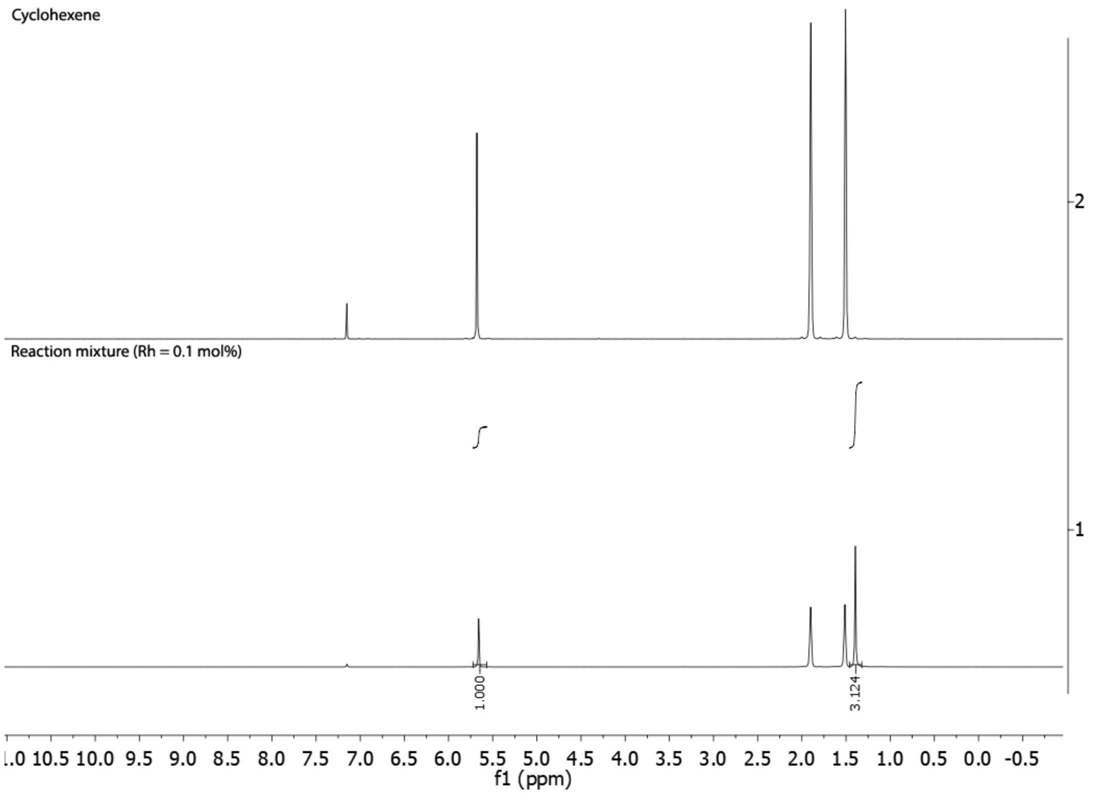

Figure S75. ^{1}H NMR (600 MHz, C_6D_6, 299 K) spectra (1) of the obtained mixture and (2) of cyclohexene.

Polymerization of arylacetylenes catalyzed by Rh complex 12

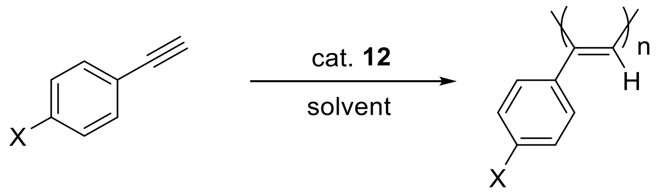

Scheme S17

Table S1 Summary of the polymerization results.

entry	X	[Rh] (mol%)	solvent	yield (%)	soluble fraction (%)	PD	M_w
1	MeO	2	ether	86	Ca. 70	2.98	340285
2	H	2	ether	99	Ca. 90	2.69	644488
3	F	2	ether	97	Ca. 95	2.84	1262340
4	H	0.1	benzene	99	5	3.20	207837
5	H	0.05	C_6D_6	96	trace	2.73	189188
6	H	0.025	benzene	45	trace	3.03	100899
7	H	0.001	benzene	28	trace	3.20	128114
8	H	0.1	THF	99	7	3.76	186128
9	F	0.1	benzene	96	73	3.89	327917
10	MeO	0.1	C_6D_6	96	29	3.89	77656

Entry 1: Polymerization of p-methoxyphenylacetylene in the presence of Rh complex 12 (2 mol%)

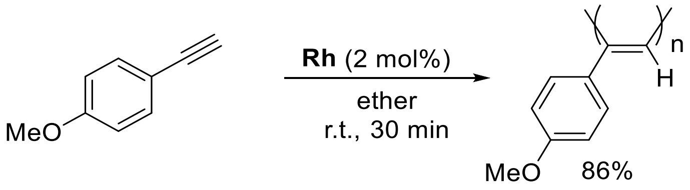

Scheme S18

Under an Argon atmosphere, a solution of p-methoxyphenylacetylene (99 mg, 0.75 mmol) in ether (2 mL) was added to a suspension of compound **12** (12.6 mg, 0.015 mmol) in ether (3 mL) at room temperature. After stirring for 30 min, acetic acid (1 mL) was added to the resulting reaction mixture and stirred for 10 min at room temperature. Subsequently, the resulting reaction mixture was poured to methanol (50 mL) to give a yellow suspension. Then the precipitates were filtrated, washed with methanol and dried in vacuo at room temperature for 24 h giving yellow solids (85 mg, 86%). The obtained yellow solid (10 mg) was then dissolved in d_8-tetrahydrofuran (1 mL), the soluble part (percentage shown in Table S1) was characterized by NMR experiments.

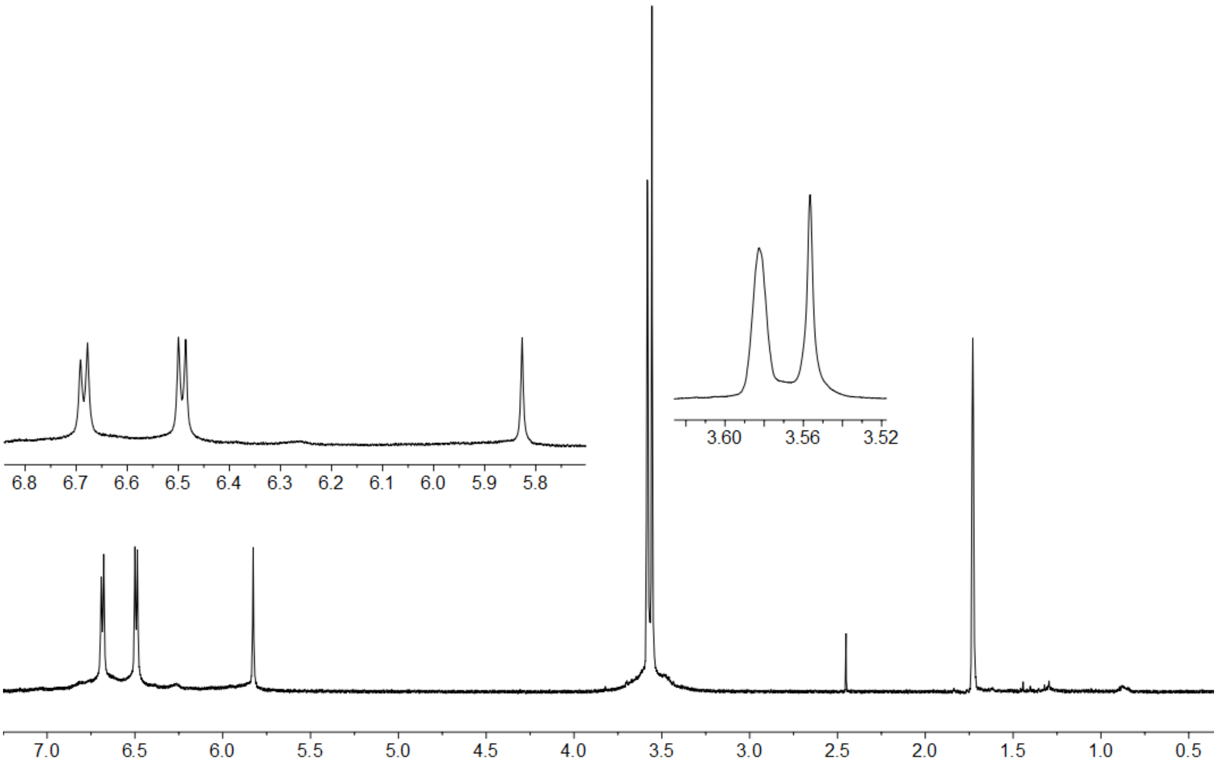

Figure S76. ^{1}H NMR (600 MHz, d_8-tetrahydrofuran, 299K) spectrum of poly(p-methoxyphenylacetylene).

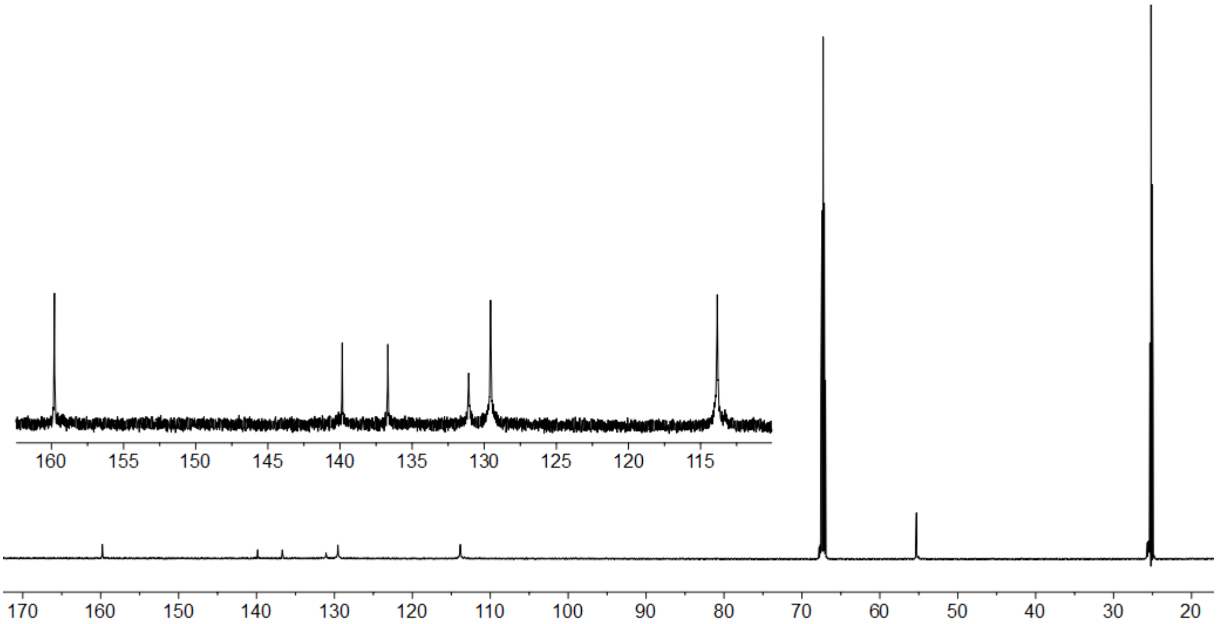

Figure S77. $^{13}C\{^{1}H\}$ NMR (151 MHz, d_8-tetrahydrofuran, 299K) spectrum of poly(p-methoxyphenylacetylene).

Entry 2: Polymerization of phenylacetylene in the presence of Rh complex 12 (2 mol%)

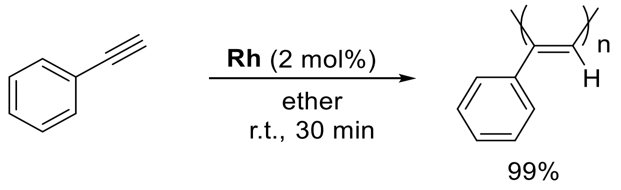

Scheme S19

Under an Argon atmosphere, a solution of phenylacetylene (76 mg, 0.75 mmol) in ether (2 mL) was added to a suspension of compound **12** (12.6 mg, 0.015 mmol) in ether (3 mL) at room temperature. After stirring for 30 min, acetic acid (1 mL) was added to the resulting reaction mixture and stirred for 10 min at room temperature. Subsequently, the resulting reaction mixture was poured to methanol (50 mL) to give a yellow suspension. The precipitates were then filtrated, washed with methanol and dried in vacuo at room temperature for 24 h giving yellow solids (75 mg, 99%). The obtained yellow solid (10 mg) was then dissolved in d_8-tetrahydrofuran (1 mL), the soluble part (percentage shown in Table S1) was characterized by NMR experiments.

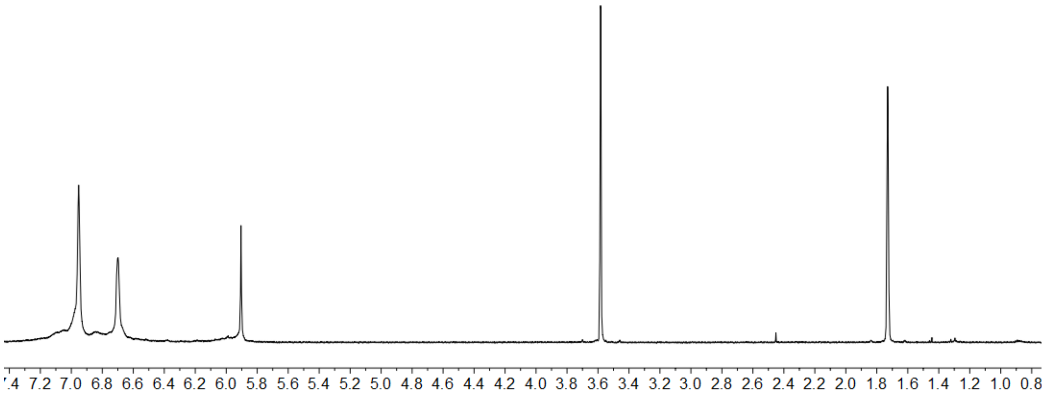

Figure S78. ^{1}H NMR (600 MHz, d_8-tetrahydrofuran, 299K) spectrum of poly(phenylacetylene).

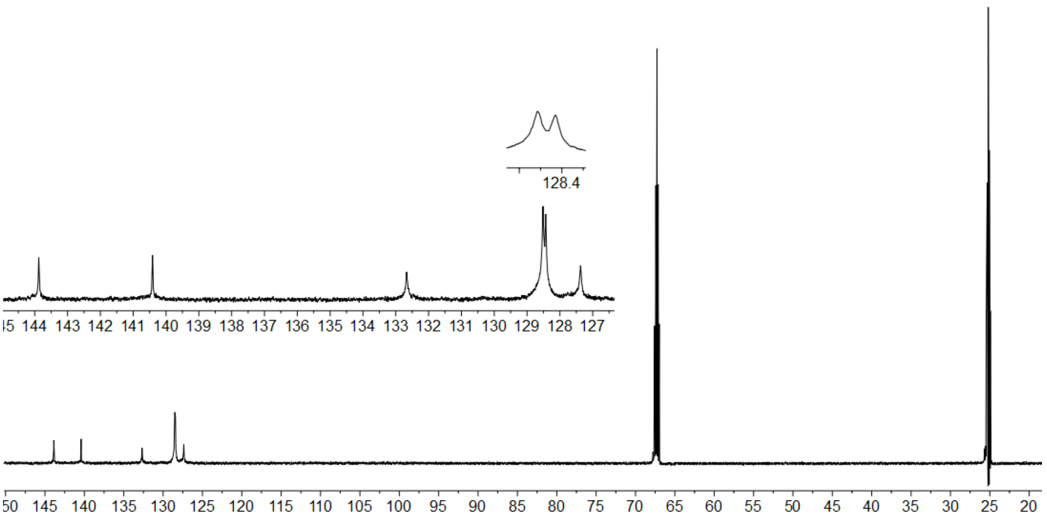

Figure S79. $^{13}C\{^1H\}$ NMR (151 MHz, d_8-tetrahydrofuran, 299K) spectrum of poly(phenylacetylene).

Entry 3: Polymerization of p-fluorophenylacetylene in the presence of Rh complex 12 (2 mol%)

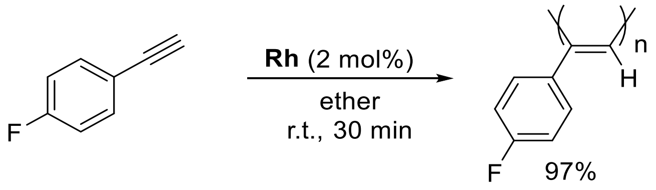

Scheme S20

Under an Argon atmosphere, a solution of p-fluorophenylacetylene (90 mg, 0.75 mmol) in ether (2 mL) was added to a suspension of compound **12** (12.6 mg, 0.015 mmol) in ether (3 mL) at room temperature. After stirring for 30 min, acetic acid (1 mL) was added to the resulting reaction mixture and stirred for 10 min at room temperature. Subsequently, the resulting reaction mixture was poured to methanol (50 mL) to give a yellow suspension. The precipitates were then filtrated, washed with methanol and dried in vacuo at room temperature for 24 h giving yellow solids (87 mg, 97%). The obtained yellow solid (10 mg) was then dissolved in d_8-tetrahydrofuran (1 mL), the soluble part (percentage shown in Table S1) was characterized by NMR experiments.

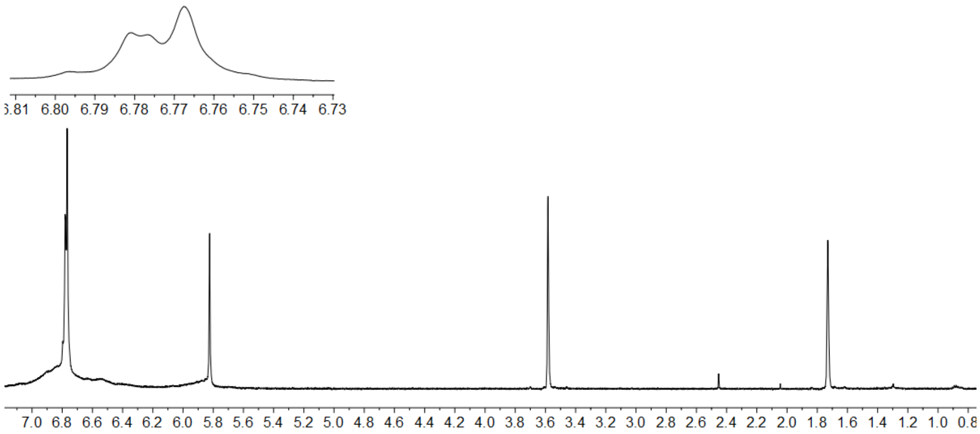

Figure S80. ^{1}H NMR (600 MHz, d_8-tetrahydrofuran, 299K) spectrum of poly(p-fluorophenylacetylene).

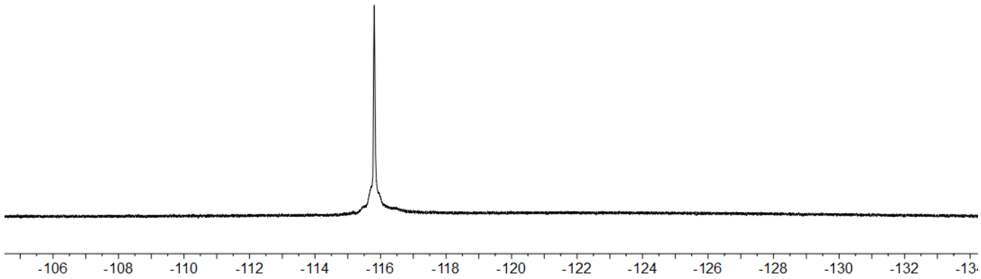

Figure S81. ^{19}F NMR (564 MHz, d_8-tetrahydrofuran, 299K) spectrum of poly(p-fluorophenylacetylene).

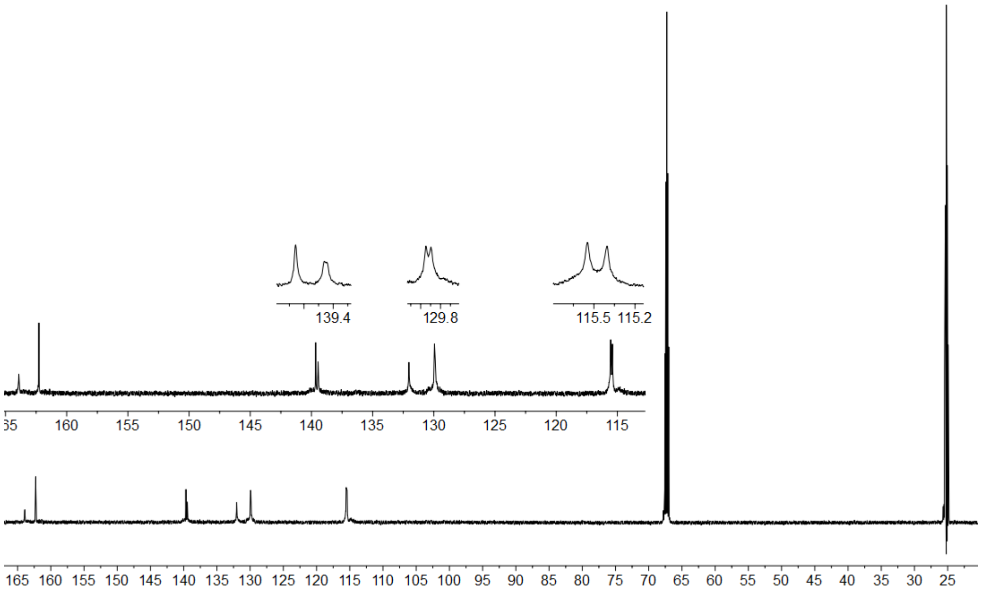

Figure S82. $^{13}C\{^1H\}$ NMR (151 MHz, d_8-tetrahydrofuran, 299K) spectrum of poly(p-fluorophenylacetylene).

Entry 4: Polymerization of phenylacetylene in the presence of Rh complex 12 (0.1 mol%)

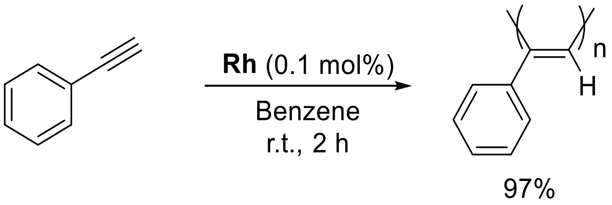

Scheme S21

In a glovebox with an argon atmosphere, compound **12** (Rh complex) (2.1 mg, 0.0025 mmol, 0.1 mol%) was dissolved in benzene (4 mL). Then, a solution of phenylacetylene (255.3 mg, 2.5 mmol) in benzene (6 mL) was gradually added. After addition, it was stirred at room temperature for 2 hours. The obtained reaction mixture was washed by methanol (10 mL, 3 times) and then dried in vacuo at 50 °C to give an orange solid (246.7 mg, 97%).

^{1}H NMR (600 MHz, CD_2Cl_2, 299 K): δ = 6.97 (m, 3H, *m,p*-Ph), 6.67 (m, 2H, *o*-Ph), 5.84 (br, 1H, =CH).

$^{13}C\{^1H\}$ NMR (151 MHz, CD_2Cl_2, 299 K): δ = 143.3, 139.8, 132.2, 128.2, 128.0, 127.1.

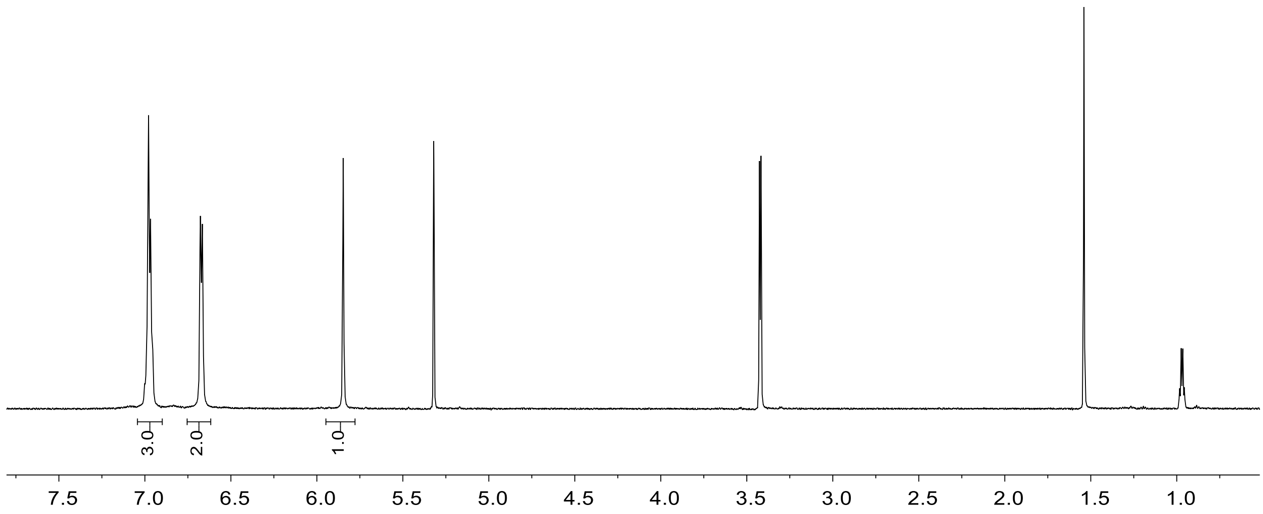

Figure S83. **^{1}H NMR** (600 MHz, CD_2Cl_2, 299 K) of the obtained polyphenyacetylene.

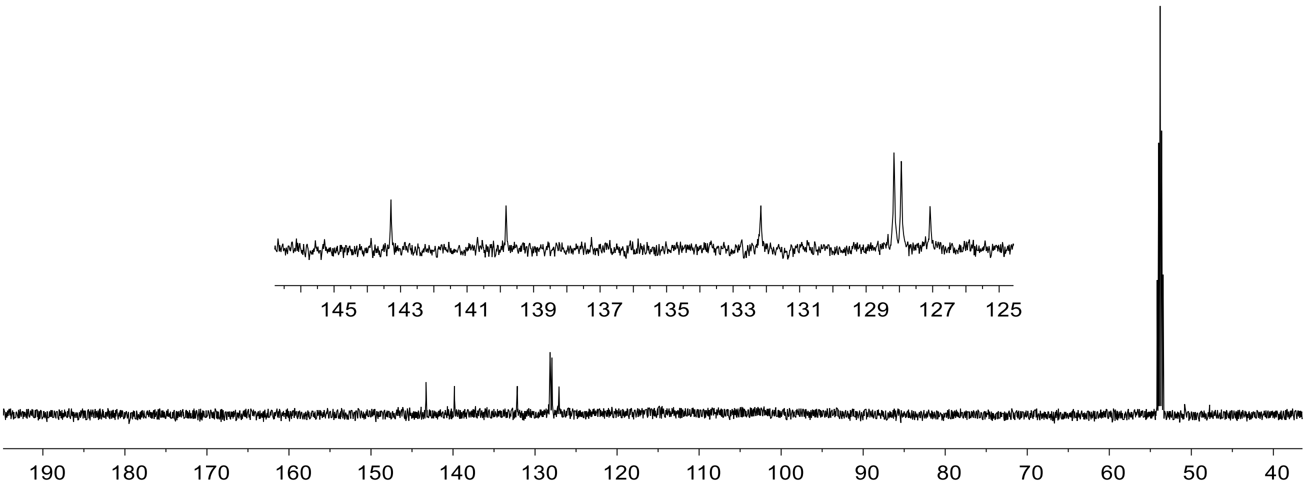

Figure S84. **$^{13}C\{^1H\}$ NMR** (151 MHz, CD_2Cl_2, 299 K) of the obtained polyphenyacetylene.

Entry 5: Polymerization of phenylacetylene in the presence of Rh complex 12 (0.05 mol%)

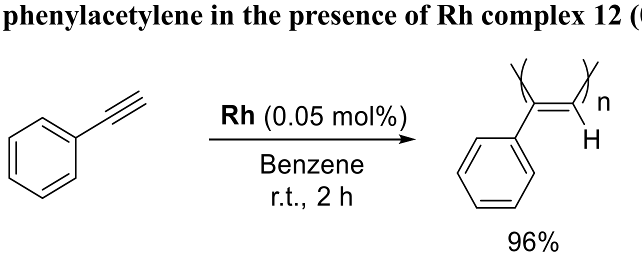

Scheme S22

In a glovebox with an argon atmosphere, compound **12** (Rh complex) (1.0 mg, 0.00125 mmol, 0.05 mol%) was dissolved in benzene (4 mL). Then, a solution of phenylacetylene (255.3 mg, 2.5 mmol) in benzene (6 mL) was gradually added. The mixture was stirred at room temperature for 2 hours. The obtained reaction mixture was washed with methanol (10 mL, 3 times) and then dried in vacuo at 50 °C to give a red solid (245.9 mg, 96%). **^{1}H NMR** (600 MHz, CD_2Cl_2, 299 K): δ = 6.97 (m, 3H, *m,p*-Ph), 6.67 (m, 2H, *o*-Ph), 5.84 (br, 1H, =CH).

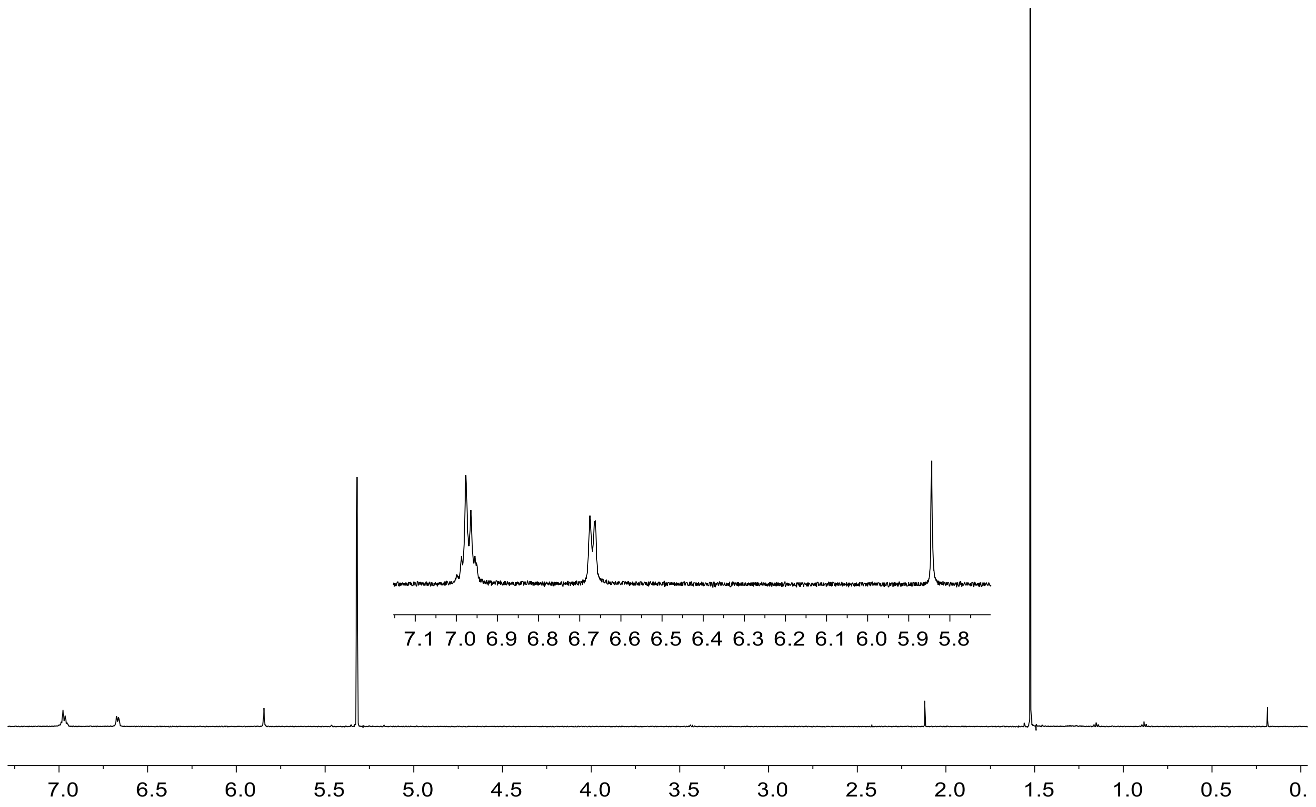

Figure S85. **^{1}H NMR** (600 MHz, CD_2Cl_2, 299 K) of the obtained polyphenylacetylene.

Entry 6: Polymerization of phenylacetylene in the presence of Rh complex 12 (0.025 mol%)

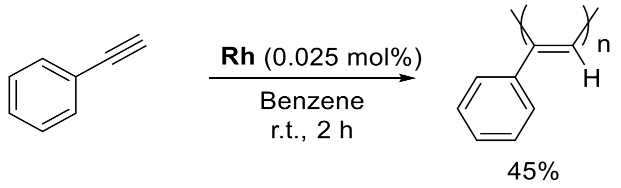

Scheme S23

In a glovebox with an argon atmosphere, compound **12** (Rh complex) (1.0 mg, 0.00125 mmol, 0.025 mol%) was dissolved in benzene (4 mL). Then, a solution of phenylacetylene (510.7 mg, 5.0 mmol) in benzene (6 mL) was gradually added. After addition, it was stirred at room temperature for 2 hours. The obtained reaction mixture was washed by methanol (10 mL, 3 times) and then dried in vacuo at 50 °C to give a red solid (228.2 mg, 45%).

Entry 7: Polymerization of phenylacetylene in the presence of Rh complex 12 (0.01 mol%)

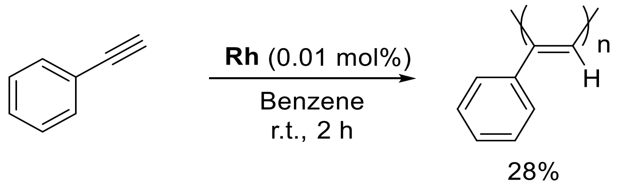

Scheme S24

In a glovebox with an argon atmosphere, compound **12** (Rh complex) (1.0 mg, 0.00125 mmol, 0.01 mol%) was dissolved in benzene (5 mL). Then, a solution of phenylacetylene (1276.7 mg, 12.5 mmol, 1 equiv.) in benzene (10 mL) was gradually added. After addition, it was stirred at room temperature for 2 hours. The obtained reaction mixture was washed with methanol (10 mL, 3 times) and then dried in vacuo at 50 °C to give a red solid (360.6 mg, 28%).

Entry 8: Polymerization of phenylacetylene in the presence of Rh complex 12 (0.1 mol%) in THF

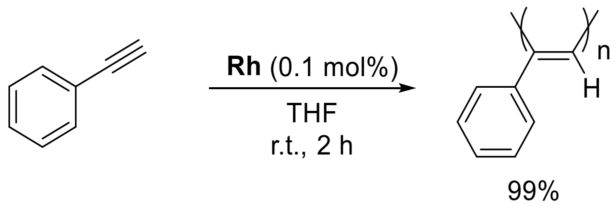

Scheme S25

In a glovebox with an argon atmosphere, compound **12** (Rh complex) (2.1 mg, 0.00125 mmol, 0.1 mol%) was dissolved in THF (4 mL). Then, a solution of phenylacetylene (255.3 mg, 2.5 mmol) in THF (6 mL) was gradually added. After addition, it was stirred at room temperature for 2 hours. The obtained reaction mixture was washed with methanol (10 mL, 3 times) and then dried in vacuo at 50 °C to give a red solid (252.1 mg, 99%).

Entry 9: Polymerization of (*p*-fluorophenyl)acetylene in the presence of Rh complex 12 (0.1 mol%)

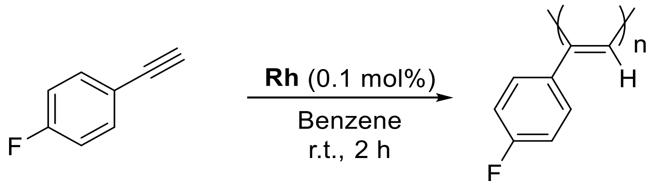

Scheme S26

In a glovebox with an argon atmosphere, compound **12** (Rh complex) (2.1 mg, 0.0025 mmol, 0.1 mol%) was dissolved in benzene (4 mL). Then, a solution of (*p*-fluorophenyl)acetylene (300.3 mg, 2.5 mmol, 1 equiv.) in benzene (6 mL) was gradually added. After addition, it was stirred at room temperature for 2 hours. The obtained reaction mixture was washed with methanol (10 mL, 3 times) and then dried in vacuo at 50 °C to give an orange solid (287.0 mg, 96%).

^{1}H NMR (600 MHz, CD_2Cl_2, 299 K): δ = 6.73 (m, 2H, *m*-Ph), 6.69 (m, 2H, *o*-Ph), 5.74 (s, 1H, -PhC=CH-).
^{19}F NMR (564 MHz, CD_2Cl_2, 299 K): δ = -115.4.

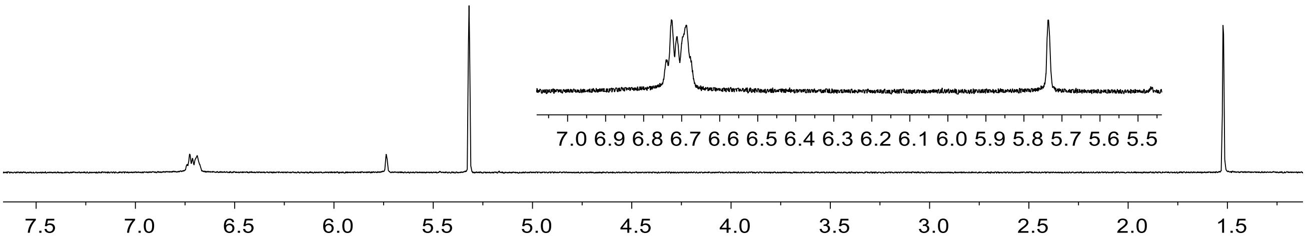

Figure S86. **^{1}H NMR** (600 MHz, CD_2Cl_2, 299 K) spectrum of the obtained poly(p-fluorophenylacetylene).

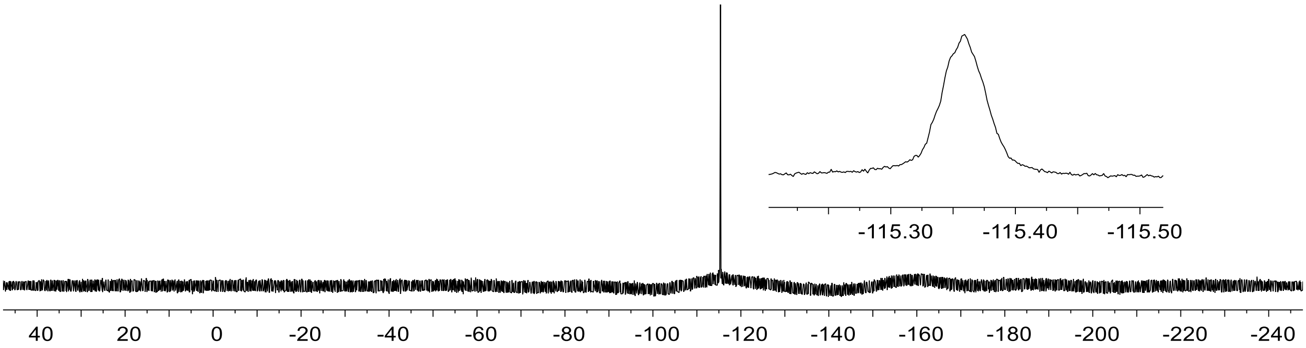

Figure S87. **^{19}F NMR** (564 MHz, CD_2Cl_2, 299 K) spectrum of the obtained poly(p-fluorophenylacetylene).

Entry 10: Polymerization of (*p*-methoxyphenyl)acetylene in the presence of Rh complex 12 (0.1 mol%)

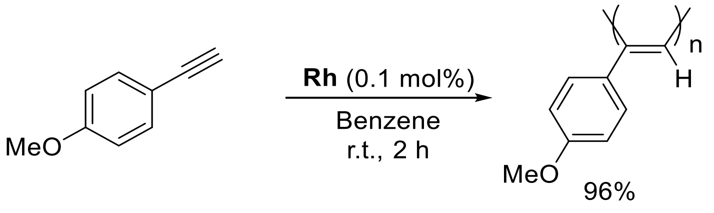

Scheme S27

In a glovebox with an argon atmosphere, compound **12** (Rh complex) (2.1 mg, 0.0025 mmol, 0.1 mol%) was dissolved in benzene (4 mL). Then, a solution of (*p*-methoxyphenyl)acetylene (330.4 mg, 2.5 mmol) in benzene (6 mL) was gradually added. After addition, it was stirred at room temperature for 2 hours. The obtained reaction mixture was washed with methanol (10 mL, 3 times) and then dried in vacuo at 50 °C to give orange solid (316.9 mg, 96%).

^{1}H NMR (600 MHz, CD_2Cl_2, 299 K): δ = [6.66, 6.49](each m, each 2H, Ph), 5.76 (s, 1H, =CH), 3.59 (s, 3H, OMe).

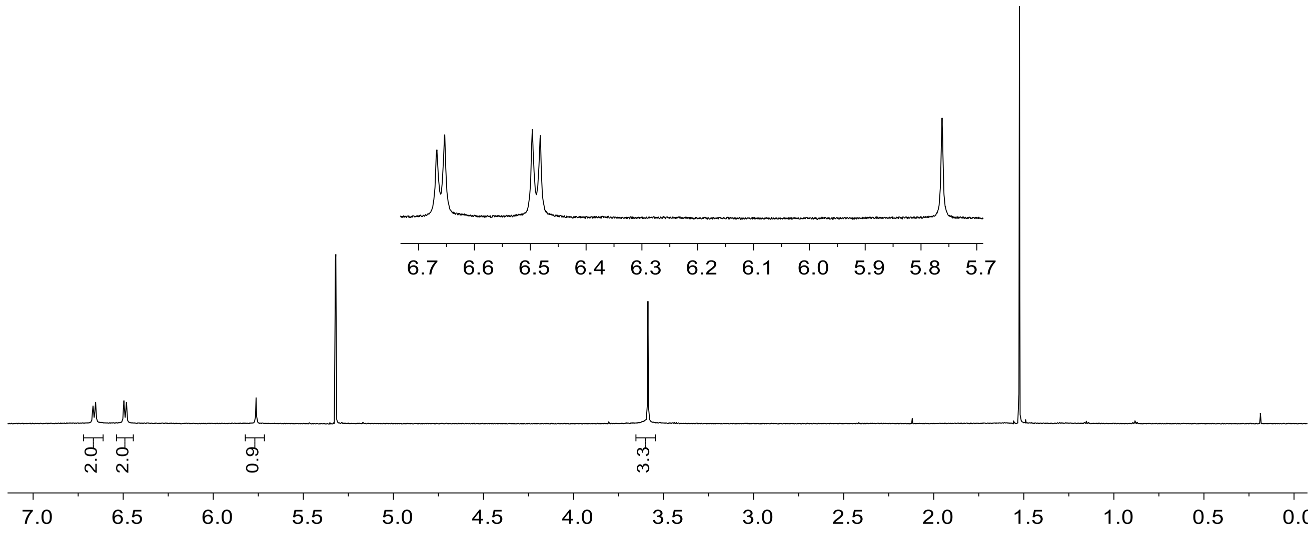

Figure S88. **^{1}H NMR** (600 MHz, CD_2Cl_2, 299 K) spectrum of the obtained poly(p-methoxyphenylacetylene).

MALDI-TOF: The compound was dissolved in $CHCl_3$ and mixed with a solution of DCTB in $CHCl_3$ to be co-crystallized on the MALDI target. Approx. 1000 laser shots were added up to the depicted spectrum.

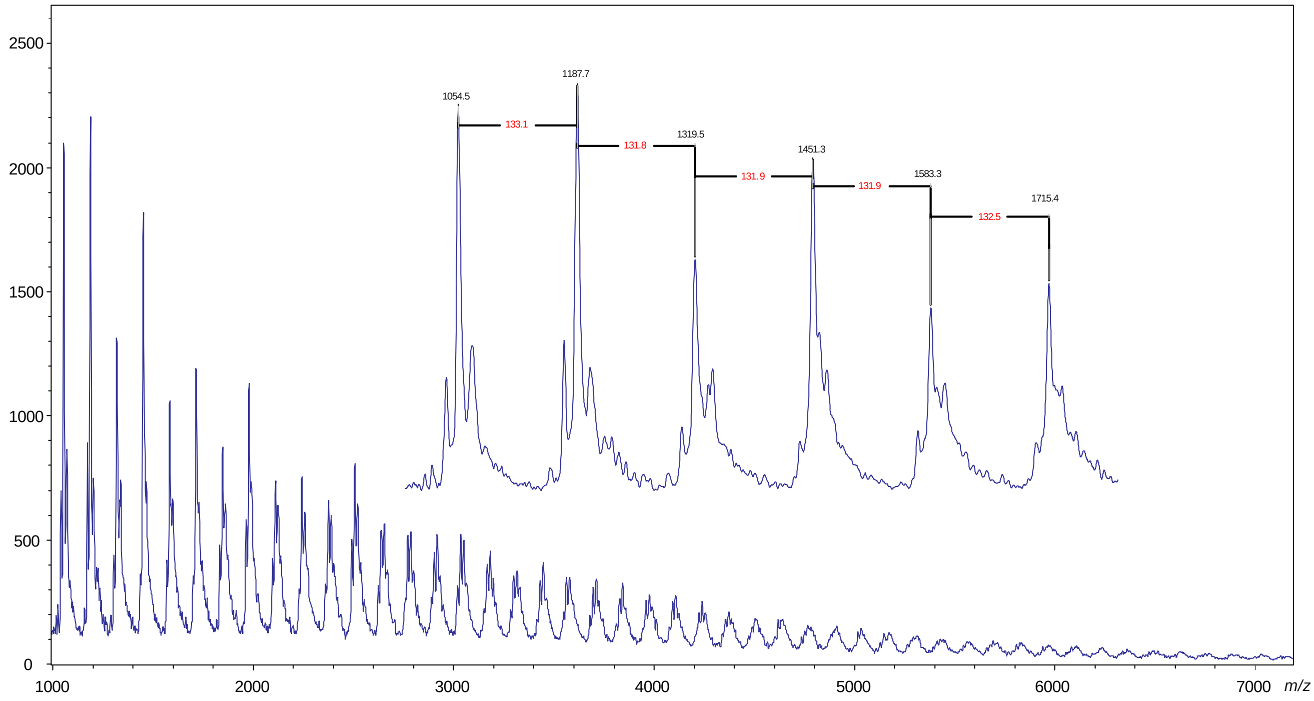

Figure S89. MALDI-TOF spectrum of the obtained poly(p-methoxyphenylacetylene) with DCTB as matrix.

Entry 11: Polymerization of 1-pentyne in the presence of Rh complex 12 (0.1 mol%)

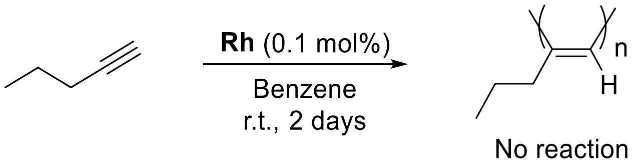

Scheme S28

In a glovebox with an argon atmosphere, a mixture of compound **12** (Rh complex) (4.7 mg, 0.005 mmol, 0.1 mol%) and 1-pentyne (34.1 mg, 0.5 mmol, 1 eq.) was dissolved in C_6D_6 (1 mL). Then the solution was stirred at room temperature for 2 days. The obtained reaction mixture was characterized by ^1H NMR spectroscopy: no conversion of 1-pentyne was observed.

Entry 12: Polymerization of 1-ethynylcyclohexene in the presence of Rh complex 12 (0.1 mol%)

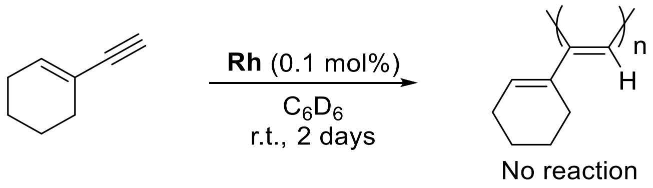

Scheme S29

In a glovebox with an argon atmosphere, a mixture of compound **12** (Rh complex) (2.1 mg, 0.0025 mmol, 0.1 mol%) and 1-ethynylcyclohexene (265.4.1 mg, 0.25 mmol, 1 eq.) was dissolved in C_6D_6 (2 mL). Then it was stirred at room temperature for 2 days. The obtained mixture was characterized by ^1H NMR spectroscopy: no conversion of 1-pentyne was observed.
